# Supplementary material for: Opposition-based learning techniques in metaheuristics: classification, comparison, and convergence analysis
Source: PeerJ Comput Sci. 2025 Jul 15;11:e2935. doi: 10.7717/peerj-cs.2935 (PMC12453659; doi:10.7717/peerj-cs.2935)
Supplement: Supplemental Information 1 [file peerj-cs-11-2935-s001.pdf]

A Appendix

This appendix provides a comprehensive analysis of the performance metrics for the five selected MAs and their OBL variants, evaluated across 12 benchmark functions. The algorithms are assessed on two different dimensionalities: 10 and 20. Detailed metrics, including Min, Max, Mean, Std, ET, and NFEs, are presented in Tables 1 to 10. Furthermore, Tables 11 to 15 summarize the Friedman test results based on mean fitness values. Additionally, Figures 1 to 30 depict the convergence curves of various algorithmic variants across iterations, offering insights into their solution quality, convergence speed, and the trade-off between exploration and exploitation.

Table 1: Performance metrics for DE variants across 12 Functions ($D = 10$)

			F_1	F_2	F_3	F_4	F_5	F_6	F_7	F_8	F_9	F_{10}	F_{11}	F_{12}
DE		Min	3.00E+02	4.00E+02	6.00E+02	8.03E+02	9.00E+02	1.82E+03	2.00E+03	2.20E+03	2.53E+03	2.50E+03	2.60E+03	2.86E+03
		Max	3.00E+02	4.09E+02	6.00E+02	8.36E+02	9.00E+02	3.77E+03	2.02E+03	2.22E+03	2.53E+03	2.61E+03	3.00E+03	2.87E+03
		Mean	3.00E+02	4.07E+02	6.00E+02	8.22E+02	9.00E+02	2.21E+03	2.01E+03	2.21E+03	2.53E+03	2.51E+03	2.62E+03	2.86E+03
		STD	6.39E-12	7.45E+00	4.15E-14	2.37E+01	0.00E+00	6.39E+02	1.67E+01	1.45E+01	2.29E+02	1.16E+02	8.27E+01	1.63E+02
		ET	3.86E-01	3.85E-01	4.49E-01	3.99E-01	4.08E-01	3.83E-01	4.68E-01	4.96E-01	4.59E-01	4.51E-01	5.23E-01	5.28E-01
		NFEs	5.00E+04	5.00E+04	5.00E+04	5.00E+04	5.00E+04	5.00E+04	5.00E+04	5.00E+04	5.00E+04	5.00E+04	5.00E+04	5.00E+04
BOBL-DE	IP	Min	3.00E+02	4.00E+02	6.00E+02	8.04E+02	9.00E+02	1.82E+03	2.00E+03	2.20E+03	2.53E+03	2.50E+03	2.60E+03	2.86E+03
		Max	3.00E+02	4.09E+02	6.00E+02	8.36E+02	9.00E+02	4.71E+03	2.02E+03	2.23E+03	2.53E+03	2.61E+03	3.00E+03	2.86E+03
		Mean	3.00E+02	4.06E+02	6.00E+02	8.19E+02	9.00E+02	2.34E+03	2.01E+03	2.21E+03	2.53E+03	2.52E+03	2.62E+03	2.86E+03
		STD	1.27E-12	6.11E+00	4.64E-14	2.16E+01	0.00E+00	9.55E+02	1.48E+01	1.58E+01	2.29E+02	1.29E+02	7.80E+01	1.63E+02
		ET	3.80E-01	3.79E-01	4.39E-01	3.93E-01	4.03E-01	3.77E-01	4.64E-01	4.90E-01	4.53E-01	4.42E-01	5.14E-01	5.15E-01
		NFEs	5.01E+04	5.01E+04	5.01E+04	5.01E+04	5.01E+04	5.01E+04	5.01E+04	5.01E+04	5.01E+04	5.01E+04	5.01E+04	5.01E+04
	SP	Min	3.00E+02	4.00E+02	6.00E+02	8.04E+02	9.00E+02	1.81E+03	2.00E+03	2.20E+03	2.53E+03	2.50E+03	2.60E+03	2.86E+03
		Max	3.00E+02	4.09E+02	6.00E+02	8.37E+02	9.00E+02	5.03E+03	2.02E+03	2.22E+03	2.53E+03	2.61E+03	3.00E+03	2.87E+03
		Mean	3.00E+02	4.06E+02	6.00E+02	8.22E+02	9.00E+02	2.17E+03	2.01E+03	2.22E+03	2.53E+03	2.51E+03	2.65E+03	2.86E+03
		STD	1.65E-11	6.33E+00	5.87E-14	2.42E+01	0.00E+00	8.29E+02	1.72E+01	1.80E+01	2.29E+02	1.21E+02	1.32E+02	1.63E+02
		ET	4.55E-01	4.40E-01	5.16E-01	4.56E-01	4.70E-01	4.06E-01	5.50E-01	5.84E-01	5.24E-01	5.09E-01	6.32E-01	6.15E-01
		NFEs	6.51E+04	6.50E+04	6.48E+04	6.49E+04	6.53E+04	6.48E+04	6.49E+04	6.51E+04	6.52E+04	6.48E+04	6.53E+04	6.53E+04
	IP-SP	Min	3.00E+02	4.00E+02	6.00E+02	8.05E+02	9.00E+02	1.82E+03	2.00E+03	2.20E+03	2.53E+03	2.50E+03	2.60E+03	2.86E+03
		Max	3.00E+02	4.09E+02	6.00E+02	8.35E+02	9.00E+02	4.35E+03	2.02E+03	2.22E+03	2.53E+03	2.61E+03	3.00E+03	2.87E+03
		Mean	3.00E+02	4.07E+02	6.00E+02	8.23E+02	9.00E+02	2.22E+03	2.01E+03	2.21E+03	2.53E+03	2.50E+03	2.63E+03	2.86E+03
		STD	1.84E-11	7.40E+00	5.49E-14	2.45E+01	0.00E+00	6.80E+02	1.54E+01	1.56E+01	2.29E+02	1.06E+02	1.03E+02	1.63E+02
		ET	4.62E-01	4.58E-01	5.66E-01	4.73E-01	4.89E-01	4.51E-01	5.74E-01	6.12E-01	5.56E-01	5.39E-01	6.33E-01	6.40E-01
		NFEs	6.50E+04	6.51E+04	6.51E+04	6.53E+04	6.50E+04	6.48E+04	6.52E+04	6.50E+04	6.51E+04	6.51E+04	6.50E+04	6.52E+04

Table 1 continued from previous page

			F_1	F_2	F_3	F_4	F_5	F_6	F_7	F_8	F_9	F_{10}	F_{11}	F_{12}
COOBL-DE	IP	Min	3.00E+02	4.00E+02	6.00E+02	8.04E+02	9.00E+02	1.81E+03	2.00E+03	2.20E+03	2.53E+03	2.50E+03	2.60E+03	2.86E+03
		Max	3.00E+02	4.09E+02	6.00E+02	8.35E+02	9.00E+02	6.93E+03	2.14E+03	2.22E+03	2.53E+03	2.61E+03	3.00E+03	2.86E+03
		Mean	3.00E+02	4.07E+02	6.00E+02	8.23E+02	9.00E+02	2.32E+03	2.02E+03	2.21E+03	2.53E+03	2.51E+03	2.64E+03	2.86E+03
		STD	5.54E-12	7.62E+00	4.64E-14	2.48E+01	0.00E+00	1.15E+03	3.01E+01	1.69E+01	2.29E+02	1.20E+02	1.26E+02	1.63E+02
		ET	3.76E-01	3.78E-01	4.41E-01	3.92E-01	4.09E-01	3.80E-01	4.69E-01	4.97E-01	4.60E-01	4.46E-01	5.15E-01	5.17E-01
		NFEs	5.01E+04	5.01E+04	5.01E+04	5.01E+04	5.01E+04	5.01E+04	5.01E+04	5.01E+04	5.01E+04	5.01E+04	5.01E+04	5.01E+04
	SP	Min	3.00E+02	4.00E+02	6.00E+02	8.04E+02	9.00E+02	1.82E+03	2.02E+03	2.22E+03	2.49E+03	2.50E+03	2.60E+03	2.85E+03
		Max	3.09E+04	4.09E+02	6.00E+02	8.70E+02	9.02E+02	3.29E+06	2.04E+03	2.24E+03	2.54E+03	2.65E+03	2.90E+03	2.90E+03
		Mean	3.16E+03	4.04E+02	6.00E+02	8.38E+02	9.00E+02	2.41E+05	2.02E+03	2.23E+03	2.51E+03	2.52E+03	2.63E+03	2.87E+03
		STD	7.87E+03	4.88E+00	2.09E-02	4.12E+01	4.61E-01	7.03E+05	2.49E+01	2.66E+01	2.08E+02	1.23E+02	7.76E+01	1.67E+02
		ET	4.38E-01	4.32E-01	5.28E-01	8.19E-01	1.85E+00	1.94E+00	9.56E-01	5.65E-01	5.23E-01	5.12E-01	6.02E-01	6.04E-01
		NFEs	6.50E+04	6.49E+04	6.52E+04	6.51E+04	6.50E+04	6.49E+04	6.49E+04	6.50E+04	6.51E+04	6.51E+04	6.54E+04	6.50E+04
	IP-SP	Min	3.00E+02	4.00E+02	6.00E+02	8.08E+02	9.00E+02	1.83E+03	2.00E+03	2.20E+03	2.49E+03	2.50E+03	2.60E+03	2.85E+03
		Max	3.62E+04	4.08E+02	6.00E+02	8.70E+02	1.46E+03	1.58E+07	2.04E+03	2.33E+03	2.61E+03	3.53E+03	3.00E+03	2.90E+03
		Mean	3.76E+03	4.05E+02	6.00E+02	8.38E+02	9.19E+02	9.37E+05	2.02E+03	2.23E+03	2.51E+03	2.55E+03	2.63E+03	2.87E+03
		STD	9.07E+03	5.05E+00	3.51E-02	4.16E+01	1.02E+02	3.05E+06	2.31E+01	3.37E+01	2.15E+02	2.40E+02	9.23E+01	1.68E+02
		ET	4.21E-01	4.20E-01	5.05E-01	4.51E-01	4.68E-01	4.44E-01	5.50E-01	6.10E-01	5.53E-01	5.34E-01	6.36E-01	6.52E-01
		NFEs	6.51E+04	6.49E+04	6.53E+04	6.49E+04	6.51E+04	6.52E+04	6.49E+04	6.51E+04	6.50E+04	6.53E+04	6.50E+04	6.49E+04

Table 1 continued from previous page

			F_1	F_2	F_3	F_4	F_5	F_6	F_7	F_8	F_9	F_{10}	F_{11}	F_{12}
GOBL-DE	IP	Min	3.00E+02	4.04E+02	6.00E+02	8.04E+02	9.00E+02	1.80E+03	2.00E+03	2.20E+03	2.53E+03	2.50E+03	2.60E+03	2.86E+03
		Max	3.00E+02	4.09E+02	6.00E+02	8.33E+02	9.00E+02	3.69E+03	2.02E+03	2.22E+03	2.53E+03	2.61E+03	2.75E+03	2.86E+03
		Mean	3.00E+02	4.07E+02	6.00E+02	8.22E+02	9.00E+02	2.09E+03	2.01E+03	2.21E+03	2.53E+03	2.51E+03	2.61E+03	2.86E+03
		STD	4.04E-10	7.66E+00	3.60E-14	2.35E+01	0.00E+00	5.35E+02	1.40E+01	1.54E+01	2.29E+02	1.11E+02	2.75E+01	1.63E+02
		ET	5.11E-01	5.07E-01	5.89E-01	5.30E-01	5.42E-01	5.06E-01	6.16E-01	6.51E-01	6.12E-01	5.97E-01	1.46E+01	7.18E-01
		NFEs	5.01E+04	5.01E+04	5.01E+04	5.01E+04	5.01E+04	5.01E+04	5.01E+04	5.01E+04	5.01E+04	5.01E+04	5.01E+04	5.01E+04
	SP	Min	3.00E+02	4.00E+02	6.00E+02	8.03E+02	9.00E+02	1.82E+03	2.00E+03	2.20E+03	2.53E+03	2.50E+03	2.60E+03	2.86E+03
		Max	3.00E+02	4.09E+02	6.00E+02	8.36E+02	9.00E+02	3.96E+03	2.02E+03	2.22E+03	2.53E+03	2.61E+03	2.75E+03	2.86E+03
		Mean	3.00E+02	4.06E+02	6.00E+02	8.21E+02	9.00E+02	2.13E+03	2.01E+03	2.21E+03	2.53E+03	2.51E+03	2.61E+03	2.86E+03
		STD	1.89E-12	6.57E+00	5.87E-14	2.32E+01	0.00E+00	6.06E+02	1.63E+01	1.52E+01	2.29E+02	1.11E+02	2.75E+01	1.63E+02
		ET	5.56E-01	5.61E-01	6.67E-01	5.94E-01	6.18E-01	3.33E+00	7.18E-01	7.55E-01	6.88E-01	6.79E-01	7.99E-01	7.91E-01
		NFEs	6.51E+04	6.50E+04	6.51E+04	6.52E+04	6.48E+04	6.52E+04	6.50E+04	6.52E+04	6.50E+04	6.53E+04	6.50E+04	6.51E+04
	IP-SP	Min	3.00E+02	4.04E+02	6.00E+02	8.02E+02	9.00E+02	1.82E+03	2.00E+03	2.20E+03	2.53E+03	2.50E+03	2.60E+03	2.86E+03
		Max	3.00E+02	4.09E+02	6.00E+02	8.36E+02	9.00E+02	2.97E+03	2.02E+03	2.22E+03	2.53E+03	2.50E+03	2.75E+03	2.87E+03
		Mean	3.00E+02	4.06E+02	6.00E+02	8.21E+02	9.00E+02	2.06E+03	2.01E+03	2.21E+03	2.53E+03	2.50E+03	2.61E+03	2.86E+03
		STD	1.24E-11	6.62E+00	4.64E-14	2.33E+01	0.00E+00	4.11E+02	1.54E+01	1.47E+01	2.29E+02	1.00E+02	3.88E+01	1.63E+02
		ET	5.56E-01	5.65E-01	6.71E-01	6.01E-01	6.46E-01	5.65E-01	7.07E-01	4.14E+00	7.07E-01	6.71E-01	7.87E-01	7.93E-01
		NFEs	6.52E+04	6.51E+04	6.49E+04	6.51E+04	6.50E+04	6.52E+04	6.51E+04	6.56E+04	6.52E+04	6.50E+04	6.50E+04	6.51E+04

Table 1 continued from previous page

			F_1	F_2	F_3	F_4	F_5	F_6	F_7	F_8	F_9	F_{10}	F_{11}	F_{12}
QOBL-DE	IP	Min	3.00E+02	4.04E+02	6.00E+02	8.02E+02	9.00E+02	1.81E+03	2.00E+03	2.20E+03	2.53E+03	2.50E+03	2.60E+03	2.86E+03
		Max	3.00E+02	4.09E+02	6.00E+02	8.38E+02	9.00E+02	5.39E+03	2.02E+03	2.23E+03	2.53E+03	2.61E+03	2.75E+03	2.86E+03
		Mean	3.00E+02	4.07E+02	6.00E+02	8.20E+02	9.00E+02	2.30E+03	2.01E+03	2.21E+03	2.53E+03	2.51E+03	2.61E+03	2.86E+03
		STD	9.29E-13	6.94E+00	5.08E-14	2.30E+01	0.00E+00	9.79E+02	1.29E+01	1.58E+01	2.29E+02	1.15E+02	2.75E+01	1.63E+02
		ET	3.84E-01	4.83E-01	5.15E-01	5.66E-01	4.94E-01	5.49E-01	5.48E-01	4.87E-01	4.51E-01	4.36E-01	5.10E-01	5.24E-01
		NFEs	5.01E+04	5.01E+04	5.01E+04	5.01E+04	5.01E+04	5.01E+04	5.01E+04	5.01E+04	5.01E+04	5.01E+04	5.01E+04	5.01E+04
	SP	Min	3.00E+02	4.00E+02	6.00E+02	8.04E+02	9.00E+02	1.81E+03	2.00E+03	2.20E+03	2.53E+03	2.50E+03	2.60E+03	2.86E+03
		Max	3.00E+02	4.09E+02	6.00E+02	8.35E+02	9.00E+02	4.83E+03	2.02E+03	2.22E+03	2.53E+03	2.61E+03	2.75E+03	2.86E+03
		Mean	3.00E+02	4.05E+02	6.00E+02	8.22E+02	9.00E+02	2.15E+03	2.01E+03	2.21E+03	2.53E+03	2.51E+03	2.61E+03	2.86E+03
		STD	1.11E-12	5.64E+00	2.07E-06	2.39E+01	0.00E+00	7.04E+02	1.29E+01	1.71E+01	2.29E+02	1.20E+02	2.75E+01	1.63E+02
		ET	6.37E-01	6.62E-01	6.95E-01	6.38E-01	6.52E-01	6.15E-01	7.43E-01	7.76E-01	7.45E-01	7.06E-01	8.07E-01	8.05E-01
		NFEs	6.48E+04	6.51E+04	6.52E+04	6.51E+04	6.51E+04	6.51E+04	6.51E+04	6.47E+04	6.48E+04	6.52E+04	6.51E+04	6.51E+04
	IP-SP	Min	3.00E+02	4.00E+02	6.00E+02	8.03E+02	9.00E+02	1.82E+03	2.00E+03	2.20E+03	2.53E+03	2.50E+03	2.60E+03	2.86E+03
		Max	3.00E+02	4.09E+02	6.00E+02	8.36E+02	9.00E+02	4.00E+03	2.02E+03	2.22E+03	2.53E+03	2.50E+03	2.75E+03	2.87E+03
		Mean	3.00E+02	4.07E+02	6.00E+02	8.19E+02	9.00E+02	2.22E+03	2.01E+03	2.21E+03	2.53E+03	2.50E+03	2.61E+03	2.86E+03
		STD	7.71E-13	7.04E+00	6.56E-14	2.18E+01	0.00E+00	7.38E+02	1.54E+01	1.28E+01	2.29E+02	1.00E+02	2.75E+01	1.63E+02
		ET	5.46E-01	5.53E-01	6.33E-01	5.71E-01	5.92E-01	5.66E-01	6.79E-01	7.04E-01	6.57E-01	6.37E-01	7.31E-01	7.32E-01
		NFEs	6.48E+04	6.51E+04	6.52E+04	6.51E+04	6.51E+04	6.50E+04	6.53E+04	6.50E+04	6.52E+04	6.52E+04	6.50E+04	6.49E+04

Table 1 continued from previous page

			F_1	F_2	F_3	F_4	F_5	F_6	F_7	F_8	F_9	F_{10}	F_{11}	F_{12}
QROBL-DE	IP	Min	3.00E+02	4.00E+02	6.00E+02	8.04E+02	9.00E+02	1.82E+03	2.00E+03	2.20E+03	2.53E+03	2.50E+03	2.60E+03	2.86E+03
		Max	3.00E+02	4.09E+02	6.00E+02	8.35E+02	9.00E+02	3.31E+03	2.02E+03	2.22E+03	2.53E+03	2.61E+03	2.75E+03	2.87E+03
		Mean	3.00E+02	4.06E+02	6.00E+02	8.22E+02	9.00E+02	2.17E+03	2.01E+03	2.21E+03	2.53E+03	2.52E+03	2.61E+03	2.86E+03
		STD	8.41E-13	6.70E+00	4.64E-14	2.42E+01	0.00E+00	5.96E+02	1.48E+01	1.35E+01	2.29E+02	1.25E+02	2.75E+01	1.63E+02
		ET	4.12E-01	4.31E-01	4.64E-01	4.17E-01	4.06E-01	3.83E-01	4.70E-01	4.99E-01	4.59E-01	4.44E-01	5.35E-01	2.20E+00
		NFEs	5.01E+04	5.01E+04	5.01E+04	5.01E+04	5.01E+04	5.01E+04	5.01E+04	5.01E+04	5.01E+04	5.01E+04	5.01E+04	5.01E+04
	SP	Min	3.00E+02	4.04E+02	6.00E+02	8.04E+02	9.00E+02	1.81E+03	2.00E+03	2.20E+03	2.53E+03	2.50E+03	2.60E+03	2.86E+03
		Max	3.00E+02	4.09E+02	6.00E+02	8.29E+02	9.00E+02	4.65E+03	2.02E+03	2.22E+03	2.53E+03	2.61E+03	2.60E+03	2.87E+03
		Mean	3.00E+02	4.06E+02	6.00E+02	8.12E+02	9.00E+02	2.29E+03	2.01E+03	2.21E+03	2.53E+03	2.53E+03	2.60E+03	2.86E+03
		STD	0.00E+00	6.62E+00	4.15E-14	1.46E+01	0.00E+00	9.00E+02	1.29E+01	1.38E+01	2.29E+02	1.33E+02	2.49E-13	1.63E+02
		ET	5.71E-01	5.71E-01	6.58E-01	6.07E-01	6.21E-01	5.89E-01	7.40E-01	7.36E-01	6.79E-01	6.73E-01	7.77E-01	7.63E-01
		NFEs	6.48E+04	6.50E+04	6.53E+04	6.49E+04	6.50E+04	6.48E+04	6.52E+04	6.50E+04	6.52E+04	6.49E+04	6.53E+04	6.52E+04
	IP-SP	Min	3.00E+02	4.00E+02	6.00E+02	8.03E+02	9.00E+02	1.81E+03	2.00E+03	2.20E+03	2.53E+03	2.50E+03	2.60E+03	2.86E+03
		Max	3.00E+02	4.09E+02	6.00E+02	8.24E+02	9.00E+02	4.18E+03	2.02E+03	2.23E+03	2.53E+03	2.61E+03	2.60E+03	2.86E+03
		Mean	3.00E+02	4.07E+02	6.00E+02	8.10E+02	9.00E+02	2.21E+03	2.01E+03	2.21E+03	2.53E+03	2.53E+03	2.60E+03	2.86E+03
		STD	0.00E+00	7.04E+00	4.64E-14	1.17E+01	0.00E+00	6.66E+02	1.12E+01	1.39E+01	2.29E+02	1.33E+02	2.03E-13	1.62E+02
		ET	5.76E-01	5.69E-01	6.61E-01	6.25E-01	4.95E+02	6.11E-01	7.41E-01	7.16E-01	6.68E-01	6.50E-01	7.38E-01	7.36E-01
		NFEs	6.51E+04	6.51E+04	6.50E+04	6.51E+04	6.49E+04	6.51E+04	6.50E+04	6.49E+04	6.52E+04	6.52E+04	6.51E+04	6.49E+04

Table 2: Performance metrics for DE variants across 12 Functions ($D = 20$)

			F_1	F_2	F_3	F_4	F_5	F_6	F_7	F_8	F_9	F_{10}	F_{11}	F_{12}
DE		Min	4.64E+03	4.45E+02	6.00E+02	8.90E+02	9.00E+02	3.85E+03	2.03E+03	2.23E+03	2.48E+03	2.50E+03	2.90E+03	2.93E+03
		Max	1.55E+04	4.49E+02	6.00E+02	9.24E+02	9.00E+02	4.01E+05	2.05E+03	2.24E+03	2.48E+03	5.03E+03	3.00E+03	2.98E+03
		Mean	9.85E+03	4.49E+02	6.00E+02	9.06E+02	9.00E+02	4.38E+04	2.03E+03	2.23E+03	2.48E+03	2.86E+03	2.93E+03	2.94E+03
		STD	9.92E+03	4.85E+01	2.65E-05	1.07E+02	7.05E-12	8.05E+04	3.43E+01	3.17E+01	1.81E+02	8.59E+02	3.37E+02	2.42E+02
		ET	4.11E-01	4.20E-01	5.44E-01	4.44E-01	4.63E-01	4.15E-01	5.80E-01	6.14E-01	6.05E-01	6.01E-01	1.95E+01	7.00E-01
		NFEs	5.00E+04	5.00E+04	5.00E+04	5.00E+04	5.00E+04	5.00E+04	5.00E+04	5.00E+04	5.00E+04	5.00E+04	5.00E+04	5.00E+04
BOBL-DE	IP	Min	5.04E+03	4.45E+02	6.00E+02	8.83E+02	9.00E+02	4.03E+03	2.02E+03	2.23E+03	2.48E+03	2.50E+03	2.90E+03	2.93E+03
		Max	1.83E+04	4.69E+02	6.01E+02	9.24E+02	9.00E+02	4.65E+04	2.05E+03	2.24E+03	2.48E+03	5.12E+03	3.00E+03	2.96E+03
		Mean	9.62E+03	4.49E+02	6.00E+02	9.07E+02	9.00E+02	2.37E+04	2.03E+03	2.23E+03	2.48E+03	2.96E+03	2.93E+03	2.94E+03
		STD	9.71E+03	4.91E+01	1.03E-01	1.07E+02	5.90E-11	2.39E+04	3.26E+01	3.24E+01	1.81E+02	9.81E+02	3.37E+02	2.41E+02
		ET	4.10E-01	4.99E-01	7.93E-01	5.83E-01	6.05E-01	5.45E-01	7.89E-01	7.99E-01	7.91E-01	6.99E-01	9.12E-01	1.26E+00
		NFEs	5.01E+04	5.01E+04	5.01E+04	5.01E+04	5.01E+04	5.01E+04	5.01E+04	5.01E+04	5.01E+04	5.01E+04	5.01E+04	5.01E+04
	SP	Min	4.28E+03	4.45E+02	6.00E+02	8.86E+02	9.00E+02	4.47E+03	2.02E+03	2.23E+03	2.48E+03	2.50E+03	2.60E+03	2.90E+03
		Max	2.10E+04	4.49E+02	6.00E+02	9.21E+02	9.00E+02	1.38E+05	2.05E+03	2.24E+03	2.48E+03	4.48E+03	3.00E+03	2.95E+03
		Mean	9.43E+03	4.48E+02	6.00E+02	9.06E+02	9.00E+02	3.00E+04	2.03E+03	2.23E+03	2.48E+03	2.78E+03	2.91E+03	2.93E+03
		STD	9.98E+03	4.84E+01	2.04E-05	1.07E+02	7.95E-12	3.69E+04	3.26E+01	3.13E+01	1.81E+02	7.13E+02	3.21E+02	2.35E+02
		ET	4.27E-01	5.14E-01	7.98E-01	6.56E-01	5.69E-01	6.09E-01	7.32E-01	7.94E-01	7.30E-01	6.60E-01	8.19E-01	8.90E-01
		NFEs	6.50E+04	6.51E+04	6.48E+04	6.50E+04	6.52E+04	6.51E+04	6.51E+04	6.51E+04	6.50E+04	6.52E+04	6.49E+04	6.52E+04
	IP-SP	Min	3.30E+03	4.45E+02	6.00E+02	8.85E+02	9.00E+02	3.40E+03	2.03E+03	2.23E+03	2.48E+03	2.50E+03	2.90E+03	2.90E+03
		Max	1.80E+04	4.49E+02	6.00E+02	9.24E+02	9.00E+02	6.98E+04	2.04E+03	2.24E+03	2.48E+03	4.40E+03	3.00E+03	2.94E+03
		Mean	1.05E+04	4.48E+02	6.00E+02	9.07E+02	9.00E+02	2.51E+04	2.03E+03	2.23E+03	2.48E+03	2.97E+03	2.93E+03	2.91E+03
		STD	1.08E+04	4.83E+01	2.11E-05	1.07E+02	6.98E-12	2.61E+04	3.29E+01	3.23E+01	1.81E+02	8.67E+02	3.30E+02	2.07E+02
		ET	5.19E-01	5.25E-01	7.17E-01	5.32E-01	5.55E-01	4.91E-01	7.11E-01	7.67E-01	7.39E-01	6.72E-01	8.42E-01	8.75E-01
		NFEs	6.52E+04	6.53E+04	6.52E+04	6.52E+04	6.50E+04	6.50E+04	6.51E+04	6.52E+04	6.51E+04	6.51E+04	6.52E+04	6.51E+04

Table 2 continued from previous page

			F_1	F_2	F_3	F_4	F_5	F_6	F_7	F_8	F_9	F_{10}	F_{11}	F_{12}
COOBL-DE	IP	Min	6.33E+03	4.45E+02	6.00E+02	8.79E+02	9.00E+02	6.93E+03	2.02E+03	2.23E+03	2.48E+03	2.50E+03	2.90E+03	2.94E+03
		Max	1.81E+04	4.49E+02	6.00E+02	9.21E+02	9.00E+02	1.35E+05	2.05E+03	2.24E+03	2.48E+03	4.57E+03	3.00E+03	3.01E+03
		Mean	1.07E+04	4.48E+02	6.00E+02	9.05E+02	9.00E+02	3.37E+04	2.03E+03	2.23E+03	2.48E+03	3.08E+03	2.92E+03	2.95E+03
		STD	1.10E+04	4.84E+01	1.91E-05	1.05E+02	6.94E-12	4.11E+04	3.21E+01	3.13E+01	1.81E+02	1.02E+03	3.26E+02	2.50E+02
		ET	4.05E-01	4.15E-01	5.43E-01	4.47E-01	4.63E-01	4.14E-01	5.84E-01	6.15E-01	6.01E-01	8.37E+00	7.34E-01	7.03E-01
		NFEs	5.01E+04	5.01E+04	5.01E+04	5.01E+04	5.01E+04	5.01E+04	5.01E+04	5.01E+04	5.01E+04	5.01E+04	5.01E+04	5.01E+04
	SP	Min	3.00E+02	4.00E+02	6.00E+02	8.22E+02	9.00E+02	2.03E+03	2.02E+03	2.22E+03	2.47E+03	2.50E+03	2.60E+03	2.90E+03
		Max	1.06E+05	4.81E+02	6.05E+02	9.97E+02	3.49E+03	4.34E+07	2.29E+03	2.35E+03	2.48E+03	5.25E+03	3.35E+03	2.90E+03
		Mean	3.71E+04	4.30E+02	6.00E+02	8.95E+02	1.05E+03	2.84E+06	2.09E+03	2.24E+03	2.48E+03	3.47E+03	2.93E+03	2.90E+03
		STD	4.97E+04	3.54E+01	9.54E-01	1.05E+02	5.01E+02	9.53E+06	1.08E+02	5.49E+01	1.77E+02	1.42E+03	3.48E+02	2.00E+02
		ET	4.66E-01	4.65E-01	6.49E-01	5.20E-01	5.27E-01	4.79E-01	6.90E-01	7.41E-01	7.10E-01	6.35E-01	8.18E-01	8.82E-01
		NFEs	6.51E+04	6.48E+04	6.52E+04	6.49E+04	6.51E+04	6.50E+04	6.51E+04	6.49E+04	6.53E+04	6.51E+04	6.52E+04	6.50E+04
	IP-SP	Min	3.32E+02	4.02E+02	6.00E+02	8.26E+02	9.00E+02	2.04E+03	2.02E+03	2.22E+03	2.47E+03	2.50E+03	2.60E+03	2.90E+03
		Max	1.31E+05	4.49E+02	6.25E+02	1.01E+03	3.12E+03	2.75E+07	2.23E+03	2.37E+03	2.52E+03	6.42E+03	3.35E+03	2.90E+03
		Mean	5.26E+04	4.31E+02	6.02E+02	9.23E+02	1.11E+03	1.92E+06	2.10E+03	2.25E+03	2.48E+03	3.94E+03	2.94E+03	2.90E+03
		STD	6.56E+04	3.35E+01	5.63E+00	1.31E+02	5.32E+02	7.00E+06	1.16E+02	6.22E+01	1.78E+02	1.92E+03	3.58E+02	2.00E+02
		ET	4.92E-01	4.78E-01	6.91E-01	5.33E-01	5.54E-01	5.12E-01	7.20E-01	7.56E-01	7.13E-01	6.36E-01	8.13E-01	8.40E-01
		NFEs	6.51E+04	6.52E+04	6.52E+04	6.49E+04	6.51E+04	6.53E+04	6.52E+04	6.53E+04	6.50E+04	6.52E+04	6.50E+04	6.52E+04

Table 2 continued from previous page

			F_1	F_2	F_3	F_4	F_5	F_6	F_7	F_8	F_9	F_{10}	F_{11}	F_{12}
GOBL-DE	IP	Min	3.42E+03	4.45E+02	6.00E+02	8.71E+02	9.00E+02	8.41E+03	2.02E+03	2.23E+03	2.48E+03	2.50E+03	2.90E+03	2.93E+03
		Max	1.95E+04	4.49E+02	6.00E+02	9.22E+02	9.00E+02	1.39E+05	2.06E+03	2.24E+03	2.48E+03	5.34E+03	3.00E+03	3.01E+03
		Mean	1.08E+04	4.48E+02	6.00E+02	9.07E+02	9.00E+02	3.47E+04	2.03E+03	2.23E+03	2.48E+03	3.07E+03	2.92E+03	2.94E+03
		STD	1.11E+04	4.83E+01	1.83E-05	1.08E+02	5.11E-11	4.12E+04	3.47E+01	3.27E+01	1.81E+02	1.09E+03	3.22E+02	2.44E+02
		ET	7.29E-01	7.43E-01	8.35E+00	7.69E-01	7.98E-01	7.45E-01	9.42E-01	9.86E-01	1.01E+00	8.82E-01	1.07E+00	1.11E+00
		NFEs	5.01E+04	5.01E+04	5.01E+04	5.01E+04	5.01E+04	5.01E+04	5.01E+04	5.01E+04	5.01E+04	5.01E+04	5.01E+04	5.01E+04
	SP	Min	5.91E+03	4.45E+02	6.00E+02	8.79E+02	9.00E+02	6.73E+03	2.02E+03	2.23E+03	2.48E+03	2.50E+03	2.90E+03	2.93E+03
		Max	1.88E+04	4.49E+02	6.00E+02	9.26E+02	9.00E+02	9.74E+04	2.05E+03	2.24E+03	2.48E+03	4.69E+03	3.00E+03	2.96E+03
		Mean	1.13E+04	4.48E+02	6.00E+02	9.04E+02	9.00E+02	2.77E+04	2.03E+03	2.23E+03	2.48E+03	2.81E+03	2.95E+03	2.94E+03
		STD	1.15E+04	4.81E+01	2.19E-05	1.05E+02	2.42E-11	3.02E+04	3.17E+01	3.27E+01	1.81E+02	7.06E+02	3.50E+02	2.42E+02
		ET	5.91E-01	6.39E-01	9.95E+00	6.75E-01	6.91E-01	6.10E-01	8.93E-01	9.44E-01	9.20E-01	8.17E-01	2.58E+01	1.14E+00
		NFEs	6.52E+04	6.50E+04	6.51E+04	6.50E+04	6.50E+04	6.50E+04	6.51E+04	6.49E+04	6.53E+04	6.48E+04	6.49E+04	6.52E+04
	IP-SP	Min	4.60E+03	4.45E+02	6.00E+02	8.90E+02	9.00E+02	2.84E+03	2.01E+03	2.22E+03	2.48E+03	2.50E+03	2.90E+03	2.93E+03
		Max	1.65E+04	4.49E+02	6.00E+02	9.19E+02	9.00E+02	1.33E+05	2.05E+03	2.24E+03	2.48E+03	4.53E+03	3.00E+03	2.95E+03
		Mean	9.58E+03	4.49E+02	6.00E+02	9.05E+02	9.00E+02	3.50E+04	2.03E+03	2.23E+03	2.48E+03	2.69E+03	2.95E+03	2.94E+03
		STD	9.66E+03	4.88E+01	2.02E-05	1.05E+02	8.29E-02	4.42E+04	3.05E+01	3.22E+01	1.81E+02	5.68E+02	3.50E+02	2.41E+02
		ET	5.90E-01	6.00E-01	8.26E-01	6.94E-01	5.54E+01	6.24E-01	9.13E-01	9.66E-01	9.20E-01	8.10E-01	1.05E+00	1.12E+00
		NFEs	6.51E+04	6.51E+04	6.52E+04	6.52E+04	6.52E+04	6.50E+04	6.50E+04	6.50E+04	6.50E+04	6.50E+04	6.50E+04	6.51E+04

Table 2 continued from previous page

			F_1	F_2	F_3	F_4	F_5	F_6	F_7	F_8	F_9	F_{10}	F_{11}	F_{12}
QOBL-DE	IP	Min	3.96E+03	4.06E+02	6.00E+02	8.92E+02	9.00E+02	4.93E+03	2.02E+03	2.23E+03	2.48E+03	2.50E+03	2.90E+03	2.93E+03
		Max	1.54E+04	4.49E+02	6.00E+02	9.20E+02	9.00E+02	8.30E+04	2.07E+03	2.24E+03	2.48E+03	5.03E+03	3.00E+03	3.02E+03
		Mean	8.90E+03	4.47E+02	6.00E+02	9.06E+02	9.00E+02	2.58E+04	2.03E+03	2.23E+03	2.48E+03	2.66E+03	2.93E+03	2.95E+03
		STD	1.09E+04	4.80E+01	1.84E-05	1.03E+02	2.86E-12	3.00E+04	3.23E+01	3.23E+01	1.81E+02	2.26E+02	3.54E+02	2.39E+02
		ET	7.29E-01	7.43E-01	8.35E+00	7.69E-01	7.98E-01	7.45E-01	9.42E-01	9.86E-01	1.01E+00	8.82E-01	1.07E+00	1.11E+00
		NFEs	6.51E+04	6.49E+04	6.50E+04	6.52E+04	6.51E+04	6.52E+04	6.49E+04	6.49E+04	6.52E+04	6.49E+04	6.51E+04	6.53E+04
	SP	Min	5.42E+03	4.45E+02	6.00E+02	8.78E+02	9.00E+02	4.51E+03	2.02E+03	2.23E+03	2.48E+03	2.50E+03	2.90E+03	2.93E+03
		Max	1.75E+04	4.49E+02	6.00E+02	9.18E+02	9.00E+02	8.59E+04	2.05E+03	2.24E+03	2.48E+03	3.52E+03	3.00E+03	2.95E+03
		Mean	1.06E+04	4.48E+02	6.00E+02	9.03E+02	9.00E+02	2.75E+04	2.03E+03	2.23E+03	2.48E+03	2.53E+03	2.95E+03	2.94E+03
		STD	1.09E+04	4.80E+01	1.84E-05	1.03E+02	2.86E-12	3.00E+04	3.23E+01	3.23E+01	1.81E+02	2.26E+02	3.54E+02	2.39E+02
		ET	7.29E-01	7.43E-01	8.35E+00	7.69E-01	7.98E-01	7.45E-01	9.42E-01	9.86E-01	1.01E+00	8.82E-01	1.07E+00	1.11E+00
		NFEs	6.54E+04	6.52E+04	6.50E+04	6.51E+04	6.49E+04	6.53E+04	6.52E+04	6.48E+04	6.49E+04	6.49E+04	6.51E+04	6.51E+04
	IP-SP	Min	4.86E+03	4.45E+02	6.00E+02	8.88E+02	9.00E+02	3.66E+03	2.02E+03	2.23E+03	2.48E+03	2.50E+03	2.90E+03	2.93E+03
		Max	1.79E+04	4.71E+02	6.00E+02	9.23E+02	9.00E+02	6.38E+04	2.04E+03	2.24E+03	2.48E+03	3.38E+03	3.00E+03	3.00E+03
		Mean	9.40E+03	4.51E+02	6.00E+02	9.06E+02	9.00E+02	1.91E+04	2.03E+03	2.23E+03	2.48E+03	2.55E+03	2.94E+03	2.94E+03
		STD	9.55E+03	5.12E+01	2.25E-05	1.06E+02	1.63E-02	2.13E+04	3.29E+01	3.33E+01	1.81E+02	2.20E+02	3.40E+02	2.44E+02
		ET	7.29E-01	7.43E-01	8.35E+00	7.69E-01	7.98E-01	7.45E-01	9.42E-01	9.86E-01	1.01E+00	8.82E-01	1.07E+00	1.11E+00
		NFEs	6.51E+04	6.49E+04	6.50E+04	6.52E+04	6.51E+04	6.52E+04	6.49E+04	6.49E+04	6.52E+04	6.49E+04	6.51E+04	6.53E+04

Table 2 continued from previous page

			F_1	F_2	F_3	F_4	F_5	F_6	F_7	F_8	F_9	F_{10}	F_{11}	F_{12}
QROBL-DE	IP	Min	4.44E+03	4.49E+02	6.00E+02	8.88E+02	9.00E+02	4.67E+03	2.02E+03	2.22E+03	2.48E+03	2.50E+03	2.90E+03	2.93E+03
		Max	2.36E+04	4.49E+02	6.00E+02	9.19E+02	9.00E+02	5.96E+04	2.05E+03	2.24E+03	2.48E+03	4.52E+03	3.00E+03	2.98E+03
		Mean	1.01E+04	4.49E+02	6.00E+02	9.06E+02	9.00E+02	2.62E+04	2.03E+03	2.23E+03	2.48E+03	2.66E+03	2.94E+03	2.94E+03
		STD	1.08E+04	4.91E+01	1.88E-05	1.06E+02	3.47E-11	2.89E+04	3.25E+01	3.17E+01	1.81E+02	5.10E+02	3.47E+02	2.43E+02
		ET	1.88E+00	1.83E+00	2.22E+00	1.89E+00	2.06E+00	1.76E+00	1.98E+00	9.81E-01	6.27E-01	5.71E-01	7.26E-01	7.10E-01
		NFEs	5.01E+04	5.01E+04	5.01E+04	5.01E+04	5.01E+04	5.01E+04	5.01E+04	5.01E+04	5.01E+04	5.01E+04	5.01E+04	5.01E+04
	SP	Min	2.45E+03	4.45E+02	6.00E+02	8.91E+02	9.00E+02	2.37E+03	2.02E+03	2.23E+03	2.48E+03	2.50E+03	2.60E+03	2.94E+03
		Max	1.01E+04	4.67E+02	6.00E+02	9.16E+02	9.00E+02	9.48E+04	2.05E+03	2.24E+03	2.48E+03	4.96E+03	3.00E+03	2.96E+03
		Mean	5.67E+03	4.50E+02	6.00E+02	9.05E+02	9.00E+02	1.98E+04	2.03E+03	2.23E+03	2.48E+03	2.61E+03	2.92E+03	2.94E+03
		STD	5.62E+03	5.00E+01	1.89E-05	1.05E+02	6.76E-13	2.62E+04	3.15E+01	3.20E+01	1.81E+02	4.89E+02	3.29E+02	2.41E+02
		ET	7.85E-01	7.67E-01	9.89E-01	4.22E+01	8.03E-01	7.42E-01	9.49E-01	1.00E+00	9.78E-01	9.05E-01	1.09E+00	1.13E+00
		NFEs	6.54E+04	6.49E+04	6.50E+04	6.50E+04	6.53E+04	6.52E+04	6.49E+04	6.50E+04	6.51E+04	6.51E+04	6.49E+04	6.50E+04
	IP-SP	Min	2.92E+03	4.06E+02	6.00E+02	8.88E+02	9.00E+02	2.25E+03	2.03E+03	2.23E+03	2.48E+03	2.50E+03	2.60E+03	2.93E+03
		Max	1.03E+04	4.67E+02	6.00E+02	9.20E+02	9.00E+02	1.14E+05	2.04E+03	2.24E+03	2.48E+03	2.70E+03	3.00E+03	2.99E+03
		Mean	5.96E+03	4.48E+02	6.00E+02	9.03E+02	9.00E+02	2.27E+04	2.03E+03	2.23E+03	2.48E+03	2.52E+03	2.93E+03	2.94E+03
		STD	5.90E+03	4.86E+01	2.02E-05	1.04E+02	1.63E-02	3.42E+04	3.19E+01	3.32E+01	1.81E+02	1.27E+02	3.36E+02	2.41E+02
		ET	7.37E-01	7.48E-01	9.06E-01	7.84E-01	7.93E-01	7.51E-01	9.65E-01	1.04E+00	2.96E+01	8.95E-01	1.08E+00	1.17E+00
		NFEs	6.51E+04	6.51E+04	6.51E+04	6.52E+04	6.51E+04	6.51E+04	6.51E+04	6.51E+04	6.50E+04	6.51E+04	6.52E+04	6.51E+04

Table 3: Performance metrics for GA variants across 12 functions ($D = 10$)

			F_1	F_2	F_3	F_4	F_5	F_6	F_7	F_8	F_9	F_{10}	F_{11}	F_{12}
GA		Min	8.25E+02	4.00E+02	6.00E+02	8.05E+02	9.00E+02	1.84E+03	2.00E+03	2.21E+03	2.53E+03	2.50E+03	2.60E+03	2.87E+03
		Max	1.52E+04	4.78E+02	6.00E+02	8.28E+02	1.02E+03	8.03E+03	2.02E+03	2.34E+03	2.55E+03	2.62E+03	2.92E+03	2.89E+03
		Mean	5.11E+03	4.16E+02	6.00E+02	8.17E+02	9.16E+02	3.29E+03	2.02E+03	2.22E+03	2.54E+03	2.52E+03	2.70E+03	2.87E+03
		STD	5.91E+03	3.05E+01	3.62E-02	1.78E+01	2.88E+01	2.20E+03	1.98E+01	3.24E+01	2.37E+02	1.23E+02	1.39E+02	1.72E+02
		ET	1.33E+00	1.37E+00	1.40E+00	1.33E+00	3.74E+01	1.34E+00	1.46E+00	1.46E+00	1.45E+00	2.28E+01	1.48E+00	1.50E+00
		NFEs	5.10E+04	5.10E+04	5.10E+04	5.10E+04	5.10E+04	5.10E+04	5.10E+04	5.10E+04	5.10E+04	5.10E+04	5.10E+04	5.10E+04
BOBL-GA	IP	Min	1.06E+03	4.00E+02	6.00E+02	8.07E+02	9.00E+02	1.83E+03	2.00E+03	2.21E+03	2.53E+03	2.40E+03	2.60E+03	2.86E+03
		Max	1.68E+04	4.71E+02	6.00E+02	8.36E+02	2.11E+03	7.95E+03	2.02E+03	2.22E+03	2.55E+03	2.76E+03	3.00E+03	2.89E+03
		Mean	6.69E+03	4.11E+02	6.00E+02	8.18E+02	9.71E+02	3.15E+03	2.02E+03	2.22E+03	2.54E+03	2.55E+03	2.69E+03	2.87E+03
		STD	7.56E+03	1.93E+01	2.37E-02	1.98E+01	2.30E+02	1.99E+03	1.91E+01	2.05E+01	2.37E+02	1.63E+02	1.35E+02	1.70E+02
		ET	1.33E+00	1.32E+00	1.39E+00	1.33E+00	1.79E+01	1.32E+00	1.49E+00	1.49E+00	1.46E+00	1.64E+01	1.49E+00	1.51E+00
		NFEs	5.11E+04	5.11E+04	5.11E+04	5.11E+04	5.11E+04	5.11E+04	5.11E+04	5.11E+04	5.11E+04	5.11E+04	5.11E+04	5.11E+04
	SP	Min	1.43E+03	4.00E+02	6.00E+02	8.06E+02	9.00E+02	1.81E+03	2.00E+03	2.22E+03	2.53E+03	2.50E+03	2.60E+03	2.86E+03
		Max	1.48E+04	4.75E+02	6.00E+02	8.43E+02	9.78E+02	7.47E+03	2.02E+03	2.22E+03	2.55E+03	2.64E+03	3.20E+03	2.89E+03
		Mean	5.48E+03	4.08E+02	6.00E+02	8.17E+02	9.17E+02	3.13E+03	2.02E+03	2.22E+03	2.53E+03	2.56E+03	2.73E+03	2.87E+03
		STD	6.15E+03	1.94E+01	2.20E-02	1.88E+01	2.65E+01	2.10E+03	1.77E+01	2.08E+01	2.35E+02	1.71E+02	1.87E+02	1.72E+02
		ET	1.40E+00	1.37E+00	1.50E+00	1.42E+00	3.31E+00	3.50E+00	4.25E+00	3.86E+00	4.09E+00	4.71E+00	4.44E+00	5.69E+00
		NFEs	6.61E+04	6.61E+04	6.61E+04	6.60E+04	6.61E+04	6.59E+04	6.63E+04	6.64E+04	6.57E+04	6.62E+04	6.59E+04	6.59E+04
	IP-SP	Min	1.87E+03	4.00E+02	6.00E+02	8.07E+02	9.01E+02	1.83E+03	2.00E+03	2.20E+03	2.53E+03	2.50E+03	2.60E+03	2.86E+03
		Max	1.70E+04	4.68E+02	6.00E+02	8.35E+02	1.14E+03	6.44E+03	2.02E+03	2.22E+03	2.55E+03	2.63E+03	3.00E+03	2.89E+03
		Mean	7.47E+03	4.09E+02	6.00E+02	8.19E+02	9.35E+02	3.08E+03	2.02E+03	2.22E+03	2.54E+03	2.57E+03	2.76E+03	2.87E+03
		STD	8.41E+03	1.44E+01	2.34E-02	2.00E+01	6.94E+01	1.74E+03	1.91E+01	1.98E+01	2.36E+02	1.76E+02	2.00E+02	1.70E+02
		ET	1.39E+00	1.36E+00	1.48E+00	4.30E+01	1.43E+00	1.39E+00	1.55E+00	7.81E+00	4.49E+00	4.03E+00	4.39E+00	4.48E+00
		NFEs	6.60E+04	6.60E+04	6.59E+04	6.60E+04	6.61E+04	6.60E+04	6.62E+04	6.62E+04	6.61E+04	6.61E+04	6.61E+04	6.60E+04

Table 3 continued from previous page

			F_1	F_2	F_3	F_4	F_5	F_6	F_7	F_8	F_9	F_{10}	F_{11}	F_{12}
COOBL-GA	IP	Min	2.47E+03	4.00E+02	6.00E+02	8.07E+02	9.00E+02	1.84E+03	2.00E+03	2.20E+03	2.53E+03	2.50E+03	2.60E+03	2.86E+03
		Max	2.13E+04	4.90E+02	6.00E+02	8.59E+02	1.28E+03	7.95E+03	2.02E+03	2.34E+03	2.56E+03	2.67E+03	3.20E+03	2.94E+03
		Mean	8.05E+03	4.22E+02	6.00E+02	8.22E+02	9.63E+02	4.17E+03	2.02E+03	2.23E+03	2.54E+03	2.55E+03	2.78E+03	2.87E+03
		STD	8.92E+03	3.70E+01	1.36E-02	2.48E+01	1.28E+02	3.01E+03	1.83E+01	4.11E+01	2.38E+02	1.60E+02	2.52E+02	1.73E+02
		ET	1.69E+00	1.61E+00	1.71E+00	2.12E+02	1.65E+00	1.60E+00	1.76E+00	1.99E+01	1.74E+00	1.72E+00	1.87E+00	7.53E+01
		NFEs	5.11E+04	5.11E+04	5.11E+04	5.11E+04	5.11E+04	5.11E+04	5.11E+04	5.11E+04	5.11E+04	5.11E+04	5.11E+04	5.11E+04
	SP	Min	3.99E+02	4.00E+02	6.00E+02	8.05E+02	9.01E+02	1.85E+03	2.00E+03	2.21E+03	2.50E+03	2.50E+03	2.60E+03	2.86E+03
		Max	1.42E+04	4.91E+02	6.00E+02	8.51E+02	1.25E+03	7.69E+03	2.02E+03	2.34E+03	2.64E+03	2.64E+03	3.20E+03	2.90E+03
		Mean	4.84E+03	4.19E+02	6.00E+02	8.19E+02	9.55E+02	3.46E+03	2.02E+03	2.23E+03	2.54E+03	2.54E+03	2.69E+03	2.87E+03
		STD	5.82E+03	3.40E+01	4.17E-02	2.12E+01	9.62E+01	2.20E+03	1.83E+01	4.80E+01	2.37E+02	1.54E+02	1.78E+02	1.73E+02
		ET	1.08E+00	1.08E+00	1.16E+00	1.10E+00	1.11E+00	1.08E+00	1.24E+00	1.25E+00	1.22E+00	2.62E+01	1.29E+00	1.29E+00
		NFEs	6.61E+04	6.60E+04	6.59E+04	6.60E+04	6.61E+04	6.59E+04	6.59E+04	6.59E+04	6.61E+04	6.60E+04	6.61E+04	6.59E+04
	IP-SP	Min	5.49E+02	4.00E+02	6.00E+02	8.04E+02	9.00E+02	1.82E+03	2.00E+03	2.22E+03	2.51E+03	2.50E+03	2.60E+03	2.86E+03
		Max	1.51E+04	4.85E+02	6.00E+02	8.49E+02	1.53E+03	8.06E+03	2.02E+03	2.34E+03	2.62E+03	2.78E+03	3.20E+03	2.94E+03
		Mean	5.83E+03	4.20E+02	6.00E+02	8.24E+02	1.04E+03	3.99E+03	2.02E+03	2.22E+03	2.54E+03	2.56E+03	2.75E+03	2.88E+03
		STD	6.73E+03	3.30E+01	3.12E-02	2.71E+01	2.32E+02	3.03E+03	1.97E+01	3.25E+01	2.41E+02	1.77E+02	2.41E+02	1.77E+02
		ET	1.39E+00	1.36E+00	1.49E+00	1.40E+00	1.45E+00	1.68E+00	1.87E+00	1.94E+00	1.90E+00	2.68E+00	2.90E+00	2.71E+00
		NFEs	6.62E+04	6.60E+04	6.60E+04	6.61E+04	6.60E+04	6.61E+04	6.61E+04	6.60E+04	6.61E+04	6.62E+04	6.61E+04	6.61E+04

Table 3 continued from previous page

			F_1	F_2	F_3	F_4	F_5	F_6	F_7	F_8	F_9	F_{10}	F_{11}	F_{12}
GOBL-GA	IP	Min	6.53E+02	4.00E+02	6.00E+02	8.06E+02	9.00E+02	1.81E+03	2.00E+03	2.20E+03	2.53E+03	2.50E+03	2.60E+03	2.87E+03
		Max	2.29E+04	4.79E+02	6.00E+02	8.54E+02	1.04E+03	8.03E+03	2.02E+03	2.22E+03	2.68E+03	2.65E+03	3.20E+03	2.90E+03
		Mean	5.96E+03	4.15E+02	6.00E+02	8.21E+02	9.21E+02	3.62E+03	2.02E+03	2.22E+03	2.54E+03	2.55E+03	2.71E+03	2.88E+03
		STD	7.04E+03	3.00E+01	4.58E-02	2.49E+01	3.65E+01	2.50E+03	1.80E+01	2.01E+01	2.43E+02	1.65E+02	1.87E+02	1.76E+02
		ET	1.31E+00	1.34E+00	1.38E+00	1.31E+00	2.73E+00	2.92E+00	3.56E+00	3.90E+00	3.45E+00	2.90E+00	3.85E+00	3.72E+00
		NFEs	5.11E+04	5.11E+04	5.11E+04	5.11E+04	5.11E+04	5.11E+04	5.11E+04	5.11E+04	5.11E+04	5.11E+04	5.11E+04	5.11E+04
	SP	Min	1.34E+03	4.00E+02	6.00E+02	8.03E+02	9.00E+02	1.81E+03	2.00E+03	2.21E+03	2.53E+03	2.50E+03	2.60E+03	2.86E+03
		Max	1.57E+04	4.81E+02	6.00E+02	8.52E+02	9.60E+02	6.79E+03	2.02E+03	2.22E+03	2.55E+03	2.63E+03	3.00E+03	2.94E+03
		Mean	6.01E+03	4.11E+02	6.00E+02	8.15E+02	9.13E+02	3.53E+03	2.02E+03	2.22E+03	2.54E+03	2.54E+03	2.66E+03	2.87E+03
		STD	6.71E+03	2.38E+01	2.62E-02	1.83E+01	1.88E+01	2.33E+03	1.93E+01	1.92E+01	2.35E+02	1.47E+02	1.14E+02	1.75E+02
		ET	1.38E+00	1.37E+00	1.54E+00	1.41E+00	1.41E+00	1.38E+00	1.55E+00	1.58E+00	1.51E+00	1.49E+00	1.63E+00	1.65E+00
		NFEs	6.59E+04	6.62E+04	6.61E+04	6.59E+04	6.61E+04	6.62E+04	6.59E+04	6.62E+04	6.61E+04	6.61E+04	6.63E+04	6.60E+04
	IP-SP	Min	7.73E+02	4.00E+02	6.00E+02	8.05E+02	9.00E+02	1.87E+03	2.00E+03	2.22E+03	2.53E+03	2.50E+03	2.60E+03	2.87E+03
		Max	1.15E+04	4.91E+02	6.00E+02	8.34E+02	9.91E+02	8.00E+03	2.02E+03	2.34E+03	2.55E+03	2.62E+03	3.18E+03	2.93E+03
		Mean	4.56E+03	4.16E+02	6.00E+02	8.18E+02	9.16E+02	4.11E+03	2.02E+03	2.22E+03	2.54E+03	2.53E+03	2.69E+03	2.88E+03
		STD	4.95E+03	3.17E+01	2.01E-02	1.99E+01	2.51E+01	3.04E+03	1.84E+01	3.27E+01	2.36E+02	1.37E+02	1.61E+02	1.77E+02
		ET	1.39E+00	1.36E+00	1.49E+00	1.40E+00	1.45E+00	1.68E+00	1.87E+00	1.94E+00	1.90E+00	2.68E+00	2.90E+00	2.71E+00
		NFEs	6.60E+04	6.60E+04	6.61E+04	6.62E+04	6.62E+04	6.62E+04	6.62E+04	6.63E+04	6.64E+04	6.62E+04	6.59E+04	6.62E+04

Table 3 continued from previous page

			F_1	F_2	F_3	F_4	F_5	F_6	F_7	F_8	F_9	F_{10}	F_{11}	F_{12}
QOBL-GA	IP	Min	7.58E+02	4.00E+02	6.00E+02	8.04E+02	9.00E+02	1.82E+03	2.00E+03	2.20E+03	2.53E+03	2.50E+03	2.60E+03	2.86E+03
		Max	1.11E+04	4.75E+02	6.00E+02	8.33E+02	1.01E+03	7.44E+03	2.02E+03	2.22E+03	2.55E+03	2.63E+03	2.90E+03	2.89E+03
		Mean	3.48E+03	4.13E+02	6.00E+02	8.20E+02	9.17E+02	3.39E+03	2.02E+03	2.22E+03	2.54E+03	2.54E+03	2.67E+03	2.87E+03
		STD	3.96E+03	2.75E+01	2.05E-02	2.12E+01	3.16E+01	2.41E+03	1.81E+01	2.03E+01	2.36E+02	1.49E+02	1.20E+02	1.73E+02
		ET	1.64E+00	1.56E+00	1.66E+00	1.58E+00	2.62E+01	1.57E+00	1.70E+00	1.74E+00	1.68E+00	1.66E+00	1.01E+01	1.80E+00
		NFEs	5.11E+04	5.11E+04	5.11E+04	5.11E+04	5.11E+04	5.11E+04	5.11E+04	5.11E+04	5.11E+04	5.11E+04	5.11E+04	5.11E+04
	SP	Min	6.34E+02	4.00E+02	6.00E+02	8.04E+02	9.00E+02	1.83E+03	2.00E+03	2.20E+03	2.53E+03	2.50E+03	2.60E+03	2.86E+03
		Max	8.25E+03	4.77E+02	6.00E+02	8.40E+02	1.20E+03	6.61E+03	2.02E+03	2.22E+03	2.54E+03	2.63E+03	3.18E+03	2.90E+03
		Mean	2.93E+03	4.11E+02	6.00E+02	8.17E+02	9.37E+02	2.82E+03	2.02E+03	2.22E+03	2.53E+03	2.54E+03	2.69E+03	2.87E+03
		STD	3.25E+03	2.34E+01	3.02E-02	1.93E+01	8.00E+01	1.62E+03	1.76E+01	2.02E+01	2.34E+02	1.51E+02	1.60E+02	1.71E+02
		ET	2.06E+00	1.73E+00	1.71E+00	8.21E+01	1.60E+00	1.55E+00	1.88E+00	1.00E+01	1.95E+00	1.66E+00	8.07E+00	1.79E+00
		NFEs	6.62E+04	6.61E+04	6.61E+04	6.61E+04	6.61E+04	6.62E+04	6.61E+04	6.59E+04	6.60E+04	6.62E+04	6.60E+04	6.61E+04
	IP-SP	Min	8.05E+02	4.00E+02	6.00E+02	8.06E+02	9.00E+02	1.81E+03	2.00E+03	2.20E+03	2.53E+03	2.50E+03	2.60E+03	2.87E+03
		Max	5.98E+03	4.69E+02	6.00E+02	8.41E+02	9.71E+02	6.41E+03	2.02E+03	2.34E+03	2.55E+03	2.64E+03	2.90E+03	2.89E+03
		Mean	2.39E+03	4.09E+02	6.00E+02	8.20E+02	9.17E+02	3.15E+03	2.02E+03	2.22E+03	2.54E+03	2.56E+03	2.65E+03	2.87E+03
		STD	2.42E+03	1.86E+01	2.01E-02	2.14E+01	2.50E+01	2.01E+03	1.85E+01	3.21E+01	2.36E+02	1.66E+02	1.03E+02	1.72E+02
		ET	1.59E+00	1.58E+00	1.69E+00	1.77E+00	2.66E+00	1.60E+00	1.75E+00	1.96E+00	5.90E+00	1.68E+00	1.79E+00	1.93E+00
		NFEs	6.61E+04	6.61E+04	6.61E+04	6.60E+04	6.61E+04	6.60E+04	6.62E+04	6.59E+04	6.61E+04	6.62E+04	6.61E+04	6.60E+04

Table 3 continued from previous page

			F_1	F_2	F_3	F_4	F_5	F_6	F_7	F_8	F_9	F_{10}	F_{11}	F_{12}
QROBL-GA	IP	Min	7.76E+02	4.00E+02	6.00E+02	8.06E+02	9.01E+02	1.83E+03	2.00E+03	2.21E+03	2.53E+03	2.50E+03	2.60E+03	2.87E+03
		Max	1.24E+04	4.71E+02	6.00E+02	8.34E+02	9.44E+02	7.93E+03	2.02E+03	2.22E+03	2.55E+03	2.63E+03	3.00E+03	2.91E+03
		Mean	3.81E+03	4.20E+02	6.00E+02	8.18E+02	9.12E+02	2.90E+03	2.02E+03	2.22E+03	2.54E+03	2.55E+03	2.71E+03	2.87E+03
		STD	4.44E+03	3.64E+01	1.91E-02	1.88E+01	1.72E+01	1.83E+03	1.80E+01	2.04E+01	2.36E+02	1.58E+02	1.70E+02	1.74E+02
		ET	1.33E+00	1.32E+00	1.39E+00	1.34E+00	6.99E+00	1.30E+00	1.43E+00	1.45E+00	1.40E+00	3.58E+00	3.50E+00	1.54E+00
		NFEs	5.11E+04	5.11E+04	5.11E+04	5.11E+04	5.11E+04	5.11E+04	5.11E+04	5.11E+04	5.11E+04	5.11E+04	5.11E+04	5.11E+04
	SP	Min	5.12E+02	4.00E+02	6.00E+02	8.09E+02	9.00E+02	1.81E+03	2.00E+03	2.21E+03	2.53E+03	2.50E+03	2.60E+03	2.86E+03
		Max	3.39E+03	4.72E+02	6.00E+02	8.33E+02	9.93E+02	5.46E+03	2.02E+03	2.22E+03	2.55E+03	2.62E+03	3.00E+03	2.92E+03
		Mean	1.56E+03	4.14E+02	6.00E+02	8.19E+02	9.14E+02	3.14E+03	2.02E+03	2.22E+03	2.54E+03	2.54E+03	2.68E+03	2.87E+03
		STD	1.44E+03	2.86E+01	2.73E-02	2.01E+01	2.73E+01	1.82E+03	1.87E+01	2.06E+01	2.37E+02	1.54E+02	1.50E+02	1.74E+02
		ET	3.83E+00	4.14E+00	5.29E+00	4.12E+00	4.16E+00	4.59E+00	4.53E+00	3.69E+00	1.69E+00	1.66E+00	1.84E+00	3.20E+01
		NFEs	6.61E+04	6.61E+04	6.60E+04	6.62E+04	6.61E+04	6.61E+04	6.60E+04	6.62E+04	6.61E+04	6.62E+04	6.60E+04	6.61E+04
	IP-SP	Min	6.36E+02	4.00E+02	6.00E+02	8.07E+02	9.00E+02	1.81E+03	2.00E+03	2.20E+03	2.53E+03	2.50E+03	2.60E+03	2.87E+03
		Max	3.67E+03	4.73E+02	6.00E+02	8.30E+02	9.99E+02	7.29E+03	2.02E+03	2.22E+03	2.54E+03	2.62E+03	2.91E+03	2.90E+03
		Mean	1.78E+03	4.15E+02	6.00E+02	8.19E+02	9.21E+02	3.36E+03	2.02E+03	2.22E+03	2.54E+03	2.54E+03	2.68E+03	2.87E+03
		STD	1.66E+03	2.91E+01	2.33E-02	2.01E+01	3.11E+01	2.27E+03	1.77E+01	2.06E+01	2.36E+02	1.46E+02	1.38E+02	1.74E+02
		ET	2.45E+00	1.78E+00	1.68E+00	1.61E+00	1.67E+00	1.58E+00	1.74E+00	1.81E+00	2.40E+00	1.69E+00	1.82E+00	1.88E+00
		NFEs	6.62E+04	6.59E+04	6.59E+04	6.60E+04	6.58E+04	6.62E+04	6.63E+04	6.63E+04	6.62E+04	6.60E+04	6.63E+04	6.60E+04

Table 4: Performance metrics for GA variants across 12 functions ($D = 20$)

			F_1	F_2	F_3	F_4	F_5	F_6	F_7	F_8	F_9	F_{10}	F_{11}	F_{12}
GA		Min	4.54E+03	4.01E+02	6.00E+02	8.22E+02	9.16E+02	1.96E+03	2.03E+03	2.22E+03	2.48E+03	2.50E+03	2.90E+03	2.95E+03
		Max	2.36E+04	5.15E+02	6.03E+02	8.83E+02	1.29E+03	1.94E+04	2.20E+03	2.46E+03	2.50E+03	3.02E+03	3.00E+03	3.00E+03
		Mean	1.19E+04	4.56E+02	6.01E+02	8.47E+02	1.05E+03	7.68E+03	2.07E+03	2.27E+03	2.49E+03	2.61E+03	2.93E+03	2.97E+03
		STD	1.26E+04	5.91E+01	9.18E-01	4.97E+01	1.81E+02	7.64E+03	7.79E+01	9.29E+01	1.87E+02	2.42E+02	3.31E+02	2.69E+02
		ET	1.39E+00	2.97E+00	6.53E+00	4.91E+00	4.45E+00	3.37E+00	4.64E+00	3.47E+00	3.52E+00	2.17E+01	1.77E+00	1.79E+00
		NFEs	5.11E+04	5.11E+04	5.11E+04	5.11E+04	5.11E+04	5.11E+04	5.11E+04	5.11E+04	5.11E+04	5.11E+04	5.11E+04	5.11E+04
BOBL-GA	IP	Min	3.46E+03	4.28E+02	6.00E+02	8.23E+02	9.19E+02	1.91E+03	2.03E+03	2.22E+03	2.48E+03	2.44E+03	2.60E+03	2.94E+03
		Max	3.90E+04	4.76E+02	6.02E+02	8.97E+02	3.16E+03	1.95E+04	2.19E+03	2.34E+03	2.50E+03	3.12E+03	3.00E+03	3.05E+03
		Mean	1.36E+04	4.59E+02	6.00E+02	8.55E+02	1.34E+03	7.12E+03	2.07E+03	2.23E+03	2.49E+03	2.69E+03	2.90E+03	2.98E+03
		STD	1.52E+04	6.11E+01	6.70E-01	5.84E+01	6.83E+02	7.21E+03	8.61E+01	4.65E+01	1.86E+02	3.38E+02	3.05E+02	2.84E+02
		ET	1.39E+00	2.97E+00	6.53E+00	4.91E+00	4.45E+00	3.37E+00	4.64E+00	3.47E+00	3.52E+00	2.17E+01	1.77E+00	1.79E+00
		NFEs	5.11E+04	5.11E+04	5.11E+04	5.11E+04	5.11E+04	5.11E+04	5.11E+04	5.11E+04	5.11E+04	5.11E+04	5.11E+04	5.11E+04
	SP	Min	3.51E+03	4.05E+02	6.00E+02	8.17E+02	9.05E+02	2.13E+03	2.03E+03	2.22E+03	2.48E+03	2.50E+03	2.90E+03	2.95E+03
		Max	2.79E+04	4.75E+02	6.03E+02	8.75E+02	2.32E+03	1.95E+04	2.19E+03	2.36E+03	2.50E+03	3.25E+03	3.00E+03	3.04E+03
		Mean	1.21E+04	4.57E+02	6.00E+02	8.48E+02	1.15E+03	7.15E+03	2.07E+03	2.24E+03	2.49E+03	2.67E+03	2.93E+03	2.97E+03
		STD	1.31E+04	5.92E+01	7.64E-01	4.95E+01	4.14E+02	7.07E+03	7.83E+01	5.88E+01	1.88E+02	3.23E+02	3.28E+02	2.70E+02
		ET	3.72E+00	3.68E+00	3.98E+00	1.26E+01	3.91E+00	3.64E+00	4.23E+00	5.38E+00	4.25E+00	4.35E+00	4.75E+00	4.70E+00
		NFEs	6.60E+04	6.63E+04	6.60E+04	6.62E+04	6.58E+04	6.60E+04	6.61E+04	6.60E+04	6.60E+04	6.59E+04	6.62E+04	6.62E+04
	IP-SP	Min	4.58E+03	4.45E+02	6.00E+02	8.29E+02	9.12E+02	2.00E+03	2.02E+03	2.22E+03	2.48E+03	2.41E+03	2.90E+03	2.93E+03
		Max	3.25E+04	5.21E+02	6.03E+02	8.94E+02	1.83E+03	2.20E+04	2.15E+03	2.36E+03	2.49E+03	2.92E+03	3.00E+03	3.04E+03
		Mean	1.42E+04	4.60E+02	6.01E+02	8.47E+02	1.13E+03	6.81E+03	2.06E+03	2.24E+03	2.49E+03	2.64E+03	2.93E+03	2.98E+03
		STD	1.63E+04	6.19E+01	1.03E+00	4.99E+01	3.15E+02	7.12E+03	6.61E+01	5.98E+01	1.87E+02	2.92E+02	3.29E+02	2.77E+02
		ET	4.57E+00	2.24E+00	1.73E+00	1.55E+00	1.59E+00	1.44E+01	1.81E+00	1.84E+00	4.06E+01	1.79E+00	1.97E+00	2.09E+00
		NFEs	6.63E+04	6.61E+04	6.62E+04	6.59E+04	6.62E+04	6.60E+04	6.60E+04	6.59E+04	6.60E+04	6.60E+04	6.61E+04	6.61E+04

Table 4 continued from previous page

			F_1	F_2	F_3	F_4	F_5	F_6	F_7	F_8	F_9	F_{10}	F_{11}	F_{12}
COOBL-GA	IP	Min	6.05E+03	4.45E+02	6.00E+02	8.25E+02	9.27E+02	2.07E+03	2.03E+03	2.22E+03	2.48E+03	2.41E+03	2.60E+03	2.94E+03
		Max	3.18E+04	4.89E+02	6.01E+02	9.08E+02	3.35E+03	2.10E+04	2.17E+03	2.40E+03	2.50E+03	3.04E+03	3.00E+03	3.06E+03
		Mean	1.85E+04	4.62E+02	6.00E+02	8.50E+02	1.25E+03	8.71E+03	2.08E+03	2.26E+03	2.49E+03	2.63E+03	2.92E+03	2.98E+03
		STD	1.98E+04	6.31E+01	4.88E-01	5.28E+01	5.54E+02	9.03E+03	8.92E+01	7.81E+01	1.88E+02	2.86E+02	3.26E+02	2.79E+02
		ET	1.82E+00	1.78E+00	9.95E+00	2.01E+00	2.08E+00	2.00E+00	2.29E+00	1.70E+00	1.30E+00	1.23E+00	1.37E+00	1.44E+00
		NFEs	5.10E+04	5.10E+04	5.10E+04	5.10E+04	5.10E+04	5.10E+04	5.10E+04	5.10E+04	5.10E+04	5.10E+04	5.10E+04	5.10E+04
	SP	Min	7.99E+02	4.45E+02	6.00E+02	8.21E+02	9.59E+02	1.91E+03	2.02E+03	2.22E+03	2.48E+03	2.50E+03	2.60E+03	2.90E+03
		Max	1.45E+04	5.36E+02	6.02E+02	8.82E+02	2.11E+03	3.30E+04	2.18E+03	2.46E+03	2.51E+03	3.23E+03	3.00E+03	3.03E+03
		Mean	6.07E+03	4.61E+02	6.00E+02	8.49E+02	1.39E+03	9.94E+03	2.07E+03	2.28E+03	2.49E+03	2.67E+03	2.88E+03	2.93E+03
		STD	6.78E+03	6.38E+01	6.11E-01	5.21E+01	6.03E+02	1.20E+04	7.49E+01	1.04E+02	1.88E+02	3.29E+02	2.99E+02	2.30E+02
		ET	1.15E+00	1.15E+00	1.34E+00	1.26E+00	1.22E+00	1.13E+00	1.76E+01	1.36E+00	1.33E+00	1.25E+00	1.43E+00	1.47E+00
		NFEs	6.61E+04	6.63E+04	6.62E+04	6.60E+04	6.60E+04	6.60E+04	6.59E+04	6.58E+04	6.61E+04	6.62E+04	6.62E+04	6.61E+04
	IP-SP	Min	1.76E+03	4.26E+02	6.00E+02	8.28E+02	9.43E+02	2.02E+03	2.02E+03	2.22E+03	2.48E+03	2.50E+03	2.61E+03	2.90E+03
		Max	1.80E+04	4.75E+02	6.05E+02	9.06E+02	5.77E+03	3.71E+06	2.24E+03	2.37E+03	2.51E+03	3.24E+03	3.00E+03	3.05E+03
		Mean	8.45E+03	4.59E+02	6.01E+02	8.64E+02	1.92E+03	1.34E+05	2.08E+03	2.26E+03	2.49E+03	2.76E+03	2.91E+03	2.94E+03
		STD	9.16E+03	6.02E+01	1.12E+00	6.65E+01	1.43E+03	6.77E+05	9.64E+01	8.31E+01	1.89E+02	4.05E+02	3.22E+02	2.45E+02
		ET	1.08E+00	1.07E+00	1.27E+00	1.15E+00	1.15E+00	1.11E+00	1.33E+00	5.29E+00	1.34E+00	1.26E+00	1.46E+00	1.47E+00
		NFEs	6.63E+04	6.59E+04	6.60E+04	6.61E+04	6.61E+04	6.59E+04	6.59E+04	6.59E+04	6.62E+04	6.61E+04	6.62E+04	6.61E+04

Table 4 continued from previous page

			F_1	F_2	F_3	F_4	F_5	F_6	F_7	F_8	F_9	F_{10}	F_{11}	F_{12}
GOBL-GA	IP	Min	3.43E+03	4.49E+02	6.00E+02	8.24E+02	9.15E+02	1.88E+03	2.02E+03	2.22E+03	2.48E+03	2.41E+03	2.90E+03	2.95E+03
		Max	3.04E+04	5.07E+02	6.01E+02	8.85E+02	1.87E+03	1.90E+04	2.18E+03	2.36E+03	2.49E+03	3.00E+03	3.00E+03	3.02E+03
		Mean	1.63E+04	4.64E+02	6.01E+02	8.49E+02	1.15E+03	6.83E+03	2.07E+03	2.26E+03	2.49E+03	2.62E+03	2.93E+03	2.97E+03
		STD	1.74E+04	6.52E+01	6.06E-01	5.26E+01	3.75E+02	6.88E+03	8.41E+01	7.76E+01	1.86E+02	2.65E+02	3.31E+02	2.71E+02
		ET	3.06E+00	3.38E+00	4.03E+00	3.46E+00	3.73E+00	3.50E+00	4.20E+00	4.17E+00	4.06E+00	4.30E+00	4.51E+00	1.90E+00
		NFEs	5.11E+04	5.11E+04	5.11E+04	5.11E+04	5.11E+04	5.11E+04	5.11E+04	5.11E+04	5.11E+04	5.11E+04	5.11E+04	5.11E+04
	SP	Min	4.49E+03	4.41E+02	6.00E+02	8.14E+02	9.39E+02	1.95E+03	2.02E+03	2.22E+03	2.48E+03	2.41E+03	2.90E+03	2.94E+03
		Max	3.37E+04	4.97E+02	6.02E+02	8.86E+02	1.48E+03	1.61E+04	2.16E+03	2.36E+03	2.50E+03	3.01E+03	3.00E+03	3.05E+03
		Mean	1.33E+04	4.56E+02	6.00E+02	8.49E+02	1.07E+03	6.35E+03	2.07E+03	2.25E+03	2.49E+03	2.62E+03	2.92E+03	2.98E+03
		STD	1.46E+04	5.73E+01	4.95E-01	5.24E+01	2.12E+02	6.05E+03	7.75E+01	7.01E+01	1.88E+02	2.78E+02	3.17E+02	2.77E+02
		ET	1.47E+00	1.46E+00	1.73E+00	1.53E+00	1.54E+00	2.99E+00	1.80E+00	1.84E+00	1.80E+00	1.71E+00	1.93E+00	2.00E+00
		NFEs	6.60E+04	6.61E+04	6.62E+04	6.60E+04	6.59E+04	6.62E+04	6.60E+04	6.57E+04	6.62E+04	6.62E+04	6.59E+04	6.65E+04
	IP-SP	Min	3.11E+03	4.29E+02	6.00E+02	8.16E+02	9.25E+02	2.00E+03	2.02E+03	2.22E+03	2.48E+03	2.50E+03	2.60E+03	2.95E+03
		Max	3.01E+04	5.21E+02	6.02E+02	8.92E+02	1.55E+03	2.01E+04	2.16E+03	2.46E+03	2.49E+03	3.05E+03	3.00E+03	3.03E+03
		Mean	1.37E+04	4.60E+02	6.00E+02	8.52E+02	1.13E+03	6.61E+03	2.06E+03	2.26E+03	2.49E+03	2.63E+03	2.91E+03	2.97E+03
		STD	1.54E+04	6.24E+01	4.41E-01	5.50E+01	2.86E+02	6.80E+03	7.03E+01	8.53E+01	1.86E+02	2.75E+02	3.22E+02	2.74E+02
		ET	2.44E+00	2.56E+00	3.74E+00	2.42E+00	2.34E+00	2.44E+00	3.15E+00	3.08E+00	3.11E+00	2.79E+00	2.68E+00	2.61E+00
		NFEs	6.62E+04	6.59E+04	6.62E+04	6.61E+04	6.61E+04	6.60E+04	6.63E+04	6.62E+04	6.61E+04	6.61E+04	6.63E+04	6.60E+04

Table 4 continued from previous page

			F_1	F_2	F_3	F_4	F_5	F_6	F_7	F_8	F_9	F_{10}	F_{11}	F_{12}
QOBL-GA	IP	Min	2.45E+03	4.28E+02	6.00E+02	8.26E+02	9.51E+02	1.99E+03	2.02E+03	2.22E+03	2.48E+03	2.41E+03	2.60E+03	2.95E+03
		Max	3.35E+04	4.74E+02	6.01E+02	8.77E+02	2.40E+03	1.72E+04	2.14E+03	2.34E+03	2.50E+03	3.25E+03	3.00E+03	3.05E+03
		Mean	1.15E+04	4.58E+02	6.00E+02	8.52E+02	1.24E+03	6.41E+03	2.05E+03	2.24E+03	2.49E+03	2.60E+03	2.92E+03	2.98E+03
		STD	1.31E+04	5.92E+01	3.89E-01	5.41E+01	4.61E+02	6.08E+03	5.89E+01	6.24E+01	1.87E+02	2.52E+02	3.25E+02	2.79E+02
		ET	1.67E+00	1.65E+00	1.88E+00	1.70E+00	3.18E+00	1.67E+00	1.97E+00	1.20E+01	2.07E+00	1.98E+00	5.05E+00	2.26E+00
		NFEs	5.11E+04	5.11E+04	5.11E+04	5.11E+04	5.11E+04	5.11E+04	5.11E+04	5.11E+04	5.11E+04	5.11E+04	5.11E+04	5.11E+04
	SP	Min	3.46E+03	4.45E+02	6.00E+02	8.18E+02	9.26E+02	1.95E+03	2.03E+03	2.22E+03	2.48E+03	2.50E+03	2.90E+03	2.94E+03
		Max	2.33E+04	4.76E+02	6.01E+02	8.89E+02	1.78E+03	1.78E+04	2.18E+03	2.36E+03	2.50E+03	2.89E+03	3.00E+03	3.02E+03
		Mean	1.26E+04	4.56E+02	6.00E+02	8.50E+02	1.10E+03	5.84E+03	2.07E+03	2.24E+03	2.49E+03	2.61E+03	2.92E+03	2.97E+03
		STD	1.34E+04	5.74E+01	4.90E-01	5.37E+01	2.98E+02	5.77E+03	7.28E+01	6.02E+01	1.87E+02	2.49E+02	3.27E+02	2.72E+02
		ET	1.82E+00	4.08E+00	3.75E+01	1.92E+00	1.91E+00	1.47E+01	2.16E+00	2.20E+00	2.17E+00	2.08E+00	2.33E+00	2.43E+00
		NFEs	6.60E+04	6.60E+04	6.63E+04	6.62E+04	6.60E+04	6.61E+04	6.61E+04	6.63E+04	6.61E+04	6.61E+04	6.61E+04	6.61E+04
	IP-SP	Min	5.40E+03	4.45E+02	6.00E+02	8.30E+02	9.07E+02	1.91E+03	2.03E+03	2.22E+03	2.48E+03	2.42E+03	2.60E+03	2.94E+03
		Max	2.52E+04	4.76E+02	6.02E+02	8.94E+02	3.07E+03	1.29E+04	2.12E+03	2.34E+03	2.50E+03	2.90E+03	3.00E+03	3.06E+03
		Mean	1.49E+04	4.62E+02	6.00E+02	8.57E+02	1.37E+03	5.58E+03	2.05E+03	2.24E+03	2.49E+03	2.57E+03	2.90E+03	2.98E+03
		STD	1.56E+04	6.30E+01	6.35E-01	5.94E+01	6.92E+02	4.93E+03	5.28E+01	5.86E+01	1.88E+02	2.15E+02	3.17E+02	2.77E+02
		ET	1.85E+00	1.87E+00	4.19E+00	1.92E+00	1.92E+00	1.90E+00	2.23E+00	2.19E+00	2.16E+00	2.07E+00	2.32E+00	2.37E+00
		NFEs	6.61E+04	6.60E+04	6.62E+04	6.60E+04	6.61E+04	6.62E+04	6.60E+04	6.61E+04	6.60E+04	6.60E+04	6.61E+04	6.61E+04

Table 4 continued from previous page

			F_1	F_2	F_3	F_4	F_5	F_6	F_7	F_8	F_9	F_{10}	F_{11}	F_{12}
QROBL-GA	IP	Min	3.88E+03	4.36E+02	6.00E+02	8.31E+02	9.25E+02	2.11E+03	2.02E+03	2.22E+03	2.48E+03	2.42E+03	2.90E+03	2.95E+03
		Max	2.65E+04	4.76E+02	6.05E+02	8.77E+02	1.47E+03	1.48E+04	2.18E+03	2.36E+03	2.50E+03	2.88E+03	3.00E+03	3.04E+03
		Mean	1.02E+04	4.59E+02	6.01E+02	8.50E+02	1.12E+03	4.92E+03	2.06E+03	2.24E+03	2.49E+03	2.61E+03	2.92E+03	2.97E+03
		STD	1.11E+04	6.00E+01	1.26E+00	5.17E+01	2.67E+02	4.26E+03	6.97E+01	5.89E+01	1.88E+02	2.42E+02	3.21E+02	2.75E+02
		ET	1.39E+00	1.43E+00	9.51E+00	1.50E+00	1.45E+00	1.42E+00	9.26E+00	1.72E+00	1.67E+00	1.58E+00	2.10E+00	4.87E+00
		NFEs	5.11E+04	5.11E+04	5.11E+04	5.11E+04	5.11E+04	5.11E+04	5.11E+04	5.11E+04	5.11E+04	5.11E+04	5.11E+04	5.11E+04
	SP	Min	2.21E+03	4.45E+02	6.00E+02	8.21E+02	9.38E+02	1.88E+03	2.02E+03	2.22E+03	2.48E+03	2.50E+03	2.90E+03	2.94E+03
		Max	2.01E+04	4.76E+02	6.02E+02	8.85E+02	1.92E+03	1.40E+04	2.14E+03	2.34E+03	2.50E+03	2.89E+03	3.00E+03	3.01E+03
		Mean	7.72E+03	4.59E+02	6.01E+02	8.50E+02	1.21E+03	5.04E+03	2.05E+03	2.25E+03	2.49E+03	2.62E+03	2.92E+03	2.97E+03
		STD	8.26E+03	5.99E+01	7.00E-01	5.22E+01	3.84E+02	4.45E+03	5.90E+01	6.31E+01	1.88E+02	2.59E+02	3.20E+02	2.71E+02
		ET	1.82E+00	1.81E+00	4.29E+00	2.10E+00	1.91E+00	1.86E+00	6.47E+01	2.29E+00	2.15E+00	2.12E+00	7.59E+00	2.34E+00
		NFEs	6.61E+04	6.60E+04	6.61E+04	6.61E+04	6.60E+04	6.62E+04	6.62E+04	6.63E+04	6.62E+04	6.58E+04	6.61E+04	6.61E+04
	IP-SP	Min	2.85E+03	4.29E+02	6.00E+02	8.36E+02	9.21E+02	1.89E+03	2.02E+03	2.22E+03	2.48E+03	2.41E+03	2.90E+03	2.94E+03
		Max	1.62E+04	4.76E+02	6.04E+02	8.81E+02	2.19E+03	1.01E+04	2.17E+03	2.34E+03	2.50E+03	2.89E+03	3.00E+03	3.08E+03
		Mean	7.00E+03	4.55E+02	6.01E+02	8.54E+02	1.37E+03	4.58E+03	2.05E+03	2.24E+03	2.49E+03	2.59E+03	2.92E+03	2.97E+03
		STD	7.33E+03	5.59E+01	1.14E+00	5.55E+01	6.40E+02	3.55E+03	5.73E+01	5.61E+01	1.87E+02	2.27E+02	3.16E+02	2.75E+02
		ET	3.28E+00	1.86E+00	2.13E+00	2.36E+00	1.96E+00	1.86E+00	4.78E+00	2.20E+00	2.18E+00	8.50E+00	2.31E+00	2.35E+00
		NFEs	6.62E+04	6.60E+04	6.63E+04	6.59E+04	6.61E+04	6.62E+04	6.58E+04	6.60E+04	6.63E+04	6.62E+04	6.63E+04	6.61E+04

Table 5: Performance metrics for PSO variants across 12 functions ($D = 10$)

			F_1	F_2	F_3	F_4	F_5	F_6	F_7	F_8	F_9	F_{10}	F_{11}	F_{12}
PSO		Min	3.00E+02	4.00E+02	6.00E+02	8.05E+02	9.00E+02	1.82E+03	2.00E+03	2.20E+03	2.53E+03	2.50E+03	2.60E+03	2.86E+03
		Max	3.00E+02	4.09E+02	6.06E+02	8.33E+02	9.02E+02	7.87E+03	2.06E+03	2.22E+03	2.53E+03	2.63E+03	3.00E+03	2.88E+03
		Mean	3.00E+02	4.04E+02	6.01E+02	8.15E+02	9.00E+02	3.17E+03	2.03E+03	2.22E+03	2.53E+03	2.57E+03	2.69E+03	2.87E+03
		STD	4.52E-14	5.07E+00	1.50E+00	1.66E+01	5.33E-01	2.10E+03	3.29E+01	2.05E+01	2.29E+02	1.78E+02	1.65E+02	1.67E+02
		ET	7.31E-01	6.65E-01	7.25E-01	6.65E-01	6.77E-01	6.65E-01	8.08E-01	8.18E-01	7.47E-01	7.28E-01	8.32E-01	8.86E-01
		NFEs	5.00E+04	5.00E+04	5.00E+04	5.00E+04	5.00E+04	5.00E+04	5.00E+04	5.00E+04	5.00E+04	5.00E+04	5.00E+04	5.00E+04
BOBL-PSO	IP	Min	3.00E+02	4.00E+02	6.00E+02	8.05E+02	9.00E+02	1.81E+03	2.00E+03	2.20E+03	2.53E+03	2.50E+03	2.60E+03	2.86E+03
		Max	3.00E+02	4.09E+02	6.10E+02	8.29E+02	9.02E+02	7.85E+03	2.06E+03	2.22E+03	2.68E+03	2.69E+03	3.18E+03	2.91E+03
		Mean	3.00E+02	4.03E+02	6.02E+02	8.15E+02	9.00E+02	3.97E+03	2.02E+03	2.22E+03	2.53E+03	2.57E+03	2.75E+03	2.87E+03
		STD	4.87E-14	5.02E+00	3.40E+00	1.64E+01	6.62E-01	2.91E+03	2.82E+01	2.01E+01	2.36E+02	1.82E+02	2.38E+02	1.68E+02
		ET	8.29E-01	7.87E-01	8.81E-01	8.22E-01	8.15E-01	7.93E-01	9.18E-01	8.39E+00	9.16E-01	8.88E-01	1.01E+00	9.95E-01
		NFEs	5.01E+04	5.01E+04	5.01E+04	5.01E+04	5.01E+04	5.01E+04	5.01E+04	5.01E+04	5.01E+04	5.01E+04	5.01E+04	5.01E+04
	SP	Min	3.00E+02	4.00E+02	6.00E+02	8.04E+02	9.00E+02	1.81E+03	2.00E+03	2.20E+03	2.53E+03	2.50E+03	2.60E+03	2.86E+03
		Max	3.00E+02	4.09E+02	6.11E+02	8.27E+02	9.03E+02	7.88E+03	2.05E+03	2.34E+03	2.53E+03	2.63E+03	3.00E+03	2.88E+03
		Mean	3.00E+02	4.03E+02	6.01E+02	8.14E+02	9.00E+02	3.85E+03	2.03E+03	2.22E+03	2.53E+03	2.56E+03	2.72E+03	2.87E+03
		STD	5.08E-14	4.17E+00	2.20E+00	1.08E+01	7.69E-01	2.81E+03	2.72E+01	3.05E+01	0.00E+00	8.15E+01	1.86E+02	5.62E+00
		ET	1.65E+00	2.20E+00	2.21E+00	2.15E+00	1.80E+00	1.44E+00	1.76E+00	1.64E+00	1.60E+00	1.45E+00	1.15E+00	1.21E+00
		NFEs	6.49E+04	6.52E+04	6.51E+04	6.52E+04	6.49E+04	6.50E+04	6.50E+04	6.50E+04	6.49E+04	6.49E+04	6.48E+04	6.51E+04
	IP-SP	Min	3.00E+02	4.00E+02	6.00E+02	8.05E+02	9.00E+02	1.81E+03	2.00E+03	2.22E+03	2.53E+03	2.50E+03	2.60E+03	2.86E+03
		Max	3.00E+02	4.71E+02	6.02E+02	8.30E+02	9.03E+02	7.84E+03	2.04E+03	2.34E+03	2.53E+03	2.62E+03	3.00E+03	2.89E+03
		Mean	3.00E+02	4.09E+02	6.00E+02	8.13E+02	9.00E+02	3.73E+03	2.02E+03	2.22E+03	2.53E+03	2.55E+03	2.64E+03	2.87E+03
		STD	4.64E-14	2.25E+01	8.15E-01	1.42E+01	6.67E-01	2.60E+03	2.51E+01	3.26E+01	2.29E+02	1.56E+02	1.20E+02	1.68E+02
		ET	6.04E-01	6.39E-01	7.13E-01	6.59E-01	6.58E-01	6.47E-01	7.63E-01	8.38E-01	7.23E-01	7.42E-01	7.99E-01	8.11E-01
		NFEs	6.52E+04	6.52E+04	6.52E+04	6.50E+04	6.51E+04	6.53E+04	6.51E+04	6.53E+04	6.51E+04	6.52E+04	6.51E+04	6.54E+04

Table 5 continued from previous page

			F_1	F_2	F_3	F_4	F_5	F_6	F_7	F_8	F_9	F_{10}	F_{11}	F_{12}
COOBL-PSO	IP	Min	3.00E+02	4.00E+02	6.00E+02	8.05E+02	9.00E+02	1.89E+03	2.00E+03	2.20E+03	2.53E+03	2.50E+03	2.60E+03	2.86E+03
		Max	3.00E+02	4.09E+02	6.08E+02	8.44E+02	9.03E+02	7.38E+03	2.06E+03	2.34E+03	2.53E+03	2.89E+03	3.00E+03	3.08E+03
		Mean	3.00E+02	4.04E+02	6.01E+02	8.16E+02	9.00E+02	3.27E+03	2.03E+03	2.24E+03	2.53E+03	2.58E+03	2.66E+03	2.90E+03
		STD	5.19E-14	5.40E+00	2.08E+00	1.83E+01	7.53E-01	2.05E+03	2.77E+01	5.46E+01	2.29E+02	2.00E+02	1.37E+02	2.06E+02
		ET	6.42E-01	7.99E-01	8.38E-01	8.08E-01	9.06E-01	7.90E-01	7.83E-01	7.97E-01	7.35E-01	7.32E-01	8.17E-01	8.10E-01
		NFEs	5.01E+04	5.01E+04	5.01E+04	5.01E+04	5.01E+04	5.01E+04	5.01E+04	5.01E+04	5.01E+04	5.01E+04	5.01E+04	5.01E+04
	SP	Min	3.00E+02	4.00E+02	6.00E+02	8.06E+02	9.00E+02	1.80E+03	2.00E+03	2.22E+03	2.53E+03	2.50E+03	2.60E+03	2.86E+03
		Max	3.00E+02	4.71E+02	6.16E+02	8.49E+02	1.25E+03	8.07E+03	2.13E+03	2.26E+03	2.68E+03	3.39E+03	3.00E+03	2.92E+03
		Mean	3.00E+02	4.07E+02	6.06E+02	8.21E+02	9.38E+02	3.32E+03	2.04E+03	2.22E+03	2.53E+03	2.62E+03	2.71E+03	2.87E+03
		STD	3.86E-13	1.43E+01	7.46E+00	2.33E+01	8.55E+01	2.30E+03	4.99E+01	2.34E+01	2.36E+02	3.05E+02	1.69E+02	1.69E+02
		ET	7.71E-01	7.82E-01	8.73E-01	7.97E-01	8.67E-01	1.39E+01	8.86E-01	9.89E-01	8.74E-01	8.65E-01	9.86E-01	9.82E-01
		FES	6.50E+04	6.50E+04	6.53E+04	6.49E+04	6.51E+04	6.53E+04	6.48E+04	6.50E+04	6.50E+04	6.52E+04	6.51E+04	6.50E+04
	IP-SP	Min	3.00E+02	4.00E+02	6.00E+02	8.07E+02	9.00E+02	1.83E+03	2.02E+03	2.20E+03	2.53E+03	2.50E+03	2.60E+03	2.86E+03
		Max	3.00E+02	4.09E+02	6.31E+02	8.46E+02	1.32E+03	8.04E+03	2.14E+03	2.34E+03	2.68E+03	3.85E+03	3.00E+03	2.99E+03
		Mean	3.00E+02	4.05E+02	6.05E+02	8.20E+02	9.27E+02	3.95E+03	2.05E+03	2.23E+03	2.53E+03	2.61E+03	2.72E+03	2.89E+03
		STD	2.70E-13	6.10E+00	9.71E+00	2.22E+01	8.16E+01	2.93E+03	5.31E+01	3.41E+01	2.36E+02	3.16E+02	1.84E+02	1.96E+02
		ET	7.59E-01	7.76E-01	8.58E-01	7.70E-01	7.94E-01	7.76E-01	8.83E-01	1.41E+01	8.70E-01	8.68E-01	9.70E-01	9.72E-01
		NFEs	6.51E+04	6.52E+04	6.51E+04	6.52E+04	6.50E+04	6.51E+04	6.50E+04	6.50E+04	6.51E+04	6.50E+04	6.48E+04	6.52E+04

Table 5 continued from previous page

			F_1	F_2	F_3	F_4	F_5	F_6	F_7	F_8	F_9	F_{10}	F_{11}	F_{12}
GOBL-PSO	IP	Min	3.00E+02	4.00E+02	6.00E+02	8.04E+02	9.00E+02	1.83E+03	2.00E+03	2.20E+03	2.53E+03	2.50E+03	2.60E+03	2.86E+03
		Max	3.00E+02	4.09E+02	6.08E+02	8.35E+02	9.03E+02	7.85E+03	2.05E+03	2.22E+03	2.53E+03	2.63E+03	3.00E+03	2.91E+03
		Mean	3.00E+02	4.03E+02	6.01E+02	8.16E+02	9.01E+02	3.66E+03	2.03E+03	2.22E+03	2.53E+03	2.55E+03	2.66E+03	2.87E+03
		STD	3.60E-14	4.06E+00	1.92E+00	1.86E+01	8.32E-01	2.70E+03	2.89E+01	1.96E+01	2.29E+02	1.57E+02	1.43E+02	1.68E+02
		ET	5.79E-01	5.31E-01	7.38E-01	5.67E-01	5.17E-01	4.99E-01	5.83E-01	6.19E-01	5.81E-01	5.66E-01	6.45E-01	6.49E-01
		FES	5.01E+04	5.01E+04	5.01E+04	5.01E+04	5.01E+04	5.01E+04	5.01E+04	5.01E+04	5.01E+04	5.01E+04	5.01E+04	5.01E+04
	SP	Min	3.00E+02	4.00E+02	6.00E+02	8.03E+02	9.00E+02	1.82E+03	2.00E+03	2.20E+03	2.53E+03	2.50E+03	2.60E+03	2.86E+03
		Max	3.00E+02	4.09E+02	6.08E+02	8.39E+02	9.03E+02	7.89E+03	2.05E+03	2.32E+03	2.53E+03	2.63E+03	3.00E+03	2.87E+03
		Mean	3.00E+02	4.02E+02	6.01E+02	8.15E+02	9.00E+02	3.09E+03	2.02E+03	2.22E+03	2.53E+03	2.56E+03	2.70E+03	2.87E+03
		STD	5.39E-14	3.19E+00	1.87E+00	1.71E+01	6.27E-01	2.11E+03	2.79E+01	2.93E+01	2.29E+02	1.69E+02	1.76E+02	1.66E+02
		ET	5.39E-01	5.54E-01	6.13E-01	5.72E-01	5.78E-01	5.60E-01	6.64E-01	7.05E-01	6.44E-01	6.35E-01	7.17E-01	7.24E-01
		NFEs	6.51E+04	6.48E+04	6.52E+04	6.51E+04	6.50E+04	6.51E+04	6.50E+04	6.50E+04	6.50E+04	6.53E+04	6.50E+04	6.50E+04
	IP-SP	Min	3.00E+02	4.00E+02	6.00E+02	8.04E+02	9.00E+02	1.81E+03	2.00E+03	2.20E+03	2.53E+03	2.50E+03	2.60E+03	2.86E+03
		Max	3.00E+02	4.09E+02	6.03E+02	8.33E+02	1.14E+03	7.85E+03	2.05E+03	2.34E+03	2.53E+03	2.62E+03	3.00E+03	2.95E+03
		Mean	3.00E+02	4.03E+02	6.00E+02	8.15E+02	9.08E+02	3.17E+03	2.02E+03	2.22E+03	2.53E+03	2.56E+03	2.64E+03	2.87E+03
		STD	5.08E-14	5.18E+00	8.31E-01	1.65E+01	4.32E+01	2.05E+03	2.70E+01	3.23E+01	2.29E+02	1.67E+02	1.10E+02	1.70E+02
		ET	5.76E-01	5.76E-01	6.45E-01	6.00E-01	1.76E+01	5.36E-01	6.43E-01	6.90E-01	6.31E-01	6.26E-01	7.10E-01	7.23E-01
		NFEs	6.51E+04	6.53E+04	6.52E+04	6.49E+04	6.50E+04	6.49E+04	6.53E+04	6.52E+04	6.50E+04	6.55E+04	6.50E+04	6.52E+04

Table 5 continued from previous page

			F_1	F_2	F_3	F_4	F_5	F_6	F_7	F_8	F_9	F_{10}	F_{11}	F_{12}
QOBL-PSO	IP	Min	3.00E+02	4.00E+02	6.00E+02	8.07E+02	9.00E+02	1.81E+03	2.00E+03	2.20E+03	2.53E+03	2.50E+03	2.60E+03	2.86E+03
		Max	3.00E+02	4.71E+02	6.06E+02	8.32E+02	9.03E+02	7.83E+03	2.04E+03	2.22E+03	2.68E+03	2.63E+03	2.90E+03	2.87E+03
		Mean	3.00E+02	4.06E+02	6.01E+02	8.18E+02	9.00E+02	2.99E+03	2.02E+03	2.22E+03	2.54E+03	2.57E+03	2.65E+03	2.87E+03
		STD	5.29E-14	1.40E+01	1.68E+00	1.98E+01	6.58E-01	1.95E+03	2.62E+01	2.00E+01	2.42E+02	1.83E+02	1.06E+02	1.66E+02
		ET	5.25E-01	6.10E-01	5.84E-01	4.96E-01	5.12E-01	5.14E-01	5.94E-01	6.31E-01	5.88E-01	5.90E-01	6.64E-01	6.92E-01
		FES	5.01E+04	5.01E+04	5.01E+04	5.01E+04	5.01E+04	5.01E+04	5.01E+04	5.01E+04	5.01E+04	5.01E+04	5.01E+04	5.01E+04
	SP	Min	3.00E+02	4.00E+02	6.00E+02	8.06E+02	9.00E+02	1.81E+03	2.00E+03	2.20E+03	2.53E+03	2.50E+03	2.60E+03	2.86E+03
		Max	3.00E+02	4.09E+02	6.16E+02	8.35E+02	9.01E+02	7.92E+03	2.05E+03	2.34E+03	2.68E+03	2.62E+03	3.18E+03	2.87E+03
		Mean	3.00E+02	4.03E+02	6.02E+02	8.19E+02	9.00E+02	3.59E+03	2.02E+03	2.22E+03	2.53E+03	2.56E+03	2.68E+03	2.87E+03
		STD	4.52E-14	5.03E+00	3.36E+00	2.01E+01	5.70E-01	2.71E+03	2.67E+01	3.19E+01	2.36E+02	1.68E+02	1.78E+02	1.66E+02
		ET	7.02E-01	7.29E-01	8.01E-01	7.51E-01	7.66E-01	7.49E-01	8.55E-01	9.02E-01	8.66E-01	8.64E-01	9.63E-01	9.72E-01
		NFEs	6.51E+04	6.50E+04	6.50E+04	6.51E+04	6.50E+04	6.50E+04	6.50E+04	6.49E+04	6.49E+04	6.50E+04	6.53E+04	6.50E+04
	IP-SP	Min	3.00E+02	4.00E+02	6.00E+02	8.05E+02	9.00E+02	1.80E+03	2.00E+03	2.22E+03	2.53E+03	2.50E+03	2.60E+03	2.86E+03
		Max	3.00E+02	4.71E+02	6.11E+02	8.54E+02	9.01E+02	7.87E+03	2.05E+03	2.34E+03	2.53E+03	2.63E+03	2.90E+03	3.06E+03
		Mean	3.00E+02	4.06E+02	6.01E+02	8.19E+02	9.00E+02	2.94E+03	2.03E+03	2.23E+03	2.53E+03	2.56E+03	2.68E+03	2.88E+03
		STD	4.40E-14	1.39E+01	2.97E+00	2.09E+01	3.62E-01	2.04E+03	3.11E+01	4.11E+01	2.29E+02	1.68E+02	1.37E+02	1.80E+02
		ET	8.01E-01	7.96E-01	8.58E-01	8.04E-01	8.04E-01	7.94E-01	8.91E-01	9.55E-01	9.58E-01	1.02E+00	9.09E-01	9.57E-01
		NFEs	6.51E+04	6.55E+04	6.50E+04	6.52E+04	6.49E+04	6.50E+04	6.48E+04	6.53E+04	6.48E+04	6.52E+04	6.53E+04	6.51E+04

Table 5 continued from previous page

			F_1	F_2	F_3	F_4	F_5	F_6	F_7	F_8	F_9	F_{10}	F_{11}	F_{12}
QROBL-PSO	IP	Min	3.00E+02	4.00E+02	6.00E+02	8.09E+02	9.00E+02	1.81E+03	2.00E+03	2.20E+03	2.53E+03	2.50E+03	2.60E+03	2.86E+03
		Max	3.00E+02	4.09E+02	6.11E+02	8.39E+02	9.01E+02	7.86E+03	2.04E+03	2.34E+03	2.68E+03	2.63E+03	3.00E+03	2.94E+03
		Mean	3.00E+02	4.02E+02	6.01E+02	8.18E+02	9.00E+02	3.22E+03	2.02E+03	2.23E+03	2.53E+03	2.59E+03	2.65E+03	2.87E+03
		STD	5.87E-14	3.29E+00	2.76E+00	1.85E+01	4.12E-01	2.39E+03	2.59E+01	4.07E+01	2.36E+02	1.96E+02	1.24E+02	1.72E+02
		ET	6.84E-01	6.71E-01	7.20E-01	6.50E-01	6.63E-01	6.49E-01	7.45E-01	7.93E-01	7.32E-01	7.22E-01	6.67E+01	8.23E-01
		NFEs	5.01E+04	5.01E+04	5.01E+04	5.01E+04	5.01E+04	5.01E+04	5.01E+04	5.01E+04	5.01E+04	5.01E+04	5.01E+04	5.01E+04
	SP	Min	3.00E+02	4.00E+02	6.00E+02	8.09E+02	9.00E+02	1.82E+03	2.00E+03	2.22E+03	2.53E+03	2.50E+03	2.60E+03	2.86E+03
		Max	3.00E+02	4.09E+02	6.08E+02	8.40E+02	9.08E+02	7.86E+03	2.07E+03	2.22E+03	2.68E+03	2.99E+03	3.00E+03	2.96E+03
		Mean	3.00E+02	4.03E+02	6.01E+02	8.19E+02	9.01E+02	3.27E+03	2.03E+03	2.22E+03	2.53E+03	2.57E+03	2.72E+03	2.87E+03
		STD	5.39E-14	4.04E+00	2.56E+00	2.06E+01	1.81E+00	2.24E+03	2.95E+01	2.09E+01	2.36E+02	1.93E+02	1.94E+02	1.72E+02
		ET	9.64E-01	5.20E+00	1.04E+00	9.64E-01	9.68E-01	9.47E-01	1.08E+00	1.16E+00	1.05E+00	1.07E+00	1.17E+00	1.17E+00
		NFEs	6.51E+04	6.51E+04	6.51E+04	6.50E+04	6.50E+04	6.50E+04	6.52E+04	6.51E+04	6.50E+04	6.49E+04	6.51E+04	6.50E+04
	IP-SP	Min	3.00E+02	4.00E+02	6.00E+02	8.04E+02	9.00E+02	1.83E+03	2.00E+03	2.22E+03	2.53E+03	2.50E+03	2.60E+03	2.86E+03
		Max	3.00E+02	4.09E+02	6.12E+02	8.40E+02	1.37E+03	7.89E+03	2.05E+03	2.34E+03	2.53E+03	2.62E+03	3.00E+03	2.96E+03
		Mean	3.00E+02	4.03E+02	6.01E+02	8.19E+02	9.17E+02	3.55E+03	2.03E+03	2.23E+03	2.53E+03	2.57E+03	2.68E+03	2.87E+03
		STD	4.87E-14	4.98E+00	2.62E+00	2.07E+01	8.54E+01	2.84E+03	2.93E+01	4.80E+01	2.29E+02	1.77E+02	1.60E+02	1.73E+02
		ET	9.82E-01	9.49E-01	1.04E+00	7.09E+00	9.85E-01	9.48E-01	1.08E+00	1.14E+00	1.06E+00	5.97E+00	1.21E+00	1.17E+00
		NFEs	6.50E+04	6.50E+04	6.52E+04	6.51E+04	6.50E+04	6.49E+04	6.51E+04	6.52E+04	6.50E+04	6.51E+04	6.52E+04	6.51E+04

Table 6: Performance metrics for PSO variants across 12 functions ($D = 20$)

			F_1	F_2	F_3	F_4	F_5	F_6	F_7	F_8	F_9	F_{10}	F_{11}	F_{12}
PSO		Min	3.00E+02	4.00E+02	6.00E+02	8.35E+02	9.01E+02	1.90E+03	2.04E+03	2.22E+03	2.48E+03	2.50E+03	2.60E+03	2.95E+03
		Max	3.00E+02	4.74E+02	6.45E+02	8.95E+02	2.75E+03	2.03E+04	2.28E+03	2.36E+03	2.48E+03	5.24E+03	3.36E+03	3.12E+03
		Mean	3.00E+02	4.45E+02	6.10E+02	8.57E+02	1.15E+03	5.25E+03	2.12E+03	2.28E+03	2.48E+03	3.40E+03	2.92E+03	3.00E+03
		STD	3.06E-02	5.02E+01	1.54E+01	5.87E+01	4.80E+02	5.59E+03	1.35E+02	9.76E+01	1.81E+02	1.26E+03	3.50E+02	3.00E+02
		ET	6.75E-01	2.30E+01	8.88E-01	7.30E-01	7.67E-01	7.16E-01	9.09E-01	9.57E-01	9.84E-01	1.50E+02	1.10E+00	1.09E+00
		NFEs	5.00E+04	5.00E+04	5.00E+04	5.00E+04	5.00E+04	5.00E+04	5.00E+04	5.00E+04	5.00E+04	5.00E+04	5.00E+04	5.00E+04
BOBL-PSO	IP	Min	3.00E+02	4.00E+02	6.00E+02	8.31E+02	9.01E+02	1.83E+03	2.02E+03	2.22E+03	2.48E+03	2.50E+03	2.60E+03	2.94E+03
		Max	3.00E+02	4.72E+02	6.42E+02	8.86E+02	2.38E+03	2.02E+04	2.29E+03	2.34E+03	2.48E+03	7.41E+03	3.00E+03	4.55E+03
		Mean	3.00E+02	4.46E+02	6.13E+02	8.54E+02	1.24E+03	5.73E+03	2.10E+03	2.25E+03	2.48E+03	3.57E+03	2.93E+03	3.25E+03
		STD	9.48E-02	5.15E+01	1.64E+01	5.58E+01	5.30E+02	6.49E+03	1.11E+02	6.58E+01	1.81E+02	1.48E+03	3.42E+02	7.42E+02
		ET	8.17E-01	8.47E-01	1.07E+00	8.82E-01	9.13E-01	8.55E-01	1.11E+00	1.15E+00	5.11E+00	1.04E+00	1.26E+00	1.30E+00
		NFEs	5.01E+04	5.01E+04	5.01E+04	5.01E+04	5.01E+04	5.01E+04	5.01E+04	5.01E+04	5.01E+04	5.01E+04	5.01E+04	5.01E+04
	SP	Min	3.00E+02	4.00E+02	6.00E+02	8.31E+02	9.02E+02	1.88E+03	2.04E+03	2.22E+03	2.48E+03	2.50E+03	2.60E+03	2.95E+03
		Max	3.00E+02	4.73E+02	6.25E+02	8.90E+02	2.10E+03	1.77E+04	2.23E+03	2.36E+03	2.48E+03	4.72E+03	3.36E+03	3.07E+03
		Mean	3.00E+02	4.50E+02	6.07E+02	8.55E+02	1.06E+03	5.75E+03	2.10E+03	2.26E+03	2.48E+03	3.12E+03	2.93E+03	3.00E+03
		STD	2.65E-02	5.51E+01	9.77E+00	5.67E+01	3.09E+02	5.93E+03	1.11E+02	7.69E+01	1.81E+02	1.01E+03	3.48E+02	3.01E+02
		ET	1.04E+00	9.90E-01	1.26E+00	1.07E+00	1.07E+00	9.92E-01	1.25E+00	1.65E+00	1.92E+00	7.87E-01	9.93E-01	1.04E+00
		NFEs	6.50E+04	6.50E+04	6.51E+04	6.52E+04	6.51E+04	6.51E+04	6.51E+04	6.49E+04	6.51E+04	6.51E+04	6.48E+04	6.51E+04
	IP-SP	Min	3.00E+02	4.05E+02	6.02E+02	8.30E+02	9.01E+02	1.82E+03	2.04E+03	2.22E+03	2.48E+03	2.50E+03	2.60E+03	2.95E+03
		Max	3.00E+02	4.74E+02	6.32E+02	8.91E+02	1.65E+03	1.74E+04	2.24E+03	2.46E+03	2.48E+03	5.12E+03	3.00E+03	3.06E+03
		Mean	3.00E+02	4.51E+02	6.11E+02	8.52E+02	1.00E+03	5.33E+03	2.10E+03	2.27E+03	2.48E+03	3.51E+03	2.90E+03	2.99E+03
		STD	5.42E-02	5.30E+01	1.40E+01	5.46E+01	2.29E+02	5.36E+03	1.05E+02	9.32E+01	1.81E+02	1.37E+03	3.13E+02	2.88E+02
		ET	6.69E-01	6.78E-01	8.38E-01	7.04E-01	7.37E-01	7.10E-01	8.84E-01	9.80E-01	9.11E-01	8.47E-01	1.03E+00	1.11E+00
		NFEs	6.51E+04	6.53E+04	6.51E+04	6.51E+04	6.52E+04	6.50E+04	6.50E+04	6.52E+04	6.51E+04	6.49E+04	6.53E+04	6.51E+04

Table 6 continued from previous page

			F_1	F_2	F_3	F_4	F_5	F_6	F_7	F_8	F_9	F_{10}	F_{11}	F_{12}
COOBL-PSO	IP	Min	3.00E+02	4.01E+02	6.01E+02	8.35E+02	9.05E+02	1.87E+03	2.03E+03	2.22E+03	2.48E+03	2.50E+03	2.90E+03	2.95E+03
		Max	3.01E+02	4.74E+02	6.49E+02	8.94E+02	2.20E+03	1.80E+04	2.24E+03	2.46E+03	2.48E+03	4.77E+03	3.00E+03	3.80E+03
		Mean	3.00E+02	4.49E+02	6.10E+02	8.52E+02	1.11E+03	6.27E+03	2.10E+03	2.28E+03	2.48E+03	3.58E+03	2.93E+03	3.11E+03
		STD	1.70E-01	5.36E+01	1.45E+01	5.34E+01	3.85E+02	6.32E+03	1.07E+02	1.00E+02	1.81E+02	1.35E+03	3.30E+02	4.39E+02
		ET	6.57E-01	6.96E-01	2.04E+01	7.37E-01	7.49E-01	7.46E-01	9.04E-01	9.58E-01	9.23E-01	8.66E-01	2.02E+00	1.06E+00
		NFEs	5.01E+04	5.01E+04	5.01E+04	5.01E+04	5.01E+04	5.01E+04	5.01E+04	5.01E+04	5.01E+04	5.01E+04	5.01E+04	5.01E+04
	SP	Min	3.00E+02	4.00E+02	6.04E+02	8.38E+02	9.66E+02	1.96E+03	2.03E+03	2.22E+03	2.48E+03	2.50E+03	2.90E+03	2.95E+03
		Max	1.04E+03	4.71E+02	6.60E+02	9.30E+02	2.38E+03	1.94E+04	2.33E+03	2.56E+03	2.48E+03	6.01E+03	4.39E+03	3.21E+03
		Mean	3.25E+02	4.42E+02	6.27E+02	8.65E+02	1.65E+03	6.60E+03	2.12E+03	2.28E+03	2.48E+03	3.95E+03	2.98E+03	3.03E+03
		STD	1.35E+02	4.70E+01	3.11E+01	6.79E+01	8.77E+02	7.16E+03	1.36E+02	1.09E+02	1.81E+02	1.83E+03	4.63E+02	3.35E+02
		ET	8.14E-01	8.21E-01	1.07E+00	8.45E-01	9.13E-01	8.64E-01	2.13E+00	1.17E+00	1.11E+00	1.00E+00	1.25E+00	1.29E+00
		NFEs	6.48E+04	6.52E+04	6.53E+04	6.51E+04	6.51E+04	6.50E+04	6.50E+04	6.50E+04	6.51E+04	6.50E+04	6.50E+04	6.48E+04
	IP-SP	Min	3.00E+02	4.00E+02	6.12E+02	8.28E+02	9.38E+02	1.87E+03	2.06E+03	2.22E+03	2.48E+03	2.50E+03	2.60E+03	2.97E+03
		Max	1.84E+04	4.72E+02	6.51E+02	9.33E+02	6.79E+03	2.04E+04	2.25E+03	2.49E+03	2.48E+03	5.32E+03	3.35E+03	4.00E+03
		Mean	1.04E+03	4.43E+02	6.32E+02	8.71E+02	1.84E+03	6.32E+03	2.13E+03	2.29E+03	2.48E+03	3.77E+03	2.94E+03	3.25E+03
		STD	3.34E+03	4.72E+01	3.40E+01	7.45E+01	1.44E+03	6.83E+03	1.36E+02	1.11E+02	1.81E+02	1.62E+03	3.59E+02	5.97E+02
		ET	8.03E-01	8.01E-01	3.48E+00	8.56E-01	9.11E-01	8.48E-01	1.06E+00	1.13E+00	1.10E+00	9.96E-01	1.24E+00	1.26E+00
		NFEs	6.53E+04	6.50E+04	6.51E+04	6.51E+04	6.52E+04	6.54E+04	6.49E+04	6.53E+04	6.49E+04	6.53E+04	6.52E+04	6.51E+04

Table 6 continued from previous page

			F_1	F_2	F_3	F_4	F_5	F_6	F_7	F_8	F_9	F_{10}	F_{11}	F_{12}
GOBL-PSO	IP	Min	3.00E+02	4.01E+02	6.00E+02	8.31E+02	9.03E+02	1.91E+03	2.04E+03	2.22E+03	2.48E+03	2.50E+03	2.90E+03	2.95E+03
		Max	3.04E+02	4.69E+02	6.32E+02	8.78E+02	1.62E+03	2.03E+04	2.25E+03	2.36E+03	2.48E+03	4.54E+03	3.00E+03	3.14E+03
		Mean	3.00E+02	4.37E+02	6.10E+02	8.52E+02	9.94E+02	5.20E+03	2.09E+03	2.26E+03	2.48E+03	3.42E+03	2.94E+03	3.00E+03
		STD	6.83E-01	4.32E+01	1.35E+01	5.32E+01	1.92E+02	5.17E+03	1.05E+02	7.80E+01	1.81E+02	1.25E+03	3.44E+02	3.07E+02
		ET	5.44E-01	5.74E-01	7.01E-01	5.85E-01	6.11E-01	5.82E-01	7.33E-01	7.84E-01	1.39E+02	6.17E-01	7.28E-01	7.83E-01
		NFEs	5.01E+04	5.01E+04	5.01E+04	5.01E+04	5.01E+04	5.01E+04	5.01E+04	5.01E+04	5.01E+04	5.01E+04	5.01E+04	5.01E+04
	SP	Min	3.00E+02	4.03E+02	6.00E+02	8.21E+02	9.00E+02	1.88E+03	2.03E+03	2.22E+03	2.48E+03	2.50E+03	2.90E+03	2.95E+03
		Max	3.00E+02	4.72E+02	6.42E+02	8.97E+02	2.04E+03	1.82E+04	2.23E+03	2.46E+03	2.48E+03	5.05E+03	3.00E+03	3.20E+03
		Mean	3.00E+02	4.50E+02	6.10E+02	8.55E+02	1.13E+03	4.60E+03	2.09E+03	2.29E+03	2.48E+03	3.47E+03	2.92E+03	3.01E+03
		STD	6.24E-02	5.31E+01	1.46E+01	5.71E+01	3.74E+02	4.42E+03	9.49E+01	1.13E+02	1.81E+02	1.40E+03	3.22E+02	3.17E+02
		ET	5.81E-01	1.18E+01	7.36E-01	6.11E-01	6.40E-01	6.01E-01	7.96E-01	8.46E-01	8.08E-01	7.34E-01	9.11E-01	9.59E-01
		NFEs	6.50E+04	6.52E+04	6.49E+04	6.52E+04	6.51E+04	6.48E+04	6.50E+04	6.52E+04	6.51E+04	6.49E+04	6.51E+04	6.51E+04
	IP-SP	Min	3.00E+02	4.02E+02	6.00E+02	8.27E+02	9.02E+02	1.87E+03	2.04E+03	2.22E+03	2.48E+03	2.50E+03	2.60E+03	2.94E+03
		Max	3.00E+02	4.76E+02	6.28E+02	8.92E+02	1.90E+03	1.36E+04	2.21E+03	2.34E+03	2.48E+03	4.63E+03	3.00E+03	3.06E+03
		Mean	3.00E+02	4.52E+02	6.09E+02	8.52E+02	1.08E+03	4.53E+03	2.10E+03	2.25E+03	2.48E+03	3.09E+03	2.91E+03	3.00E+03
		STD	5.90E-02	5.48E+01	1.15E+01	5.39E+01	3.06E+02	4.02E+03	1.10E+02	6.67E+01	1.81E+02	9.78E+02	3.21E+02	2.98E+02
		ET	5.77E-01	6.04E-01	1.94E+01	8.36E-01	8.63E-01	8.25E-01	1.08E+00	1.12E+00	3.51E+01	1.01E+00	1.22E+00	1.31E+00
		NFEs	6.51E+04	6.49E+04	6.50E+04	6.50E+04	6.52E+04	6.50E+04	6.52E+04	6.49E+04	6.52E+04	6.49E+04	6.51E+04	6.51E+04

Table 6 continued from previous page

			F_1	F_2	F_3	F_4	F_5	F_6	F_7	F_8	F_9	F_{10}	F_{11}	F_{12}
QOBL-PSO	IP	Min	3.00E+02	4.03E+02	6.00E+02	8.30E+02	9.02E+02	1.86E+03	2.03E+03	2.22E+03	2.48E+03	2.50E+03	2.60E+03	2.95E+03
		Max	3.00E+02	4.73E+02	6.37E+02	9.17E+02	2.29E+03	1.55E+04	2.26E+03	2.36E+03	2.48E+03	4.47E+03	3.36E+03	3.26E+03
		Mean	3.00E+02	4.47E+02	6.15E+02	8.61E+02	1.19E+03	5.62E+03	2.09E+03	2.27E+03	2.48E+03	3.24E+03	2.94E+03	3.01E+03
		STD	1.37E-02	5.09E+01	1.97E+01	6.42E+01	4.55E+02	5.67E+03	1.07E+02	8.83E+01	1.81E+02	1.12E+03	3.59E+02	3.20E+02
		ET	5.47E-01	5.72E-01	7.04E-01	6.25E-01	6.47E-01	6.85E-01	8.31E-01	1.05E+00	8.32E-01	7.07E-01	1.04E+01	9.14E-01
		NFEs	5.01E+04	5.01E+04	5.01E+04	5.01E+04	5.01E+04	5.01E+04	5.01E+04	5.01E+04	5.01E+04	5.01E+04	5.01E+04	5.01E+04
	SP	Min	3.00E+02	4.01E+02	6.00E+02	8.27E+02	9.03E+02	1.87E+03	2.04E+03	2.22E+03	2.48E+03	2.50E+03	2.90E+03	2.95E+03
		Max	3.00E+02	4.73E+02	6.49E+02	8.93E+02	2.40E+03	1.93E+04	2.24E+03	2.36E+03	2.48E+03	4.78E+03	3.00E+03	3.04E+03
		Mean	3.00E+02	4.53E+02	6.09E+02	8.58E+02	1.22E+03	5.18E+03	2.12E+03	2.26E+03	2.48E+03	3.59E+03	2.93E+03	2.99E+03
		STD	3.86E-02	5.58E+01	1.29E+01	6.07E+01	5.40E+02	5.29E+03	1.30E+02	8.52E+01	1.81E+02	1.41E+03	3.30E+02	2.91E+02
		ET	1.59E+00	9.38E-01	1.15E+00	1.01E+00	1.04E+00	1.00E+00	1.20E+00	1.27E+00	1.23E+00	1.16E+00	1.32E+00	1.36E+00
		NFEs	6.49E+04	6.50E+04	6.50E+04	6.50E+04	6.51E+04	6.52E+04	6.51E+04	6.52E+04	6.51E+04	6.53E+04	6.51E+04	6.50E+04
	IP-SP	Min	3.00E+02	4.00E+02	6.01E+02	8.30E+02	9.01E+02	1.85E+03	2.04E+03	2.22E+03	2.48E+03	2.50E+03	2.60E+03	2.95E+03
		Max	3.00E+02	4.73E+02	6.64E+02	9.06E+02	2.05E+03	1.83E+04	2.20E+03	2.36E+03	2.48E+03	4.53E+03	3.00E+03	3.14E+03
		Mean	3.00E+02	4.49E+02	6.16E+02	8.64E+02	1.26E+03	6.62E+03	2.10E+03	2.26E+03	2.48E+03	3.22E+03	2.92E+03	3.00E+03
		STD	3.43E-02	5.22E+01	2.11E+01	6.62E+01	5.19E+02	6.79E+03	1.07E+02	7.72E+01	1.81E+02	1.07E+03	3.37E+02	3.04E+02
		ET	9.16E-01	9.70E-01	1.15E+00	1.01E+00	1.05E+00	1.01E+00	1.24E+00	3.02E+00	1.23E+00	1.13E+00	1.32E+00	1.39E+00
		NFEs	6.51E+04	6.52E+04	6.54E+04	6.51E+04	6.52E+04	6.52E+04	6.51E+04	6.54E+04	6.51E+04	6.51E+04	6.53E+04	6.50E+04

Table 6 continued from previous page

			F_1	F_2	F_3	F_4	F_5	F_6	F_7	F_8	F_9	F_{10}	F_{11}	F_{12}
QROBL-PSO	IP	Min	3.00E+02	4.01E+02	6.00E+02	8.21E+02	9.00E+02	1.89E+03	2.03E+03	2.22E+03	2.48E+03	2.50E+03	2.60E+03	2.95E+03
		Max	3.00E+02	4.73E+02	6.43E+02	9.14E+02	2.68E+03	1.19E+04	2.29E+03	2.46E+03	2.48E+03	4.35E+03	3.00E+03	3.27E+03
		Mean	3.00E+02	4.48E+02	6.15E+02	8.59E+02	1.44E+03	5.23E+03	2.11E+03	2.26E+03	2.48E+03	3.08E+03	2.91E+03	3.01E+03
		STD	9.70E-03	5.14E+01	1.89E+01	6.22E+01	7.94E+02	4.74E+03	1.20E+02	8.50E+01	1.81E+02	9.45E+02	3.21E+02	3.14E+02
		ET	7.01E-01	6.94E-01	8.53E-01	7.06E-01	7.29E-01	6.89E-01	1.47E+00	9.64E-01	9.10E-01	8.25E-01	1.02E+00	1.05E+00
		NFEs	5.01E+04	5.01E+04	5.01E+04	5.01E+04	5.01E+04	5.01E+04	5.01E+04	5.01E+04	5.01E+04	5.01E+04	5.01E+04	5.01E+04
	SP	Min	3.00E+02	4.00E+02	6.01E+02	8.23E+02	9.07E+02	1.84E+03	2.04E+03	2.22E+03	2.48E+03	2.50E+03	2.60E+03	2.95E+03
		Max	3.00E+02	4.74E+02	6.59E+02	8.87E+02	2.31E+03	1.04E+04	2.26E+03	2.36E+03	2.48E+03	5.04E+03	3.00E+03	3.08E+03
		Mean	3.00E+02	4.45E+02	6.14E+02	8.56E+02	1.48E+03	4.58E+03	2.11E+03	2.28E+03	2.48E+03	3.76E+03	2.89E+03	3.00E+03
		STD	7.50E-03	4.98E+01	2.05E+01	5.85E+01	7.02E+02	4.01E+03	1.24E+02	1.03E+02	1.81E+02	1.55E+03	3.08E+02	2.99E+02
		ET	4.55E+00	1.20E+00	1.40E+00	1.22E+00	1.25E+00	1.62E+00	1.47E+00	1.53E+00	1.49E+00	1.40E+00	3.25E+01	1.69E+00
		NFEs	6.49E+04	6.50E+04	6.50E+04	6.50E+04	6.50E+04	6.50E+04	6.52E+04	6.51E+04	6.53E+04	6.51E+04	6.51E+04	6.50E+04
	IP-SP	Min	3.00E+02	4.05E+02	6.01E+02	8.33E+02	9.02E+02	1.95E+03	2.04E+03	2.22E+03	2.48E+03	2.50E+03	2.60E+03	2.96E+03
		Max	3.00E+02	4.74E+02	6.46E+02	8.92E+02	2.29E+03	1.99E+04	2.24E+03	2.36E+03	2.48E+03	4.64E+03	3.00E+03	3.12E+03
		Mean	3.00E+02	4.47E+02	6.13E+02	8.59E+02	1.35E+03	5.04E+03	2.10E+03	2.25E+03	2.48E+03	3.31E+03	2.93E+03	3.00E+03
		STD	1.17E-03	5.00E+01	1.88E+01	6.03E+01	6.42E+02	4.94E+03	1.07E+02	7.27E+01	1.81E+02	1.17E+03	3.42E+02	3.02E+02
		ET	1.16E+00	1.19E+00	1.47E+00	4.92E+00	1.27E+00	1.20E+00	1.51E+00	3.51E+00	3.14E+00	1.39E+00	1.63E+00	1.69E+00
		NFEs	6.52E+04	6.52E+04	6.50E+04	6.53E+04	6.51E+04	6.53E+04	6.51E+04	6.53E+04	6.51E+04	6.52E+04	6.51E+04	6.54E+04

Table 7: Performance metrics for HS variants across 12 functions ($D = 10$)

			F_1	F_2	F_3	F_4	F_5	F_6	F_7	F_8	F_9	F_{10}	F_{11}	F_{12}
HS		Min	3.35E+02	4.00E+02	6.00E+02	8.02E+02	9.00E+02	1.81E+03	2.00E+03	2.21E+03	2.53E+03	2.40E+03	2.60E+03	2.86E+03
		Max	1.87E+03	4.69E+02	6.00E+02	8.32E+02	9.21E+02	7.20E+03	2.02E+03	2.22E+03	2.54E+03	2.62E+03	3.13E+03	2.87E+03
		Mean	6.60E+02	4.08E+02	6.00E+02	8.15E+02	9.05E+02	2.99E+03	2.01E+03	2.22E+03	2.53E+03	2.55E+03	2.71E+03	2.87E+03
		STD	4.81E+02	1.42E+01	5.20E-03	1.85E+01	7.41E+00	1.84E+03	1.53E+01	1.86E+01	2.31E+02	1.62E+02	1.76E+02	1.67E+02
		ET	5.44E-01	5.35E-01	6.04E-01	5.67E-01	5.67E-01	5.46E-01	6.43E-01	6.73E-01	6.15E-01	6.26E-01	6.84E-01	6.90E-01
		NFEs	5.00E+04	5.00E+04	5.00E+04	5.00E+04	5.00E+04	5.00E+04	5.00E+04	5.00E+04	5.00E+04	5.00E+04	5.00E+04	5.00E+04
BOBL-HS	IP	Min	3.60E+02	4.00E+02	6.00E+02	8.01E+02	9.00E+02	1.88E+03	2.00E+03	2.20E+03	2.53E+03	2.40E+03	2.60E+03	2.86E+03
		Max	1.66E+03	4.68E+02	6.00E+02	8.34E+02	9.19E+02	6.27E+03	2.02E+03	2.22E+03	2.54E+03	2.64E+03	3.00E+03	2.87E+03
		Mean	7.90E+02	4.12E+02	6.00E+02	8.13E+02	9.07E+02	3.10E+03	2.01E+03	2.22E+03	2.53E+03	2.54E+03	2.71E+03	2.87E+03
		STD	5.74E+02	2.22E+01	5.14E-02	1.72E+01	8.38E+00	1.69E+03	1.50E+01	1.94E+01	2.31E+02	1.71E+02	1.58E+02	1.67E+02
		ET	6.51E-01	6.43E-01	7.06E-01	6.65E-01	6.68E-01	6.49E-01	7.45E-01	7.75E-01	7.30E-01	7.20E-01	7.88E-01	7.96E-01
		NFEs	5.01E+04	5.01E+04	5.01E+04	5.01E+04	5.01E+04	5.01E+04	5.01E+04	5.01E+04	5.01E+04	5.01E+04	5.01E+04	5.01E+04
	SP	Min	3.45E+02	4.00E+02	6.00E+02	8.03E+02	9.01E+02	1.82E+03	2.00E+03	2.22E+03	2.53E+03	2.40E+03	2.60E+03	2.86E+03
		Max	1.10E+03	4.09E+02	6.00E+02	8.34E+02	9.28E+02	7.15E+03	2.02E+03	2.22E+03	2.54E+03	2.62E+03	2.91E+03	2.87E+03
		Mean	6.52E+02	4.05E+02	6.00E+02	8.12E+02	9.08E+02	3.28E+03	2.02E+03	2.22E+03	2.53E+03	2.52E+03	2.70E+03	2.87E+03
		STD	4.29E+02	5.83E+00	9.46E-02	1.50E+01	9.63E+00	2.07E+03	1.71E+01	2.03E+01	2.31E+02	1.49E+02	1.51E+02	1.68E+02
		ET	9.11E-01	9.43E-01	1.06E+00	1.18E+00	9.14E-01	8.53E-01	9.92E-01	1.06E+00	1.05E+00	1.01E+00	1.12E+00	1.16E+00
		NFEs	6.52E+04	6.50E+04	6.49E+04	6.49E+04	6.53E+04	6.49E+04	6.49E+04	6.49E+04	6.50E+04	6.51E+04	6.51E+04	6.49E+04
	IP-SP	Min	3.37E+02	4.00E+02	6.00E+02	8.01E+02	9.00E+02	1.84E+03	2.00E+03	2.20E+03	2.53E+03	2.40E+03	2.60E+03	2.86E+03
		Max	1.64E+03	4.93E+02	6.01E+02	8.27E+02	9.38E+02	6.83E+03	2.02E+03	2.22E+03	2.54E+03	2.63E+03	3.00E+03	2.87E+03
		Mean	6.68E+02	4.11E+02	6.00E+02	8.12E+02	9.11E+02	2.99E+03	2.01E+03	2.22E+03	2.53E+03	2.48E+03	2.71E+03	2.87E+03
		STD	4.42E+02	2.15E+01	1.11E-01	1.46E+01	1.48E+01	1.70E+03	1.43E+01	1.96E+01	2.32E+02	1.27E+02	1.60E+02	1.68E+02
		ET	8.99E-01	8.70E-01	1.01E+00	9.15E-01	9.33E-01	8.84E-01	1.06E+00	1.12E+00	1.06E+00	1.02E+00	1.15E+00	1.16E+00
		NFEs	6.52E+04	6.50E+04	6.51E+04	6.53E+04	6.51E+04	6.49E+04	6.52E+04	6.53E+04	6.50E+04	6.53E+04	6.51E+04	6.50E+04

Table 7 continued from previous page

			F_1	F_2	F_3	F_4	F_5	F_6	F_7	F_8	F_9	F_{10}	F_{11}	F_{12}
COOBL-HS	IP	Min	3.78E+02	4.00E+02	6.00E+02	8.02E+02	9.00E+02	1.83E+03	2.00E+03	2.21E+03	2.53E+03	2.40E+03	2.60E+03	2.86E+03
		Max	1.46E+03	4.77E+02	6.00E+02	8.33E+02	9.39E+02	8.07E+03	2.02E+03	2.22E+03	2.54E+03	2.62E+03	3.00E+03	2.87E+03
		Mean	7.49E+02	4.15E+02	6.00E+02	8.14E+02	9.09E+02	3.72E+03	2.02E+03	2.22E+03	2.53E+03	2.55E+03	2.76E+03	2.87E+03
		STD	5.34E+02	2.40E+01	2.80E-02	1.74E+01	1.17E+01	2.65E+03	1.79E+01	1.99E+01	2.32E+02	1.65E+02	1.99E+02	1.65E+02
		ET	8.02E-01	7.98E-01	8.74E-01	8.26E-01	8.32E-01	8.03E-01	9.33E-01	9.80E-01	9.26E-01	9.00E-01	9.94E-01	1.01E+00
		NFEs	5.01E+04	5.01E+04	5.01E+04	5.01E+04	5.01E+04	5.01E+04	5.01E+04	5.01E+04	5.01E+04	5.01E+04	5.01E+04	5.01E+04
	SP	Min	3.00E+02	4.00E+02	6.00E+02	8.04E+02	9.00E+02	1.83E+03	2.00E+03	2.20E+03	2.50E+03	2.50E+03	2.60E+03	2.85E+03
		Max	8.32E+02	4.78E+02	6.00E+02	8.25E+02	1.69E+03	8.24E+06	2.02E+03	2.22E+03	2.54E+03	2.63E+03	3.00E+03	2.87E+03
		Mean	3.25E+02	4.12E+02	6.00E+02	8.13E+02	9.43E+02	7.38E+05	2.02E+03	2.22E+03	2.52E+03	2.55E+03	2.69E+03	2.86E+03
		STD	1.03E+02	2.15E+01	1.68E-03	1.44E+01	1.48E+02	2.07E+06	1.83E+01	1.92E+01	2.24E+02	1.58E+02	1.50E+02	1.63E+02
		ET	9.27E-01	9.05E-01	1.02E+00	9.41E-01	9.53E-01	8.98E-01	1.06E+00	1.13E+00	1.06E+00	1.04E+00	1.17E+00	1.18E+00
		NFEs	6.50E+04	6.51E+04	6.52E+04	6.49E+04	6.51E+04	6.51E+04	6.52E+04	6.50E+04	6.49E+04	6.50E+04	6.52E+04	6.50E+04
	IP-SP	Min	3.00E+02	4.00E+02	6.00E+02	8.02E+02	9.00E+02	1.92E+03	2.00E+03	2.21E+03	2.50E+03	2.40E+03	2.60E+03	2.86E+03
		Max	8.34E+02	4.11E+02	6.00E+02	8.38E+02	1.37E+03	3.59E+06	2.02E+03	2.22E+03	2.55E+03	2.62E+03	3.00E+03	2.90E+03
		Mean	3.30E+02	4.08E+02	6.00E+02	8.16E+02	9.29E+02	2.16E+05	2.02E+03	2.22E+03	2.52E+03	2.54E+03	2.71E+03	2.87E+03
		STD	1.04E+02	7.91E+00	2.78E-03	1.74E+01	8.81E+01	7.68E+05	1.86E+01	1.97E+01	2.20E+02	1.51E+02	1.63E+02	1.69E+02
		ET	8.99E-01	8.72E-01	9.97E-01	9.19E-01	9.36E-01	8.95E-01	1.04E+00	1.11E+00	1.04E+00	1.03E+00	1.13E+00	1.16E+00
		NFEs	6.48E+04	6.49E+04	6.50E+04	6.50E+04	6.51E+04	6.49E+04	6.51E+04	6.52E+04	6.51E+04	6.50E+04	6.49E+04	6.50E+04

Table 7 continued from previous page

			F_1	F_2	F_3	F_4	F_5	F_6	F_7	F_8	F_9	F_{10}	F_{11}	F_{12}
GOBL-HS	IP	Min	3.78E+02	4.00E+02	6.00E+02	8.03E+02	9.00E+02	1.80E+03	2.00E+03	2.20E+03	2.53E+03	2.40E+03	2.60E+03	2.86E+03
		Max	2.07E+03	4.70E+02	6.00E+02	8.29E+02	9.31E+02	5.37E+03	2.02E+03	2.22E+03	2.54E+03	2.63E+03	2.90E+03	2.87E+03
		Mean	7.09E+02	4.10E+02	6.00E+02	8.13E+02	9.07E+02	2.75E+03	2.01E+03	2.22E+03	2.53E+03	2.54E+03	2.69E+03	2.87E+03
		STD	5.36E+02	1.87E+01	7.18E-02	1.63E+01	1.09E+01	1.43E+03	1.46E+01	1.96E+01	2.32E+02	1.63E+02	1.23E+02	1.67E+02
		ET	7.35E-01	7.55E-01	1.58E+00	1.04E+00	1.04E+00	1.01E+00	1.16E+00	1.22E+00	4.82E+00	7.41E+00	7.81E+00	5.47E+00
		NFEs	5.01E+04	5.01E+04	5.01E+04	5.01E+04	5.01E+04	5.01E+04	5.01E+04	5.01E+04	5.01E+04	5.01E+04	5.01E+04	5.01E+04
	SP	Min	3.33E+02	4.00E+02	6.00E+02	8.03E+02	9.00E+02	1.80E+03	2.00E+03	2.21E+03	2.53E+03	2.50E+03	2.60E+03	2.86E+03
		Max	1.40E+03	4.71E+02	6.01E+02	8.32E+02	9.64E+02	5.72E+03	2.02E+03	2.22E+03	2.54E+03	2.62E+03	2.75E+03	2.87E+03
		Mean	6.29E+02	4.10E+02	6.00E+02	8.12E+02	9.08E+02	2.73E+03	2.01E+03	2.22E+03	2.53E+03	2.56E+03	2.67E+03	2.87E+03
		STD	4.15E+02	1.93E+01	1.82E-01	1.50E+01	1.41E+01	1.35E+03	1.59E+01	1.98E+01	2.32E+02	1.66E+02	1.03E+02	1.67E+02
		ET	7.95E-01	7.78E-01	9.15E-01	8.35E-01	8.95E-01	9.36E-01	9.39E-01	9.98E-01	9.32E-01	9.08E-01	1.04E+00	1.04E+00
		NFEs	6.51E+04	6.50E+04	6.49E+04	6.51E+04	6.51E+04	6.50E+04	6.51E+04	6.52E+04	6.52E+04	6.49E+04	6.49E+04	6.51E+04
	IP-SP	Min	3.39E+02	4.00E+02	6.00E+02	8.01E+02	9.00E+02	1.81E+03	2.00E+03	2.20E+03	2.53E+03	2.50E+03	2.60E+03	2.86E+03
		Max	2.14E+03	4.66E+02	6.00E+02	8.31E+02	9.19E+02	6.42E+03	2.02E+03	2.22E+03	2.54E+03	2.62E+03	2.90E+03	2.87E+03
		Mean	6.22E+02	4.09E+02	6.00E+02	8.14E+02	9.06E+02	2.56E+03	2.01E+03	2.22E+03	2.53E+03	2.54E+03	2.67E+03	2.87E+03
		STD	4.80E+02	1.73E+01	5.95E-02	1.71E+01	8.50E+00	1.29E+03	1.55E+01	1.93E+01	2.32E+02	1.52E+02	1.06E+02	1.67E+02
		ET	8.16E-01	8.12E-01	9.19E-01	8.40E-01	8.52E-01	8.18E-01	9.72E-01	1.02E+00	9.44E-01	9.26E-01	1.05E+00	1.06E+00
		NFEs	6.51E+04	6.52E+04	6.50E+04	6.51E+04	6.51E+04	6.50E+04	6.51E+04	6.51E+04	6.52E+04	6.50E+04	6.51E+04	6.53E+04

Table 7 continued from previous page

			F_1	F_2	F_3	F_4	F_5	F_6	F_7	F_8	F_9	F_{10}	F_{11}	F_{12}
QOBL-HS	IP	Min	3.50E+02	4.00E+02	6.00E+02	8.02E+02	9.00E+02	1.82E+03	2.00E+03	2.21E+03	2.53E+03	2.50E+03	2.60E+03	2.86E+03
		Max	1.23E+03	4.12E+02	6.00E+02	8.32E+02	9.12E+02	5.60E+03	2.02E+03	2.22E+03	2.54E+03	2.62E+03	2.90E+03	2.87E+03
		Mean	6.12E+02	4.04E+02	6.00E+02	8.16E+02	9.05E+02	2.60E+03	2.01E+03	2.22E+03	2.53E+03	2.58E+03	2.66E+03	2.87E+03
		STD	3.97E+02	5.63E+00	7.75E-02	1.92E+01	6.10E+00	1.24E+03	1.67E+01	1.75E+01	2.32E+02	1.83E+02	9.90E+01	1.67E+02
		ET	8.57E-01	8.42E-01	9.34E-01	8.60E-01	8.81E-01	8.39E-01	9.99E-01	1.06E+00	1.02E+00	9.50E-01	1.07E+00	1.06E+00
		FES	5.01E+04	5.01E+04	5.01E+04	5.01E+04	5.01E+04	5.01E+04	5.01E+04	5.01E+04	5.01E+04	5.01E+04	5.01E+04	5.01E+04
	SP	Min	3.83E+02	4.00E+02	6.00E+02	8.07E+02	9.00E+02	1.82E+03	2.00E+03	2.21E+03	2.53E+03	2.50E+03	2.60E+03	2.86E+03
		Max	1.76E+03	4.64E+02	6.00E+02	8.34E+02	9.12E+02	5.19E+03	2.02E+03	2.22E+03	2.54E+03	2.61E+03	2.90E+03	2.88E+03
		Mean	7.60E+02	4.06E+02	6.00E+02	8.27E+02	9.03E+02	2.61E+03	2.01E+03	2.22E+03	2.53E+03	2.57E+03	2.67E+03	2.87E+03
		STD	5.71E+02	1.30E+01	3.26E-02	2.75E+01	3.94E+00	1.18E+03	1.41E+01	1.93E+01	2.32E+02	1.79E+02	1.34E+02	1.68E+02
		ET	1.29E+00	1.26E+00	1.42E+00	1.31E+00	1.31E+00	1.29E+00	1.49E+00	1.54E+00	1.47E+00	1.43E+00	1.58E+00	1.60E+00
		NFEs	6.52E+04	6.53E+04	6.51E+04	6.48E+04	6.51E+04	6.52E+04	6.52E+04	6.50E+04	6.52E+04	6.52E+04	6.51E+04	6.49E+04
	IP-SP	Min	3.46E+02	4.00E+02	6.00E+02	8.03E+02	9.00E+02	1.81E+03	2.00E+03	2.20E+03	2.53E+03	2.54E+03	2.60E+03	2.86E+03
		Max	1.43E+03	4.71E+02	6.00E+02	8.30E+02	9.40E+02	7.70E+03	2.02E+03	2.22E+03	2.55E+03	2.62E+03	2.90E+03	2.87E+03
		Mean	5.91E+02	4.14E+02	6.00E+02	8.15E+02	9.06E+02	3.17E+03	2.01E+03	2.22E+03	2.53E+03	2.60E+03	2.69E+03	2.87E+03
		STD	3.72E+02	2.74E+01	6.77E-02	1.79E+01	1.03E+01	2.07E+03	1.55E+01	1.87E+01	2.33E+02	1.99E+02	1.37E+02	1.68E+02
		ET	1.46E+00	1.47E+00	1.60E+00	1.37E+00	1.40E+00	1.34E+00	2.87E+00	9.56E-01	8.86E-01	9.47E-01	1.04E+00	1.02E+00
		NFEs	6.51E+04	6.52E+04	6.50E+04	6.50E+04	6.51E+04	6.53E+04	6.51E+04	6.52E+04	6.49E+04	6.52E+04	6.50E+04	6.52E+04

Table 7 continued from previous page

			F_1	F_2	F_3	F_4	F_5	F_6	F_7	F_8	F_9	F_{10}	F_{11}	F_{12}
QROBL-HS	IP	Min	3.09E+02	4.00E+02	6.00E+02	8.03E+02	9.00E+02	1.80E+03	2.00E+03	2.21E+03	2.53E+03	2.50E+03	2.60E+03	2.86E+03
		Max	1.17E+03	4.72E+02	6.00E+02	8.31E+02	9.30E+02	7.44E+03	2.02E+03	2.22E+03	2.54E+03	2.62E+03	2.90E+03	2.87E+03
		Mean	5.85E+02	4.08E+02	6.00E+02	8.17E+02	9.09E+02	3.27E+03	2.01E+03	2.22E+03	2.53E+03	2.58E+03	2.70E+03	2.87E+03
		STD	3.55E+02	1.89E+01	6.96E-02	1.93E+01	1.10E+01	2.01E+03	1.42E+01	1.89E+01	2.32E+02	1.84E+02	1.55E+02	1.68E+02
		ET	5.65E-01	5.34E-01	6.05E-01	5.62E-01	5.70E-01	5.48E-01	6.47E-01	6.75E-01	6.30E-01	6.18E-01	9.00E-01	1.07E+00
		NFEs	5.01E+04	5.01E+04	5.01E+04	5.01E+04	5.01E+04	5.01E+04	5.01E+04	5.01E+04	5.01E+04	5.01E+04	5.01E+04	5.01E+04
	SP	Min	3.30E+02	4.00E+02	6.00E+02	8.12E+02	9.00E+02	1.81E+03	2.00E+03	2.21E+03	2.53E+03	2.40E+03	2.60E+03	2.86E+03
		Max	1.53E+03	4.75E+02	6.00E+02	8.36E+02	9.25E+02	4.12E+03	2.02E+03	2.22E+03	2.54E+03	2.61E+03	2.90E+03	2.87E+03
		Mean	6.50E+02	4.07E+02	6.00E+02	8.27E+02	9.04E+02	2.51E+03	2.01E+03	2.22E+03	2.53E+03	2.56E+03	2.66E+03	2.87E+03
		STD	4.58E+02	1.48E+01	6.34E-02	2.72E+01	6.09E+00	9.73E+02	1.29E+01	1.94E+01	2.31E+02	1.71E+02	1.13E+02	1.68E+02
		ET	1.12E+00	1.06E+00	1.29E+00	1.14E+00	1.17E+00	1.09E+00	1.29E+00	1.27E+00	1.22E+00	1.15E+00	1.40E+00	1.10E+00
		NFEs	6.51E+04	6.50E+04	6.49E+04	6.50E+04	6.52E+04	6.51E+04	6.50E+04	6.53E+04	6.51E+04	6.49E+04	6.49E+04	6.52E+04
	IP-SP	Min	3.64E+02	4.00E+02	6.00E+02	8.05E+02	9.00E+02	1.81E+03	2.00E+03	2.21E+03	2.53E+03	2.50E+03	2.60E+03	2.86E+03
		Max	1.62E+03	4.71E+02	6.00E+02	8.35E+02	9.14E+02	5.02E+03	2.02E+03	2.22E+03	2.54E+03	2.61E+03	2.75E+03	2.87E+03
		Mean	7.02E+02	4.06E+02	6.00E+02	8.26E+02	9.03E+02	2.80E+03	2.01E+03	2.22E+03	2.53E+03	2.57E+03	2.64E+03	2.87E+03
		STD	4.82E+02	1.82E+01	4.53E-02	2.68E+01	4.62E+00	1.32E+03	1.42E+01	1.94E+01	2.32E+02	1.79E+02	7.28E+01	1.68E+02
		ET	1.33E+00	1.21E+00	1.27E+00	1.35E+00	1.17E+00	1.18E+00	1.47E+00	1.58E+00	1.28E+00	1.20E+00	1.46E+00	1.16E+00
		NFEs	6.51E+04	6.53E+04	6.51E+04	6.52E+04	6.52E+04	6.51E+04	6.50E+04	6.52E+04	6.50E+04	6.51E+04	6.52E+04	6.53E+04

Table 8: Performance metrics for HS variants across 12 functions ($D = 20$)

			F_1	F_2	F_3	F_4	F_5	F_6	F_7	F_8	F_9	F_{10}	F_{11}	F_{12}
HS		Min	6.43E+03	4.45E+02	6.00E+02	8.78E+02	9.04E+02	1.85E+03	2.02E+03	2.22E+03	2.48E+03	2.40E+03	2.90E+03	2.93E+03
		Max	1.96E+04	4.52E+02	6.01E+02	9.20E+02	9.48E+02	1.13E+04	2.07E+03	2.24E+03	2.49E+03	2.50E+03	2.93E+03	2.96E+03
		Mean	1.18E+04	4.50E+02	6.00E+02	9.02E+02	9.15E+02	3.87E+03	2.03E+03	2.22E+03	2.49E+03	2.41E+03	2.91E+03	2.95E+03
		STD	1.19E+04	4.99E+01	5.03E-01	1.02E+02	1.76E+01	3.11E+03	3.70E+01	2.47E+01	1.86E+02	3.68E+01	3.12E+02	2.50E+02
		ET	8.77E-01	8.52E-01	1.01E+00	9.01E-01	9.26E-01	8.89E-01	1.09E+00	1.15E+00	1.14E+00	1.06E+00	1.20E+00	1.24E+00
		NFEs	5.00E+04	5.00E+04	5.00E+04	5.00E+04	5.00E+04	5.00E+04	5.00E+04	5.00E+04	5.00E+04	5.00E+04	5.00E+04	5.00E+04
BOBL-HS	IP	Min	6.16E+03	4.49E+02	6.00E+02	8.86E+02	9.02E+02	1.94E+03	2.01E+03	2.22E+03	2.48E+03	2.40E+03	2.91E+03	2.95E+03
		Max	1.54E+04	4.83E+02	6.02E+02	9.14E+02	9.38E+02	1.53E+04	2.09E+03	2.24E+03	2.49E+03	2.50E+03	3.06E+03	2.98E+03
		Mean	9.96E+03	4.54E+02	6.00E+02	9.01E+02	9.16E+02	5.84E+03	2.04E+03	2.22E+03	2.49E+03	2.40E+03	2.93E+03	2.96E+03
		STD	9.98E+03	5.47E+01	5.40E-01	1.01E+02	1.81E+01	5.50E+03	4.80E+01	2.48E+01	1.86E+02	1.84E+01	3.31E+02	2.61E+02
		ET	9.58E-01	9.82E-01	1.53E+01	1.81E+00	1.74E+00	1.67E+00	1.59E+00	3.23E+00	9.40E+00	1.13E+01	8.36E+00	1.76E+00
		NFEs	5.01E+04	5.01E+04	5.01E+04	5.01E+04	5.01E+04	5.01E+04	5.01E+04	5.01E+04	5.01E+04	5.01E+04	5.01E+04	5.01E+04
	SP	Min	6.63E+03	4.45E+02	6.00E+02	8.82E+02	9.01E+02	1.86E+03	2.02E+03	2.22E+03	2.48E+03	2.40E+03	2.91E+03	2.94E+03
		Max	1.76E+04	4.84E+02	6.01E+02	9.15E+02	9.46E+02	1.81E+04	2.09E+03	2.24E+03	2.49E+03	2.50E+03	2.94E+03	2.97E+03
		Mean	1.03E+04	4.56E+02	6.01E+02	9.01E+02	9.19E+02	4.79E+03	2.04E+03	2.22E+03	2.49E+03	2.41E+03	2.93E+03	2.95E+03
		STD	1.05E+04	5.77E+01	5.74E-01	1.01E+02	2.17E+01	4.50E+03	4.71E+01	2.45E+01	1.85E+02	2.60E+01	3.26E+02	2.50E+02
		ET	1.27E+00	1.27E+00	1.54E+00	1.36E+00	1.36E+00	1.31E+00	1.60E+00	1.68E+00	1.65E+00	1.52E+00	1.82E+00	1.85E+00
		NFEs	6.50E+04	6.51E+04	6.48E+04	6.50E+04	6.50E+04	6.50E+04	6.50E+04	6.52E+04	6.52E+04	6.48E+04	6.52E+04	6.50E+04
	IP-SP	Min	6.21E+03	4.45E+02	6.00E+02	8.75E+02	9.03E+02	1.82E+03	2.02E+03	2.22E+03	2.48E+03	2.40E+03	2.91E+03	2.94E+03
		Max	1.82E+04	4.81E+02	6.02E+02	9.16E+02	9.25E+02	1.51E+04	2.07E+03	2.25E+03	2.49E+03	2.40E+03	2.95E+03	2.98E+03
		Mean	1.06E+04	4.55E+02	6.01E+02	9.00E+02	9.14E+02	4.76E+03	2.04E+03	2.23E+03	2.49E+03	2.40E+03	2.93E+03	2.96E+03
		STD	1.06E+04	5.60E+01	6.60E-01	1.01E+02	1.51E+01	4.54E+03	4.01E+01	2.64E+01	1.85E+02	9.14E-01	3.26E+02	2.59E+02
		ET	1.30E+00	1.31E+00	1.57E+00	1.35E+00	1.39E+00	1.32E+00	1.62E+00	1.71E+00	1.65E+00	1.57E+00	1.82E+00	1.94E+00
		NFEs	6.51E+04	6.49E+04	6.52E+04	6.50E+04	6.50E+04	6.52E+04	6.51E+04	6.51E+04	6.52E+04	6.52E+04	6.49E+04	6.52E+04

Table 8 continued from previous page

			F_1	F_2	F_3	F_4	F_5	F_6	F_7	F_8	F_9	F_{10}	F_{11}	F_{12}
COOBL-HS	IP	Min	7.59E+03	4.45E+02	6.00E+02	8.75E+02	9.03E+02	1.88E+03	2.02E+03	2.22E+03	2.48E+03	2.40E+03	2.91E+03	2.94E+03
		Max	2.17E+04	4.85E+02	6.01E+02	9.16E+02	9.37E+02	1.74E+04	2.09E+03	2.24E+03	2.50E+03	2.50E+03	3.07E+03	2.98E+03
		Mean	1.28E+04	4.52E+02	6.00E+02	8.98E+02	9.16E+02	5.35E+03	2.04E+03	2.23E+03	2.48E+03	2.40E+03	2.93E+03	2.96E+03
		STD	1.30E+04	5.31E+01	5.24E-01	9.83E+01	1.81E+01	5.27E+03	4.77E+01	2.69E+01	1.85E+02	1.84E+01	3.29E+02	2.56E+02
		ET	1.28E+00	1.19E+00	1.39E+00	1.25E+00	1.25E+00	1.27E+00	1.47E+00	1.51E+00	1.49E+00	1.41E+00	1.59E+00	1.65E+00
		NFEs	5.01E+04	5.01E+04	5.01E+04	5.01E+04	5.01E+04	5.01E+04	5.01E+04	5.01E+04	5.01E+04	5.01E+04	5.01E+04	5.01E+04
	SP	Min	3.63E+02	4.47E+02	6.00E+02	8.17E+02	9.46E+02	1.90E+03	2.02E+03	2.22E+03	2.48E+03	2.40E+03	2.90E+03	2.90E+03
		Max	6.00E+03	5.35E+02	6.01E+02	8.59E+02	1.47E+03	3.06E+08	2.09E+03	2.24E+03	2.49E+03	2.63E+03	2.90E+03	2.97E+03
		Mean	2.04E+03	4.64E+02	6.00E+02	8.38E+02	1.08E+03	1.07E+07	2.05E+03	2.22E+03	2.48E+03	2.43E+03	2.90E+03	2.90E+03
		STD	2.40E+03	6.77E+01	4.98E-01	3.90E+01	2.21E+02	5.59E+07	4.91E+01	2.46E+01	1.83E+02	6.67E+01	3.00E+02	2.03E+02
		ET	1.31E+00	1.28E+00	1.58E+00	1.38E+00	1.41E+00	1.33E+00	1.66E+00	1.72E+00	1.69E+00	1.58E+00	1.86E+00	1.91E+00
		NFEs	6.47E+04	6.50E+04	6.52E+04	6.51E+04	6.52E+04	6.52E+04	6.50E+04	6.52E+04	6.51E+04	6.50E+04	6.50E+04	6.53E+04
	IP-SP	Min	4.19E+02	4.45E+02	6.00E+02	8.25E+02	9.21E+02	1.91E+03	2.02E+03	2.22E+03	2.48E+03	2.40E+03	2.90E+03	2.90E+03
		Max	7.35E+03	5.17E+02	6.01E+02	8.81E+02	1.35E+03	1.31E+07	2.13E+03	2.24E+03	2.49E+03	2.50E+03	3.00E+03	2.90E+03
		Mean	1.89E+03	4.60E+02	6.00E+02	8.46E+02	1.06E+03	7.17E+05	2.05E+03	2.22E+03	2.48E+03	2.42E+03	2.90E+03	2.90E+03
		STD	2.18E+03	6.24E+01	5.77E-01	4.84E+01	1.91E+02	2.71E+06	5.12E+01	2.46E+01	1.82E+02	4.51E+01	3.04E+02	2.00E+02
		ET	1.27E+00	1.26E+00	1.53E+00	1.55E+00	1.54E+00	1.32E+00	1.62E+00	1.67E+00	1.67E+00	1.56E+00	1.82E+00	1.89E+00
		NFEs	6.50E+04	6.51E+04	6.51E+04	6.52E+04	6.52E+04	6.52E+04	6.51E+04	6.51E+04	6.52E+04	6.53E+04	6.49E+04	6.50E+04

Table 8 continued from previous page

			F_1	F_2	F_3	F_4	F_5	F_6	F_7	F_8	F_9	F_{10}	F_{11}	F_{12}
GOBL-HS	IP	Min	6.36E+03	4.49E+02	6.00E+02	8.75E+02	9.02E+02	1.86E+03	2.02E+03	2.22E+03	2.48E+03	2.40E+03	2.91E+03	2.94E+03
		Max	2.13E+04	4.99E+02	6.01E+02	9.14E+02	9.51E+02	9.46E+03	2.07E+03	2.24E+03	2.49E+03	2.50E+03	2.95E+03	2.97E+03
		Mean	1.18E+04	4.53E+02	6.00E+02	9.00E+02	9.21E+02	4.00E+03	2.04E+03	2.22E+03	2.48E+03	2.41E+03	2.93E+03	2.95E+03
		STD	1.20E+04	5.35E+01	5.80E-01	1.00E+02	2.40E+01	3.04E+03	4.24E+01	2.47E+01	1.84E+02	3.19E+01	3.27E+02	2.53E+02
		ET	1.00E+01	8.88E+00	5.20E+00	1.15E+00	1.16E+00	1.12E+00	1.39E+00	1.40E+00	1.36E+00	1.29E+00	1.47E+00	1.50E+00
		NFEs	5.01E+04	5.01E+04	5.01E+04	5.01E+04	5.01E+04	5.01E+04	5.01E+04	5.01E+04	5.01E+04	5.01E+04	5.01E+04	5.01E+04
	SP	Min	3.71E+03	4.46E+02	6.00E+02	8.81E+02	9.02E+02	1.83E+03	2.02E+03	2.22E+03	2.48E+03	2.40E+03	2.91E+03	2.94E+03
		Max	2.35E+04	4.79E+02	6.01E+02	9.18E+02	9.39E+02	7.66E+03	2.07E+03	2.25E+03	2.49E+03	2.50E+03	2.94E+03	2.97E+03
		Mean	1.13E+04	4.54E+02	6.01E+02	9.02E+02	9.17E+02	3.72E+03	2.04E+03	2.22E+03	2.49E+03	2.41E+03	2.93E+03	2.95E+03
		STD	1.17E+04	5.52E+01	5.94E-01	1.02E+02	2.00E+01	2.54E+03	4.15E+01	2.56E+01	1.85E+02	2.60E+01	3.29E+02	2.54E+02
		ET	1.17E+00	1.17E+00	1.43E+00	1.24E+00	1.25E+00	1.19E+00	1.49E+00	1.55E+00	1.51E+00	1.41E+00	1.65E+00	1.71E+00
		NFEs	6.51E+04	6.50E+04	6.49E+04	6.51E+04	6.51E+04	6.50E+04	6.51E+04	6.52E+04	6.52E+04	6.49E+04	6.49E+04	6.51E+04
	IP-SP	Min	4.56E+03	4.37E+02	6.00E+02	8.83E+02	9.04E+02	1.84E+03	2.02E+03	2.22E+03	2.48E+03	2.40E+03	2.90E+03	2.94E+03
		Max	2.07E+04	4.94E+02	6.01E+02	9.11E+02	9.63E+02	8.72E+03	2.07E+03	2.25E+03	2.49E+03	2.50E+03	3.01E+03	2.97E+03
		Mean	1.19E+04	4.54E+02	6.00E+02	9.02E+02	9.16E+02	3.32E+03	2.04E+03	2.22E+03	2.48E+03	2.40E+03	2.93E+03	2.95E+03
		STD	1.21E+04	5.49E+01	5.02E-01	1.03E+02	2.03E+01	2.29E+03	3.92E+01	2.55E+01	1.85E+02	1.85E+01	3.31E+02	2.52E+02
		ET	1.19E+00	1.20E+00	1.44E+00	1.26E+00	1.27E+00	1.20E+00	1.52E+00	1.57E+00	1.53E+00	1.44E+00	1.67E+00	1.74E+00
		NFEs	6.50E+04	6.53E+04	6.50E+04	6.51E+04	6.55E+04	6.52E+04	6.52E+04	6.50E+04	6.50E+04	6.50E+04	6.48E+04	6.51E+04

Table 8 continued from previous page

			F_1	F_2	F_3	F_4	F_5	F_6	F_7	F_8	F_9	F_{10}	F_{11}	F_{12}
QOBL-HS	IP	Min	4.53E+03	4.46E+02	6.00E+02	8.79E+02	9.03E+02	1.86E+03	2.02E+03	2.22E+03	2.48E+03	2.40E+03	2.90E+03	2.94E+03
		Max	1.61E+04	4.81E+02	6.01E+02	9.12E+02	9.52E+02	7.92E+03	2.09E+03	2.25E+03	2.49E+03	2.50E+03	2.94E+03	2.97E+03
		Mean	9.69E+03	4.58E+02	6.00E+02	9.02E+02	9.15E+02	3.42E+03	2.04E+03	2.22E+03	2.49E+03	2.41E+03	2.92E+03	2.95E+03
		STD	9.80E+03	5.94E+01	4.38E-01	1.03E+02	1.71E+01	2.06E+03	4.60E+01	2.47E+01	1.86E+02	3.68E+01	3.25E+02	2.54E+02
		ET	1.24E+00	1.25E+00	1.49E+00	1.33E+00	1.35E+00	1.30E+00	1.64E+00	1.85E+00	1.79E+00	1.71E+00	1.97E+00	2.05E+00
		NFEs	5.01E+04	5.01E+04	5.01E+04	5.01E+04	5.01E+04	5.01E+04	5.01E+04	5.01E+04	5.01E+04	5.01E+04	5.01E+04	5.01E+04
	SP	Min	4.21E+03	4.50E+02	6.00E+02	8.87E+02	9.02E+02	1.94E+03	2.02E+03	2.22E+03	2.48E+03	2.40E+03	2.91E+03	2.94E+03
		Max	1.21E+04	4.74E+02	6.01E+02	9.25E+02	9.24E+02	5.65E+03	2.06E+03	2.24E+03	2.49E+03	2.50E+03	3.02E+03	2.97E+03
		Mean	8.01E+03	4.57E+02	6.00E+02	9.09E+02	9.12E+02	3.46E+03	2.04E+03	2.23E+03	2.49E+03	2.42E+03	2.93E+03	2.95E+03
		STD	7.97E+03	5.79E+01	4.72E-01	1.10E+02	1.28E+01	2.00E+03	4.41E+01	2.58E+01	1.86E+02	4.51E+01	3.35E+02	2.52E+02
		ET	1.99E+00	2.03E+00	2.29E+00	2.07E+00	2.06E+00	2.03E+00	2.41E+00	2.53E+00	2.50E+00	2.34E+00	2.64E+00	3.00E+00
		NFEs	6.52E+04	6.51E+04	6.50E+04	6.50E+04	6.50E+04	6.53E+04	6.49E+04	6.49E+04	6.51E+04	6.51E+04	6.51E+04	6.50E+04
	IP-SP	Min	5.35E+03	4.49E+02	6.00E+02	8.83E+02	9.03E+02	1.89E+03	2.02E+03	2.22E+03	2.48E+03	2.40E+03	2.92E+03	2.94E+03
		Max	1.62E+04	4.69E+02	6.01E+02	9.13E+02	9.41E+02	7.22E+03	2.06E+03	2.24E+03	2.49E+03	2.50E+03	3.05E+03	2.98E+03
		Mean	9.77E+03	4.53E+02	6.00E+02	9.03E+02	9.14E+02	3.42E+03	2.04E+03	2.22E+03	2.48E+03	2.42E+03	2.94E+03	2.95E+03
		STD	9.81E+03	5.28E+01	4.78E-01	1.03E+02	1.71E+01	2.13E+03	4.48E+01	2.49E+01	1.84E+02	4.11E+01	3.45E+02	2.54E+02
		ET	1.30E+00	1.29E+00	1.53E+00	1.36E+00	1.35E+00	1.31E+00	1.56E+00	1.60E+00	1.57E+00	1.48E+00	1.67E+00	1.71E+00
		NFEs	6.53E+04	6.50E+04	6.50E+04	6.50E+04	6.51E+04	6.52E+04	6.50E+04	6.51E+04	6.54E+04	6.52E+04	6.52E+04	6.51E+04

Table 8 continued from previous page

			F_1	F_2	F_3	F_4	F_5	F_6	F_7	F_8	F_9	F_{10}	F_{11}	F_{12}
QROBL-HS	IP	Min	5.43E+03	4.49E+02	6.00E+02	8.78E+02	9.03E+02	1.98E+03	2.02E+03	2.22E+03	2.48E+03	2.40E+03	2.91E+03	2.94E+03
		Max	1.40E+04	4.76E+02	6.01E+02	9.17E+02	9.46E+02	8.68E+03	2.07E+03	2.25E+03	2.49E+03	2.50E+03	3.01E+03	2.97E+03
		Mean	9.59E+03	4.55E+02	6.00E+02	9.03E+02	9.16E+02	3.82E+03	2.04E+03	2.22E+03	2.48E+03	2.41E+03	2.93E+03	2.95E+03
		STD	9.52E+03	5.53E+01	5.85E-01	1.03E+02	1.78E+01	2.50E+03	4.23E+01	2.42E+01	1.84E+02	3.19E+01	3.30E+02	2.54E+02
		ET	1.49E+00	1.33E+00	1.40E+00	1.38E+00	1.22E+00	1.36E+00	1.45E+00	1.60E+00	1.51E+00	1.43E+00	1.56E+00	1.61E+00
		NFEs	5.01E+04	5.01E+04	5.01E+04	5.01E+04	5.01E+04	5.01E+04	5.01E+04	5.01E+04	5.01E+04	5.01E+04	5.01E+04	5.01E+04
	SP	Min	4.73E+03	4.50E+02	6.00E+02	8.96E+02	9.02E+02	2.11E+03	2.02E+03	2.22E+03	2.48E+03	2.40E+03	2.91E+03	2.94E+03
		Max	1.24E+04	4.76E+02	6.01E+02	9.23E+02	9.32E+02	6.19E+03	2.05E+03	2.24E+03	2.49E+03	2.50E+03	2.94E+03	2.97E+03
		Mean	8.09E+03	4.59E+02	6.00E+02	9.08E+02	9.13E+02	3.48E+03	2.04E+03	2.22E+03	2.49E+03	2.41E+03	2.93E+03	2.95E+03
		STD	8.08E+03	5.95E+01	4.59E-01	1.09E+02	1.52E+01	2.05E+03	4.19E+01	2.53E+01	1.86E+02	3.18E+01	3.25E+02	2.51E+02
		ET	1.35E+00	1.37E+00	1.55E+00	1.42E+00	1.62E+00	1.38E+00	1.67E+00	1.62E+00	1.58E+00	1.87E+00	2.02E+00	2.21E+00
		NFEs	6.50E+04	6.51E+04	6.49E+04	6.50E+04	6.52E+04	6.51E+04	6.50E+04	6.51E+04	6.50E+04	6.50E+04	6.52E+04	6.47E+04
	IP-SP	Min	5.01E+03	4.50E+02	6.00E+02	8.89E+02	9.02E+02	2.00E+03	2.03E+03	2.22E+03	2.48E+03	2.40E+03	2.90E+03	2.94E+03
		Max	1.31E+04	4.75E+02	6.01E+02	9.20E+02	9.36E+02	5.51E+03	2.06E+03	2.24E+03	2.50E+03	2.50E+03	3.04E+03	2.97E+03
		Mean	8.38E+03	4.56E+02	6.00E+02	9.06E+02	9.15E+02	3.46E+03	2.04E+03	2.22E+03	2.49E+03	2.41E+03	2.93E+03	2.95E+03
		STD	8.28E+03	5.60E+01	5.52E-01	1.06E+02	1.70E+01	1.86E+03	4.31E+01	2.41E+01	1.86E+02	3.19E+01	3.34E+02	2.51E+02
		ET	1.74E+00	1.84E+00	2.12E+00	2.01E+00	1.96E+00	1.93E+00	2.12E+00	1.90E+00	1.54E+00	1.45E+00	1.61E+00	1.67E+00
		NFEs	6.49E+04	6.51E+04	6.50E+04	6.53E+04	6.48E+04	6.49E+04	6.52E+04	6.52E+04	6.53E+04	6.50E+04	6.50E+04	6.50E+04

Table 9: Performance metrics for ABC variants across 12 functions ($D = 10$)

			F_1	F_2	F_3	F_4	F_5	F_6	F_7	F_8	F_9	F_{10}	F_{11}	F_{12}
ABC		Min	7.43E+03	4.05E+02	6.00E+02	8.22E+02	9.00E+02	2.16E+04	2.02E+03	2.23E+03	2.49E+03	2.50E+03	2.60E+03	2.88E+03
		Max	1.79E+04	4.07E+02	6.00E+02	8.37E+02	9.00E+02	1.01E+06	2.03E+03	2.24E+03	2.49E+03	2.51E+03	2.69E+03	2.90E+03
		Mean	1.30E+04	4.06E+02	6.00E+02	8.30E+02	9.00E+02	3.34E+05	2.03E+03	2.23E+03	2.49E+03	2.50E+03	2.60E+03	2.90E+03
		STD	1.30E+04	5.93E+00	5.06E-04	3.06E+01	5.42E-07	4.36E+05	2.82E+01	3.20E+01	1.89E+02	1.05E+02	1.63E+01	1.98E+02
		ET	8.59E-01	8.51E-01	1.13E+00	8.70E-01	1.30E+00	1.98E+00	2.55E+00	2.77E+00	2.60E+00	2.19E+00	2.57E+00	2.56E+00
		NFEs	1.00E+05	1.00E+05	1.00E+05	1.00E+05	1.00E+05	1.00E+05	1.00E+05	1.00E+05	1.00E+05	1.00E+05	1.00E+05	1.00E+05
BOBL-ABC	IP	Min	5.12E+03	4.05E+02	6.00E+02	8.07E+02	9.00E+02	4.72E+04	2.02E+03	2.23E+03	2.49E+03	2.50E+03	2.60E+03	2.87E+03
		Max	1.91E+04	4.07E+02	6.00E+02	8.41E+02	9.00E+02	5.20E+05	2.03E+03	2.24E+03	2.49E+03	2.51E+03	2.75E+03	2.90E+03
		Mean	1.19E+04	4.06E+02	6.00E+02	8.30E+02	9.00E+02	2.15E+05	2.03E+03	2.23E+03	2.49E+03	2.50E+03	2.61E+03	2.89E+03
		STD	1.22E+04	5.90E+00	6.47E-04	3.09E+01	1.62E-06	2.51E+05	2.81E+01	3.16E+01	1.90E+02	1.03E+02	3.75E+01	1.95E+02
		ET	1.79E+00	1.81E+00	2.20E+00	1.90E+00	1.96E+00	1.78E+00	1.46E+00	1.69E+00	1.32E+00	9.61E-01	1.14E+00	1.13E+00
		NFEs	1.00E+05	1.00E+05	1.00E+05	1.00E+05	1.00E+05	1.00E+05	1.00E+05	1.00E+05	1.00E+05	1.00E+05	1.00E+05	1.00E+05
	SP	Min	9.84E+02	4.05E+02	6.00E+02	8.31E+02	9.00E+02	7.06E+05	2.03E+03	2.23E+03	2.49E+03	2.50E+03	2.60E+03	2.90E+03
		Max	1.03E+04	4.06E+02	6.00E+02	8.47E+02	9.00E+02	6.55E+06	2.03E+03	2.24E+03	2.49E+03	2.50E+03	2.60E+03	2.90E+03
		Mean	3.24E+03	4.06E+02	6.00E+02	8.38E+02	9.00E+02	3.44E+06	2.03E+03	2.24E+03	2.49E+03	2.50E+03	2.60E+03	2.90E+03
		STD	3.45E+03	5.78E+00	3.13E-11	3.85E+01	2.41E-08	3.82E+06	2.84E+01	3.58E+01	1.86E+02	1.00E+02	5.30E-03	2.00E+02
		ET	9.13E-01	8.95E-01	1.11E+00	9.43E-01	9.78E-01	8.83E-01	1.16E+00	1.25E+00	1.12E+00	1.12E+00	1.33E+00	1.31E+00
		NFEs	1.15E+05	1.15E+05	1.15E+05	1.15E+05	1.15E+05	1.16E+05	1.15E+05	1.15E+05	1.15E+05	1.16E+05	1.15E+05	1.16E+05
	IP-SP	Min	8.89E+02	4.05E+02	6.00E+02	8.24E+02	9.00E+02	6.86E+05	2.03E+03	2.23E+03	2.49E+03	2.50E+03	2.60E+03	2.90E+03
		Max	4.42E+03	4.07E+02	6.00E+02	8.47E+02	9.00E+02	8.89E+06	2.03E+03	2.24E+03	2.49E+03	2.50E+03	2.75E+03	2.90E+03
		Mean	2.59E+03	4.06E+02	6.00E+02	8.37E+02	9.00E+02	3.37E+06	2.03E+03	2.23E+03	2.49E+03	2.50E+03	2.61E+03	2.90E+03
		STD	2.45E+03	5.89E+00	1.17E-11	3.79E+01	2.71E-09	3.90E+06	2.86E+01	3.47E+01	1.86E+02	1.00E+02	3.19E+01	2.00E+02
		ET	9.35E-01	9.47E-01	1.37E+00	1.04E+00	1.06E+00	9.47E-01	1.44E+00	1.58E+00	1.54E+00	1.36E+00	1.76E+00	1.85E+00
		NFEs	1.00E+05	1.00E+05	1.00E+05	1.00E+05	1.00E+05	1.00E+05	1.00E+05	1.00E+05	1.00E+05	1.00E+05	1.00E+05	1.00E+05

Table 9 continued from previous page

			F_1	F_2	F_3	F_4	F_5	F_6	F_7	F_8	F_9	F_{10}	F_{11}	F_{12}
COOBL-ABC	IP	Min	9.08E+03	4.05E+02	6.00E+02	8.22E+02	9.00E+02	2.60E+04	2.02E+03	2.23E+03	2.49E+03	2.50E+03	2.60E+03	2.85E+03
		Max	2.21E+04	4.09E+02	6.00E+02	8.38E+02	9.00E+02	9.59E+05	2.03E+03	2.24E+03	2.49E+03	2.53E+03	2.76E+03	2.90E+03
		Mean	1.37E+04	4.06E+02	6.00E+02	8.31E+02	9.00E+02	3.88E+05	2.03E+03	2.23E+03	2.49E+03	2.51E+03	2.61E+03	2.89E+03
		STD	1.39E+04	6.20E+00	6.34E-04	3.12E+01	1.11E-06	4.70E+05	2.84E+01	3.24E+01	1.90E+02	1.06E+02	4.10E+01	1.89E+02
		ET	6.12E-01	6.44E-01	8.22E-01	6.87E-01	7.48E-01	6.63E-01	8.38E-01	9.30E-01	8.52E-01	8.09E-01	9.68E-01	9.35E-01
		NFEs	1.00E+05	1.00E+05	1.00E+05	1.00E+05	1.00E+05	1.00E+05	1.00E+05	1.00E+05	1.00E+05	1.00E+05	1.00E+05	1.00E+05
	SP	Min	3.00E+02	4.00E+02	6.00E+02	8.09E+02	9.00E+02	2.18E+03	2.02E+03	2.20E+03	2.49E+03	2.50E+03	2.60E+03	2.85E+03
		Max	3.18E+04	4.07E+02	6.00E+02	8.60E+02	1.35E+03	7.04E+06	2.06E+03	2.27E+03	2.49E+03	2.59E+03	2.60E+03	2.90E+03
		Mean	5.94E+03	4.01E+02	6.00E+02	8.41E+02	9.25E+02	1.08E+06	2.03E+03	2.24E+03	2.49E+03	2.50E+03	2.60E+03	2.89E+03
		STD	1.21E+04	1.73E+00	1.42E-02	4.45E+01	9.73E+01	2.00E+06	3.21E+01	3.76E+01	1.86E+02	1.05E+02	0.00E+00	1.94E+02
		ET	7.82E-01	7.69E-01	9.09E-01	9.14E-01	8.32E-01	8.76E-01	1.07E+00	1.16E+00	9.82E-01	1.30E+01	1.05E+00	1.17E+00
		NFEs	1.15E+05	1.15E+05	1.15E+05	1.15E+05	1.15E+05	1.15E+05	1.15E+05	1.15E+05	1.15E+05	1.15E+05	1.15E+05	1.15E+05
	IP-SP	Min	3.00E+02	4.00E+02	6.00E+02	8.05E+02	9.00E+02	1.92E+03	2.00E+03	2.22E+03	2.49E+03	2.50E+03	2.60E+03	2.85E+03
		Max	4.98E+04	4.08E+02	6.15E+02	8.75E+02	9.09E+02	1.94E+07	2.05E+03	2.26E+03	2.49E+03	2.80E+03	2.90E+03	2.90E+03
		Mean	9.38E+03	4.01E+02	6.00E+02	8.42E+02	9.01E+02	1.33E+06	2.03E+03	2.24E+03	2.49E+03	2.53E+03	2.62E+03	2.88E+03
		STD	1.70E+04	1.97E+00	2.68E+00	4.54E+01	1.85E+00	4.04E+06	2.97E+01	3.71E+01	1.86E+02	1.46E+02	7.75E+01	1.85E+02
		ET	1.06E+00	9.51E-01	1.44E+00	1.25E+00	1.27E+00	1.07E+00	1.57E+00	1.70E+00	1.55E+00	1.45E+00	1.75E+00	2.00E+00
		NFEs	1.15E+05	1.15E+05	1.16E+05	1.15E+05	1.15E+05	1.15E+05	1.15E+05	1.15E+05	1.15E+05	1.15E+05	1.15E+05	1.15E+05

Table 9 continued from previous page

			F_1	F_2	F_3	F_4	F_5	F_6	F_7	F_8	F_9	F_{10}	F_{11}	F_{12}
GOBL-ABC	IP	Min	5.17E+03	4.05E+02	6.00E+02	8.21E+02	9.00E+02	7.82E+03	2.02E+03	2.23E+03	2.49E+03	2.50E+03	2.60E+03	2.86E+03
		Max	1.87E+04	4.07E+02	6.00E+02	8.39E+02	9.00E+02	4.88E+05	2.03E+03	2.24E+03	2.49E+03	2.51E+03	2.74E+03	2.90E+03
		Mean	1.16E+04	4.06E+02	6.00E+02	8.31E+02	9.00E+02	2.40E+05	2.03E+03	2.23E+03	2.49E+03	2.50E+03	2.61E+03	2.89E+03
		STD	1.20E+04	5.86E+00	6.25E-04	3.18E+01	1.28E-06	2.75E+05	2.81E+01	3.21E+01	1.89E+02	1.03E+02	2.95E+01	1.93E+02
		ET	8.16E-01	8.12E-01	9.87E-01	8.46E-01	8.76E-01	8.11E-01	1.04E+00	1.10E+00	1.02E+00	1.01E+00	1.16E+00	1.16E+00
		NFEs	1.00E+05	1.00E+05	1.00E+05	1.00E+05	1.00E+05	1.00E+05	1.00E+05	1.00E+05	1.00E+05	1.00E+05	1.00E+05	1.00E+05
	SP	Min	2.50E+03	4.05E+02	6.00E+02	8.17E+02	9.00E+02	6.04E+05	2.02E+03	2.23E+03	2.49E+03	2.50E+03	2.60E+03	2.90E+03
		Max	1.32E+04	4.06E+02	6.00E+02	8.45E+02	9.00E+02	7.24E+06	2.03E+03	2.25E+03	2.49E+03	2.50E+03	2.60E+03	2.90E+03
		Mean	7.20E+03	4.06E+02	6.00E+02	8.36E+02	9.00E+02	2.98E+06	2.03E+03	2.24E+03	2.49E+03	2.50E+03	2.60E+03	2.90E+03
		STD	7.57E+03	5.84E+00	1.87E-11	3.69E+01	2.15E-08	3.48E+06	2.86E+01	3.57E+01	1.86E+02	1.01E+02	2.11E-03	2.00E+02
		ET	8.75E-01	8.71E-01	1.07E+00	9.07E-01	9.51E-01	8.65E-01	1.12E+00	1.20E+00	1.10E+00	1.06E+00	1.30E+00	1.27E+00
		NFEs	1.16E+05	1.15E+05	1.15E+05	1.15E+05	1.16E+05	1.15E+05	1.15E+05	1.15E+05	1.15E+05	1.15E+05	1.16E+05	1.15E+05
	IP-SP	Min	2.49E+03	4.05E+02	6.00E+02	8.16E+02	9.00E+02	1.42E+06	2.02E+03	2.23E+03	2.49E+03	2.50E+03	2.60E+03	2.90E+03
		Max	1.58E+04	4.07E+02	6.00E+02	8.47E+02	9.00E+02	6.75E+06	2.03E+03	2.25E+03	2.49E+03	2.50E+03	2.60E+03	2.90E+03
		Mean	6.38E+03	4.06E+02	6.00E+02	8.37E+02	9.00E+02	2.82E+06	2.03E+03	2.24E+03	2.49E+03	2.50E+03	2.60E+03	2.90E+03
		STD	6.83E+03	5.89E+00	3.03E-11	3.79E+01	7.08E-09	3.08E+06	2.86E+01	3.64E+01	1.86E+02	1.01E+02	1.28E-07	2.00E+02
		ET	9.11E-01	9.17E-01	1.37E+00	1.05E+00	1.07E+00	9.37E-01	1.45E+00	1.55E+00	1.51E+00	1.37E+00	1.74E+00	1.86E+00
		NFEs	1.15E+05	1.15E+05	1.15E+05	1.15E+05	1.15E+05	1.15E+05	1.15E+05	1.15E+05	1.15E+05	1.15E+05	1.15E+05	1.15E+05

Table 9 continued from previous page

			F_1	F_2	F_3	F_4	F_5	F_6	F_7	F_8	F_9	F_{10}	F_{11}	F_{12}
QOBL-ABC	IP	Min	3.57E+03	4.05E+02	6.00E+02	8.20E+02	9.00E+02	2.30E+04	2.02E+03	2.22E+03	2.49E+03	2.50E+03	2.60E+03	2.87E+03
		Max	1.41E+04	4.06E+02	6.00E+02	8.40E+02	9.00E+02	4.91E+05	2.03E+03	2.23E+03	2.49E+03	2.51E+03	2.69E+03	2.90E+03
		Mean	8.19E+03	4.06E+02	6.00E+02	8.32E+02	9.00E+02	1.54E+05	2.03E+03	2.23E+03	2.49E+03	2.50E+03	2.61E+03	2.89E+03
		STD	8.46E+03	5.78E+00	1.04E-03	3.22E+01	3.06E-06	1.82E+05	2.78E+01	3.00E+01	1.89E+02	1.02E+02	2.80E+01	1.94E+02
		ET	6.26E-01	6.14E-01	7.72E-01	6.34E-01	6.94E-01	6.08E-01	7.82E-01	8.36E-01	7.61E-01	7.78E-01	8.67E-01	1.15E+00
		NFEs	1.00E+05	1.00E+05	1.00E+05	1.00E+05	1.00E+05	1.00E+05	1.00E+05	1.00E+05	1.00E+05	1.00E+05	1.00E+05	1.00E+05
	SP	Min	8.85E+02	4.05E+02	6.00E+02	8.26E+02	9.00E+02	8.74E+05	2.02E+03	2.23E+03	2.49E+03	2.50E+03	2.60E+03	2.90E+03
		Max	2.43E+03	4.07E+02	6.00E+02	8.50E+02	9.00E+02	6.17E+06	2.03E+03	2.24E+03	2.49E+03	2.63E+03	2.74E+03	2.90E+03
		Mean	1.48E+03	4.06E+02	6.00E+02	8.40E+02	9.00E+02	2.70E+06	2.03E+03	2.24E+03	2.49E+03	2.51E+03	2.60E+03	2.90E+03
		STD	1.23E+03	5.84E+00	1.32E-11	4.05E+01	1.92E-09	2.89E+06	2.83E+01	3.57E+01	1.86E+02	1.14E+02	2.48E+01	2.00E+02
		ET	8.64E-01	8.53E-01	1.01E+00	6.54E+01	8.72E-01	8.15E-01	1.02E+00	1.11E+00	1.02E+00	1.01E+00	1.19E+00	1.19E+00
		NFEs	1.15E+05	1.16E+05	1.15E+05	1.15E+05	1.15E+05	1.15E+05	1.15E+05	1.15E+05	1.15E+05	1.15E+05	1.15E+05	1.15E+05
	IP-SP	Min	9.03E+02	4.05E+02	6.00E+02	8.28E+02	9.00E+02	6.58E+05	2.02E+03	2.23E+03	2.49E+03	2.50E+03	2.60E+03	2.90E+03
		Max	1.94E+03	4.06E+02	6.00E+02	8.47E+02	9.00E+02	5.94E+06	2.03E+03	2.24E+03	2.49E+03	2.60E+03	2.75E+03	2.90E+03
		Mean	1.41E+03	4.06E+02	6.00E+02	8.41E+02	9.00E+02	2.38E+06	2.03E+03	2.24E+03	2.49E+03	2.51E+03	2.61E+03	2.90E+03
		STD	1.15E+03	5.63E+00	3.51E-11	4.08E+01	1.70E-09	2.62E+06	2.85E+01	3.53E+01	1.86E+02	1.07E+02	2.77E+01	2.00E+02
		ET	1.32E+00	1.33E+00	1.75E+00	1.45E+00	1.43E+00	1.32E+00	1.85E+00	1.98E+00	1.90E+00	1.74E+00	2.17E+00	2.25E+00
		NFEs	1.15E+05	1.15E+05	1.15E+05	1.15E+05	1.15E+05	1.15E+05	1.15E+05	1.15E+05	1.15E+05	1.15E+05	1.15E+05	1.15E+05

Table 9 continued from previous page

			F_1	F_2	F_3	F_4	F_5	F_6	F_7	F_8	F_9	F_{10}	F_{11}	F_{12}
QROBL-ABC	IP	Min	3.21E+03	4.05E+02	6.00E+02	8.22E+02	9.00E+02	1.45E+04	2.03E+03	2.23E+03	2.49E+03	2.50E+03	2.60E+03	2.86E+03
		Max	1.38E+04	4.06E+02	6.00E+02	8.40E+02	9.00E+02	5.25E+05	2.03E+03	2.24E+03	2.49E+03	2.51E+03	2.67E+03	2.90E+03
		Mean	7.97E+03	4.06E+02	6.00E+02	8.32E+02	9.00E+02	1.62E+05	2.03E+03	2.23E+03	2.49E+03	2.50E+03	2.60E+03	2.89E+03
		STD	7.99E+03	5.87E+00	6.48E-04	3.20E+01	1.21E-06	1.91E+05	2.80E+01	3.17E+01	1.88E+02	1.02E+02	1.32E+01	1.93E+02
		ET	1.33E+01	8.13E-01	1.01E+00	8.42E-01	8.82E-01	8.09E-01	1.05E+00	1.10E+00	1.01E+00	9.94E-01	1.17E+00	1.36E+00
		NFEs	1.00E+05	1.00E+05	1.00E+05	1.00E+05	1.00E+05	1.00E+05	1.00E+05	1.00E+05	1.00E+05	1.00E+05	1.00E+05	1.00E+05
	SP	Min	3.58E+02	4.05E+02	6.00E+02	8.20E+02	9.00E+02	1.85E+05	2.02E+03	2.23E+03	2.49E+03	2.50E+03	2.60E+03	2.90E+03
		Max	6.37E+02	4.06E+02	6.00E+02	8.40E+02	9.00E+02	8.10E+06	2.03E+03	2.24E+03	2.49E+03	2.50E+03	2.60E+03	2.90E+03
		Mean	4.56E+02	4.05E+02	6.00E+02	8.27E+02	9.00E+02	2.92E+06	2.03E+03	2.24E+03	2.49E+03	2.50E+03	2.60E+03	2.90E+03
		STD	1.67E+02	5.44E+00	4.90E-12	2.75E+01	3.24E-10	3.34E+06	2.78E+01	3.54E+01	1.86E+02	1.00E+02	2.92E-12	2.00E+02
		ET	1.08E+00	1.07E+00	1.29E+00	1.13E+00	1.16E+00	1.06E+00	1.33E+00	1.41E+00	1.31E+00	1.27E+00	1.51E+00	1.48E+00
		NFEs	1.15E+05	1.15E+05	1.15E+05	1.15E+05	1.15E+05	1.15E+05	1.15E+05	1.15E+05	1.16E+05	1.15E+05	1.15E+05	1.16E+05
	IP-SP	Min	3.43E+02	4.05E+02	6.00E+02	8.17E+02	9.00E+02	1.62E+05	2.02E+03	2.23E+03	2.49E+03	2.50E+03	2.60E+03	2.90E+03
		Max	5.45E+02	4.06E+02	6.00E+02	8.30E+02	9.00E+02	6.86E+06	2.03E+03	2.25E+03	2.49E+03	2.50E+03	2.60E+03	2.90E+03
		Mean	4.35E+02	4.05E+02	6.00E+02	8.25E+02	9.00E+02	1.93E+06	2.03E+03	2.24E+03	2.49E+03	2.50E+03	2.60E+03	2.90E+03
		STD	1.42E+02	5.47E+00	6.34E-12	2.50E+01	3.63E-10	2.32E+06	2.71E+01	3.65E+01	1.86E+02	1.00E+02	2.36E-11	2.00E+02
		ET	1.31E+00	1.30E+00	1.79E+00	1.44E+00	1.47E+00	1.36E+00	1.86E+00	1.98E+00	1.92E+00	1.77E+00	2.15E+00	2.24E+00
		NFEs	1.15E+05	1.15E+05	1.15E+05	1.15E+05	1.15E+05	1.15E+05	1.15E+05	1.15E+05	1.15E+05	1.15E+05	1.15E+05	1.15E+05

Table 10: Performance metrics for ABC variants across 12 functions ($D = 20$)

			F_1	F_2	F_3	F_4	F_5	F_6	F_7	F_8	F_9	F_{10}	F_{11}	F_{12}
ABC		Min	1.27E+04	4.05E+02	6.00E+02	8.24E+02	9.00E+02	5.34E+06	2.03E+03	2.23E+03	2.49E+03	2.50E+03	2.60E+03	2.90E+03
		Max	5.61E+04	4.07E+02	6.00E+02	8.54E+02	9.00E+02	3.28E+07	2.05E+03	2.26E+03	2.54E+03	2.74E+03	2.75E+03	2.90E+03
		Mean	3.15E+04	4.06E+02	6.00E+02	8.43E+02	9.00E+02	1.53E+07	2.04E+03	2.24E+03	2.50E+03	2.56E+03	2.62E+03	2.90E+03
		STD	1.08E+05	1.79E+01	1.37E-01	1.24E+02	9.10E+00	3.58E+07	1.37E+02	1.00E+02	1.88E+02	3.33E+03	3.00E+02	2.00E+02
		ET	1.87E+00	1.94E+00	2.72E+00	1.54E+00	9.36E-01	8.32E-01	1.26E+00	1.36E+00	1.34E+00	1.18E+00	1.52E+00	2.94E+00
		NFEs	1.00E+05	1.00E+05	1.00E+05	1.00E+05	1.00E+05	1.00E+05	1.00E+05	1.00E+05	1.00E+05	1.00E+05	1.00E+05	1.00E+05
BOBL-ABC	IP	Min	3.59E+04	4.17E+02	6.00E+02	9.06E+02	9.03E+02	1.09E+07	2.10E+03	2.26E+03	2.48E+03	2.62E+03	2.90E+03	2.90E+03
		Max	1.26E+05	4.19E+02	6.00E+02	9.42E+02	9.26E+02	6.19E+07	2.16E+03	2.32E+03	2.50E+03	6.92E+03	2.90E+03	2.90E+03
		Mean	8.25E+04	4.18E+02	6.00E+02	9.21E+02	9.08E+02	2.88E+07	2.13E+03	2.29E+03	2.49E+03	5.01E+03	2.90E+03	2.90E+03
		STD	8.49E+04	1.80E+01	1.19E-01	1.22E+02	9.34E+00	3.14E+07	1.29E+02	8.67E+01	1.90E+02	3.09E+03	3.00E+02	2.00E+02
		ET	8.01E-01	8.18E-01	1.20E+00	9.10E-01	9.44E-01	8.35E-01	1.30E+00	1.35E+00	1.31E+00	1.17E+00	1.52E+00	1.59E+00
		NFEs	1.00E+05	1.00E+05	1.00E+05	1.00E+05	1.00E+05	1.00E+05	1.00E+05	1.00E+05	1.00E+05	1.00E+05	1.00E+05	1.00E+05
	SP	Min	9.75E+04	4.17E+02	6.00E+02	9.09E+02	9.00E+02	2.36E+07	2.09E+03	2.27E+03	2.47E+03	5.70E+03	2.90E+03	2.90E+03
		Max	3.65E+05	4.19E+02	6.00E+02	9.42E+02	9.05E+02	8.31E+07	2.15E+03	2.40E+03	2.49E+03	7.10E+03	2.90E+03	2.90E+03
		Mean	2.13E+05	4.18E+02	6.00E+02	9.28E+02	9.01E+02	5.33E+07	2.13E+03	2.32E+03	2.47E+03	6.60E+03	2.90E+03	2.90E+03
		STD	2.25E+05	1.76E+01	8.85E-03	1.29E+02	1.20E+00	5.55E+07	1.26E+02	1.22E+02	1.73E+02	4.22E+03	3.00E+02	2.00E+02
		ET	8.72E-01	8.93E-01	1.31E+00	1.00E+00	1.12E+00	1.12E+00	1.72E+00	1.86E+00	1.80E+00	1.61E+00	2.11E+00	2.24E+00
		NFEs	1.15E+05	1.15E+05	1.15E+05	1.15E+05	1.15E+05	1.15E+05	1.15E+05	1.15E+05	1.15E+05	1.15E+05	1.15E+05	1.15E+05
	IP-SP	Min	7.68E+04	4.17E+02	6.00E+02	9.01E+02	9.00E+02	2.00E+07	2.08E+03	2.29E+03	2.47E+03	2.61E+03	2.90E+03	2.90E+03
		Max	2.87E+05	4.19E+02	6.00E+02	9.43E+02	9.03E+02	1.02E+08	2.16E+03	2.36E+03	2.50E+03	7.01E+03	2.91E+03	2.90E+03
		Mean	1.52E+05	4.18E+02	6.00E+02	9.30E+02	9.01E+02	5.76E+07	2.12E+03	2.32E+03	2.47E+03	6.34E+03	2.90E+03	2.90E+03
		STD	1.59E+05	1.76E+01	7.37E-03	1.30E+02	8.73E-01	6.10E+07	1.25E+02	1.22E+02	1.75E+02	4.05E+03	3.01E+02	2.00E+02
		ET	9.74E-01	9.97E-01	1.45E+00	1.11E+00	1.15E+00	1.03E+00	1.54E+00	1.66E+00	1.67E+00	1.50E+00	2.06E+00	2.16E+00
		FES	1.00E+05	1.00E+05	1.00E+05	1.00E+05	1.00E+05	1.00E+05	1.00E+05	1.00E+05	1.00E+05	1.00E+05	1.00E+05	1.00E+05

Table 10 continued from previous page

			F_1	F_2	F_3	F_4	F_5	F_6	F_7	F_8	F_9	F_{10}	F_{11}	F_{12}
COOBL-ABC	IP	Min	5.96E+04	4.17E+02	6.00E+02	9.08E+02	9.02E+02	1.49E+07	2.09E+03	2.26E+03	2.48E+03	2.59E+03	2.90E+03	2.90E+03
		Max	2.00E+05	4.19E+02	6.00E+02	9.38E+02	9.22E+02	7.37E+07	2.16E+03	2.34E+03	2.51E+03	7.10E+03	2.90E+03	2.90E+03
		Mean	1.09E+05	4.18E+02	6.00E+02	9.25E+02	9.07E+02	3.96E+07	2.13E+03	2.29E+03	2.49E+03	5.63E+03	2.90E+03	2.90E+03
		STD	1.12E+05	1.79E+01	1.21E-01	1.26E+02	9.25E+00	4.23E+07	1.33E+02	9.35E+01	1.91E+02	3.64E+03	3.00E+02	2.00E+02
		ET	6.42E+00	7.01E-01	9.84E-01	7.50E-01	8.01E-01	6.93E-01	1.06E+00	1.15E+00	1.10E+00	1.03E+00	1.27E+00	1.34E+00
		NFEs	1.00E+05	1.00E+05	1.00E+05	1.00E+05	1.00E+05	1.00E+05	1.00E+05	1.00E+05	1.00E+05	1.00E+05	1.00E+05	1.00E+05
	SP	Min	5.16E+04	4.10E+02	6.00E+02	8.31E+02	9.02E+02	2.03E+03	2.02E+03	2.23E+03	2.47E+03	2.50E+03	2.90E+03	2.90E+03
		Max	2.57E+05	4.80E+02	7.17E+02	1.01E+03	7.27E+03	7.08E+08	2.38E+03	2.64E+03	2.70E+03	7.56E+03	3.00E+03	2.90E+03
		Mean	1.18E+05	4.17E+02	6.12E+02	9.48E+02	1.84E+03	3.50E+07	2.16E+03	2.35E+03	2.48E+03	5.50E+03	2.93E+03	2.90E+03
		STD	1.27E+05	2.32E+01	3.33E+01	1.58E+02	1.57E+03	1.34E+08	1.97E+02	1.88E+02	1.87E+02	3.70E+03	3.30E+02	2.00E+02
		ET	8.38E-01	7.72E-01	1.12E+00	9.08E-01	9.50E-01	8.76E-01	1.28E+00	1.40E+00	1.32E+00	1.18E+00	1.42E+00	1.62E+00
		NFEs	1.15E+05	1.15E+05	1.15E+05	1.15E+05	1.15E+05	1.15E+05	1.15E+05	1.15E+05	1.15E+05	1.15E+05	1.15E+05	1.15E+05
	IP-SP	Min	4.72E+04	4.17E+02	6.00E+02	9.19E+02	9.00E+02	1.96E+07	2.09E+03	2.27E+03	2.47E+03	2.50E+03	2.90E+03	2.90E+03
		Max	2.64E+05	4.19E+02	6.00E+02	9.42E+02	9.04E+02	1.16E+08	2.16E+03	2.37E+03	2.48E+03	7.18E+03	2.94E+03	2.90E+03
		Mean	1.38E+05	4.18E+02	6.00E+02	9.32E+02	9.01E+02	5.95E+07	2.12E+03	2.32E+03	2.47E+03	5.56E+03	2.90E+03	2.90E+03
		STD	1.45E+05	1.80E+01	8.13E-03	1.32E+02	1.15E+00	6.36E+07	1.21E+02	1.20E+02	1.72E+02	3.68E+03	3.02E+02	2.00E+02
		ET	6.24E-01	6.40E-01	9.22E-01	7.46E-01	7.22E-01	6.93E-01	1.02E+00	1.07E+00	1.02E+00	9.43E-01	1.17E+00	1.26E+00
		NFEs	1.00E+05	1.00E+05	1.00E+05	1.00E+05	1.00E+05	1.00E+05	1.00E+05	1.00E+05	1.00E+05	1.00E+05	1.00E+05	1.00E+05

Table 10 continued from previous page

			F_1	F_2	F_3	F_4	F_5	F_6	F_7	F_8	F_9	F_{10}	F_{11}	F_{12}
GOBL-ABC	IP	Min	5.25E+04	4.17E+02	6.00E+02	9.08E+02	9.01E+02	6.12E+06	2.10E+03	2.25E+03	2.47E+03	2.62E+03	2.90E+03	2.90E+03
		Max	1.46E+05	4.19E+02	6.00E+02	9.38E+02	9.43E+02	5.52E+07	2.16E+03	2.31E+03	2.50E+03	6.97E+03	2.90E+03	2.90E+03
		Mean	8.58E+04	4.18E+02	6.00E+02	9.24E+02	9.08E+02	2.66E+07	2.13E+03	2.28E+03	2.49E+03	4.84E+03	2.90E+03	2.90E+03
		STD	8.86E+04	1.79E+01	1.28E-01	1.24E+02	1.12E+01	2.92E+07	1.36E+02	8.59E+01	1.87E+02	3.00E+03	3.00E+02	2.00E+02
		ET	8.22E-01	8.38E-01	1.22E+00	9.32E-01	9.53E-01	8.51E-01	1.28E+00	1.38E+00	1.33E+00	1.19E+00	1.55E+00	1.61E+00
		NFEs	1.00E+05	1.00E+05	1.00E+05	1.00E+05	1.00E+05	1.00E+05	1.00E+05	1.00E+05	1.00E+05	1.00E+05	1.00E+05	1.00E+05
	SP	Min	6.84E+04	4.17E+02	6.00E+02	9.09E+02	9.00E+02	2.14E+07	2.09E+03	2.27E+03	2.47E+03	2.50E+03	2.88E+03	2.90E+03
		Max	2.84E+05	4.19E+02	6.00E+02	9.40E+02	9.04E+02	1.16E+08	2.16E+03	2.40E+03	2.49E+03	7.23E+03	3.00E+03	2.90E+03
		Mean	1.60E+05	4.18E+02	6.00E+02	9.27E+02	9.01E+02	5.74E+07	2.13E+03	2.31E+03	2.47E+03	6.69E+03	2.91E+03	2.90E+03
		STD	1.68E+05	1.78E+01	8.26E-03	1.27E+02	9.09E-01	6.26E+07	1.27E+02	1.16E+02	1.73E+02	4.36E+03	3.07E+02	2.00E+02
		ET	9.02E-01	9.01E-01	1.32E+00	1.00E+00	1.03E+00	9.14E-01	1.40E+00	1.56E+00	1.47E+00	1.32E+00	1.71E+00	1.81E+00
		NFEs	1.15E+05	1.15E+05	1.15E+05	1.15E+05	1.15E+05	1.15E+05	1.15E+05	1.15E+05	1.15E+05	1.15E+05	1.15E+05	1.15E+05
	IP-SP	Min	6.37E+04	4.17E+02	6.00E+02	9.31E+02	9.01E+02	1.07E+08	2.11E+03	2.29E+03	2.48E+03	4.75E+03	2.90E+03	2.90E+03
		Max	2.15E+06	4.19E+02	6.00E+02	9.74E+02	9.32E+02	7.78E+08	2.26E+03	2.76E+03	2.70E+03	7.70E+03	2.90E+03	2.90E+03
		Mean	3.84E+05	4.18E+02	6.00E+02	9.57E+02	9.08E+02	3.84E+08	2.21E+03	2.46E+03	2.56E+03	7.21E+03	2.90E+03	2.90E+03
		STD	5.60E+05	1.78E+01	2.31E-01	1.57E+02	1.15E+01	4.13E+08	2.14E+02	2.80E+02	2.70E+02	4.84E+03	3.00E+02	2.00E+02
		ET	8.05E-01	8.25E-01	1.20E+00	9.32E-01	9.29E-01	8.34E-01	1.26E+00	1.35E+00	1.31E+00	1.18E+00	1.53E+00	1.60E+00
		NFEs	1.00E+05	1.00E+05	1.00E+05	1.00E+05	1.00E+05	1.00E+05	1.00E+05	1.00E+05	1.00E+05	1.00E+05	1.00E+05	1.00E+05

Table 10 continued from previous page

			F_1	F_2	F_3	F_4	F_5	F_6	F_7	F_8	F_9	F_{10}	F_{11}	F_{12}
QOBL-ABC	IP	Min	3.21E+04	4.17E+02	6.00E+02	9.11E+02	9.01E+02	5.34E+06	2.08E+03	2.26E+03	2.47E+03	2.54E+03	2.90E+03	2.90E+03
		Max	9.17E+04	4.19E+02	6.00E+02	9.37E+02	9.26E+02	3.55E+07	2.16E+03	2.32E+03	2.49E+03	6.67E+03	2.91E+03	2.90E+03
		Mean	6.24E+04	4.18E+02	6.00E+02	9.23E+02	9.07E+02	1.64E+07	2.12E+03	2.28E+03	2.48E+03	3.57E+03	2.90E+03	2.90E+03
		STD	6.45E+04	1.79E+01	1.34E-01	1.24E+02	8.71E+00	1.78E+07	1.24E+02	8.20E+01	1.79E+02	1.76E+03	3.01E+02	2.00E+02
		ET	6.85E-01	7.49E-01	1.46E+01	7.28E-01	7.16E-01	6.47E-01	9.80E-01	1.10E+00	1.02E+00	9.22E-01	1.18E+00	1.28E+00
		NFEs	1.00E+05	1.00E+05	1.00E+05	1.00E+05	1.00E+05	1.00E+05	1.00E+05	1.00E+05	1.00E+05	1.00E+05	1.00E+05	1.00E+05
	SP	Min	4.21E+04	4.17E+02	6.00E+02	9.12E+02	9.00E+02	1.57E+07	2.09E+03	2.27E+03	2.47E+03	6.38E+03	2.90E+03	2.90E+03
		Max	1.79E+05	4.19E+02	6.00E+02	9.47E+02	9.09E+02	1.17E+08	2.17E+03	2.37E+03	2.48E+03	7.29E+03	2.90E+03	2.90E+03
		Mean	8.92E+04	4.18E+02	6.00E+02	9.35E+02	9.01E+02	5.63E+07	2.13E+03	2.32E+03	2.47E+03	7.01E+03	2.90E+03	2.90E+03
		STD	9.51E+04	1.78E+01	7.68E-03	1.36E+02	2.25E+00	6.20E+07	1.34E+02	1.25E+02	1.72E+02	4.61E+03	3.00E+02	2.00E+02
		ET	1.07E+00	1.27E+01	1.32E+00	1.08E+00	1.11E+00	1.04E+00	1.43E+00	1.53E+00	1.47E+00	3.50E+01	1.56E+00	1.66E+00
		NFEs	1.07E+00	1.27E+01	1.32E+00	1.08E+00	1.11E+00	1.04E+00	1.43E+00	1.53E+00	1.47E+00	3.50E+01	1.56E+00	1.66E+00
	IP-SP	Min	3.91E+04	4.17E+02	6.00E+02	9.12E+02	9.00E+02	6.22E+06	2.08E+03	2.26E+03	2.47E+03	2.50E+03	2.90E+03	2.90E+03
		Max	1.32E+05	4.19E+02	6.00E+02	9.45E+02	9.05E+02	5.96E+07	2.16E+03	2.35E+03	2.48E+03	7.19E+03	2.92E+03	2.90E+03
		Mean	7.89E+04	4.18E+02	6.00E+02	9.34E+02	9.01E+02	2.69E+07	2.12E+03	2.30E+03	2.47E+03	6.33E+03	2.90E+03	2.90E+03
		STD	8.24E+04	1.78E+01	8.14E-03	1.34E+02	1.72E+00	2.94E+07	1.20E+02	1.06E+02	1.69E+02	4.09E+03	3.01E+02	2.00E+02
		ET	6.38E-01	6.51E-01	9.42E-01	7.24E-01	7.38E-01	6.92E-01	1.03E+00	1.12E+00	1.06E+00	9.60E-01	1.22E+00	1.28E+00
		NFEs	1.00E+05	1.00E+05	1.00E+05	1.00E+05	1.00E+05	1.00E+05	1.00E+05	1.00E+05	1.00E+05	1.00E+05	1.00E+05	1.00E+05

Table 10 continued from previous page

			F_1	F_2	F_3	F_4	F_5	F_6	F_7	F_8	F_9	F_{10}	F_{11}	F_{12}
QROBL-ABC	IP	Min	2.58E+04	4.17E+02	6.00E+02	8.98E+02	9.01E+02	7.27E+06	2.11E+03	2.27E+03	2.47E+03	2.51E+03	2.90E+03	2.90E+03
		Max	1.12E+05	4.19E+02	6.00E+02	9.34E+02	9.25E+02	3.49E+07	2.14E+03	2.32E+03	2.49E+03	6.75E+03	2.91E+03	2.90E+03
		Mean	6.56E+04	4.18E+02	6.00E+02	9.20E+02	9.07E+02	1.66E+07	2.13E+03	2.29E+03	2.48E+03	3.23E+03	2.90E+03	2.90E+03
		STD	6.79E+04	1.79E+01	1.13E-01	1.20E+02	9.43E+00	1.79E+07	1.27E+02	8.71E+01	1.77E+02	1.48E+03	3.01E+02	2.00E+02
		ET	8.28E-01	8.50E-01	1.22E+00	9.38E-01	9.62E-01	8.65E-01	1.29E+00	1.39E+00	1.34E+00	1.23E+00	1.55E+00	1.63E+00
		FES	1.00E+05	1.00E+05	1.00E+05	1.00E+05	1.00E+05	1.00E+05	1.00E+05	1.00E+05	1.00E+05	1.00E+05	1.00E+05	1.00E+05
	SP	Min	1.28E+04	4.17E+02	6.00E+02	9.09E+02	9.00E+02	4.47E+06	2.09E+03	2.26E+03	2.47E+03	2.50E+03	2.90E+03	2.90E+03
		Max	2.77E+04	4.76E+02	6.00E+02	9.36E+02	9.04E+02	8.19E+07	2.17E+03	2.36E+03	2.47E+03	6.53E+03	3.00E+03	2.90E+03
		Mean	2.19E+04	4.20E+02	6.00E+02	9.26E+02	9.01E+02	3.71E+07	2.12E+03	2.31E+03	2.47E+03	3.05E+03	2.91E+03	2.90E+03
		STD	2.18E+04	2.22E+01	5.38E-03	1.26E+02	1.41E+00	4.12E+07	1.17E+02	1.12E+02	1.66E+02	1.46E+03	3.11E+02	2.00E+02
		ET	1.29E+00	1.28E+00	1.71E+00	1.39E+00	1.42E+00	1.32E+00	1.80E+00	1.94E+00	1.86E+00	1.68E+00	2.12E+00	2.19E+00
		NFEs	1.15E+05	1.15E+05	1.15E+05	1.15E+05	1.15E+05	1.15E+05	1.15E+05	1.15E+05	1.15E+05	1.15E+05	1.15E+05	1.15E+05
	IP-SP	Min	1.13E+04	4.17E+02	6.00E+02	9.08E+02	9.00E+02	3.26E+06	2.07E+03	2.27E+03	2.47E+03	2.50E+03	2.90E+03	2.90E+03
		Max	2.97E+04	4.49E+02	6.00E+02	9.44E+02	9.02E+02	3.42E+07	2.15E+03	2.34E+03	2.47E+03	6.32E+03	3.00E+03	2.90E+03
		Mean	2.08E+04	4.19E+02	6.00E+02	9.31E+02	9.01E+02	1.84E+07	2.11E+03	2.30E+03	2.47E+03	2.65E+03	2.91E+03	2.90E+03
		STD	2.09E+04	1.95E+01	5.38E-03	1.31E+02	1.06E+00	1.99E+07	1.09E+02	1.01E+02	1.66E+02	7.28E+02	3.14E+02	2.00E+02
		ET	8.07E-01	8.24E-01	1.20E+00	9.15E-01	9.38E-01	8.39E-01	1.27E+00	1.37E+00	1.32E+00	1.19E+00	1.53E+00	1.61E+00
		NFEs	1.00E+05	1.00E+05	1.00E+05	1.00E+05	1.00E+05	1.00E+05	1.00E+05	1.00E+05	1.00E+05	1.00E+05	1.00E+05	1.00E+05

Table 11: Friedman Mean Rank Results for DE variants

Algorithm	Diemension	Friedman Mean Rank	Rank	Diemension	Friedman Mean Rank	Rank
DE_BOBL_IP	$D = 10$	17.625	6	$D = 20$	32.167	12
DE_BOBL_SP		21.292	10		26.875	3
DE_BOBL_IP_SP		21.542	11		30.625	7
DE_QOBL_IP		17.917	7		30.167	6
DE_QOBL_SP		17.458	5		28.25	4
DE_QOBL_IP_SP		15.167	3		31.125	9
DE_GOBL_IP		18.458	8		31.792	11
DE_GOBL_SP		17.708	6		32.458	13
DE_GOBL_IP_SP		17.208	4		31.708	10
DE_COOBL_IP		23.375	12		29.083	5
DE_COOBL_SP		36.917	14		38.417	14
DE_COOBL_IP_SP		43.250	15		46.833	15
DE_QROBL_IP		18.208	9		31.083	8
DE_QROBL_SP		12.958	2		**24**	**1**
DE_QROBL_IP_SP		**11.708**	**1**		26.292	2

Table 12: Friedman Mean Rank Results for GA variants

Algorithm	Diemension	Friedman Mean Rank	Rank	Diemension	Friedman Mean Rank	Rank
GA_BOBL_IP	$D = 10$	48.833	10	$D = 20$	43.25	9
GA_BOBL_SP		47.250	8		42.25	7
GA_BOBL_IP_SP		49.583	11		44.417	10
GA_QOBL_IP		46.917	5		39.917	4
GA_QOBL_SP		**43.583**	**1**		39.167	3
GA_QOBL_IP_SP		46.333	2		42.667	8
GA_GOBL_IP		53.333	12		46.25	12
GA_GOBL_SP		44.250	3		39.917	4
GA_GOBL_IP_SP		50.750	12		42	6
GA_COOBL_IP		57.417	14		45.833	11
GA_COOBL_SP		52.500	13		38.75	2
GA_COOBL_IP_SP		60.167	15		46.417	13
GA_QROBL_IP		47.083	7		40.083	5
GA_QROBL_SP		47.583	9		38.75	2
GA_QROBL_IP_SP		46.583	4		**37.333**	**1**

Table 13: Friedman Mean Rank Results for PSO variants

Algorithm	Diemension	Friedman Mean Rank	Rank	Diemension	Friedman Mean Rank	Rank
PSO_BOBL_IP	$D = 10$	41.625	9	$D = 20$	41.167	8
PSO_BOBL_SP		38.125	5		40.958	7
PSO_BOBL_IP_SP		36.333	4		36.792	2
PSO_QOBL_IP		38.417	6		42.833	12
PSO_QOBL_SP		39.208	7		43.5	13
PSO_QOBL_IP_SP		42.833	11		41.75	10
PSO_GOBL_IP		**33.333**	**1**		39.125	4
PSO_GOBL_SP		33.917	2		41.208	9
PSO_GOBL_IP_SP		35	3		**35.333**	**1**
PSO_COOBL_IP		42.333	10		41.833	11
PSO_COOBL_SP		53.583	14		51	14
PSO_COOBL_IP_SP		54.625	15		51.667	15
PSO_QROBL_IP		40.5	8		39.583	5

Algorithm	Diemension	Friedman Mean Rank	Rank	Diemension	Friedman Mean Rank	Rank
PSO_QROBL_SP		43.625	112		38.75	3
PSO_QROBL_IP_SP		44.25	13		40.625	6

Table 14: Friedman Mean Rank Results for HS variants

Algorithm	Diemension	Friedman Mean Rank	Rank	Diemension	Friedman Mean Rank	Rank
HS_BOBL_IP	$D = 10$	37.083	8	$D = 20$	34.5	11
HS_BOBL_SP		35.083	4		33.333	10
HS_BOBL_IP_SP		34.833	2		32.833	8
HS_QOBL_IP		**33.083**	**1**		31.5	3
HS_QOBL_SP		37.167	9		34.667	12
HS_QOBL_IP_SP		41	14		32.833	8
HS_GOBL_IP		35.667	5		32.667	7
HS_GOBL_SP		37.5	10		34.5	11
HS_GOBL_IP_SP		34.167	3		32.083	5
HS_COOBL_IP		42.333	15		34.917	13
HS_COOBL_SP		36	7		**28.833**	**1**
HS_COOBL_IP_SP		39.583	12		29.167	2
HS_QROBL_IP		40.167	13		32.333	6
HS_QROBL_SP		37.083	8		31.667	4
HS_QROBL_IP_SP		37.583	11		33	9

Table 15: Friedman Mean Rank Results for ABC variants

Algorithm	Diemension	Friedman Mean Rank	Rank	Diemension	Friedman Mean Rank	Rank
ABC_BOBL_IP	$D = 10$	42.833	10	$D = 20$	44.583	11
ABC_BOBL_SP		41.083	3		41	6
ABC_BOBL_IP_SP		41.167	4		41.083	7
ABC_QOBL_IP		41.417	6		37.75	4
ABC_QOBL_SP		41.417	6		41.583	9
ABC_QOBL_IP_SP		41.333	5		39.583	5
ABC_GOBL_IP		41.833	7		43.667	10
ABC_GOBL_SP		42	8		41.417	8
ABC_GOBL_IP_SP		42.083	9		41.083	7
ABC_COOBL_IP		43.917	12		46.417	12
ABC_COOBL_SP		43.583	11		53.5	13
ABC_COOBL_IP_SP		47.583	13		56.667	14
ABC_QROBL_IP		40.583	2		37.083	3
ABC_QROBL_SP		**35.083**	**1**		36.917	2
ABC_QROBL_IP_SP		**35.083**	**1**		**34.833**	**1**

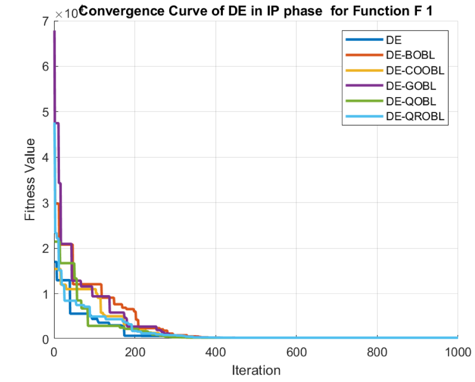

(a) F_1

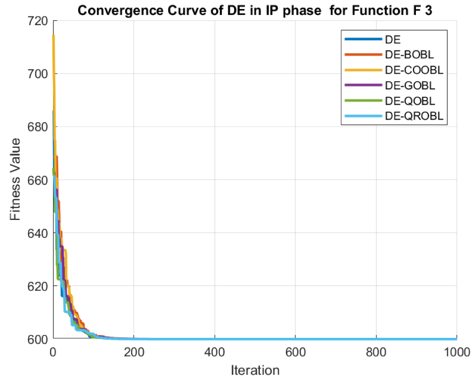

(b) F_3

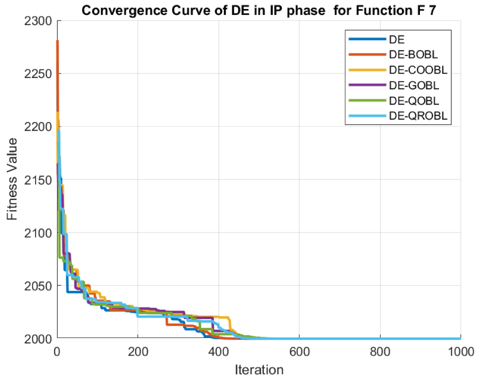

(c) F_7

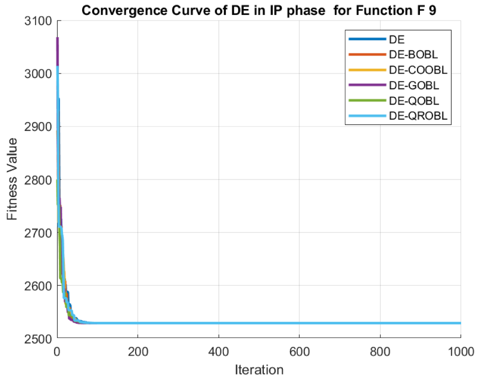

(d) F_9

Fig. 1: Convergence curves of DE and its variant during IP phase ($D = 10$).

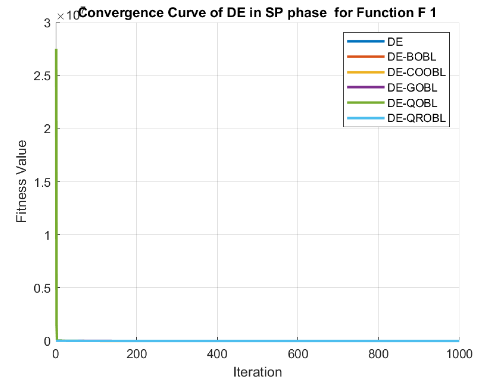

(a) F_1

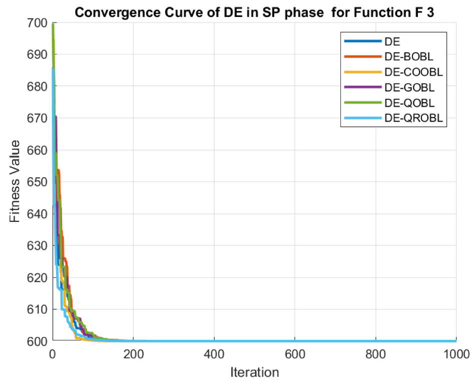

(b) F_3

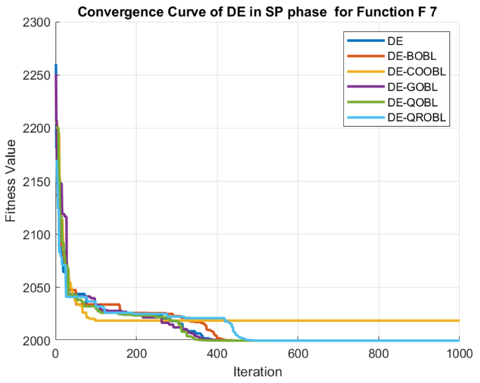

(c) F_7

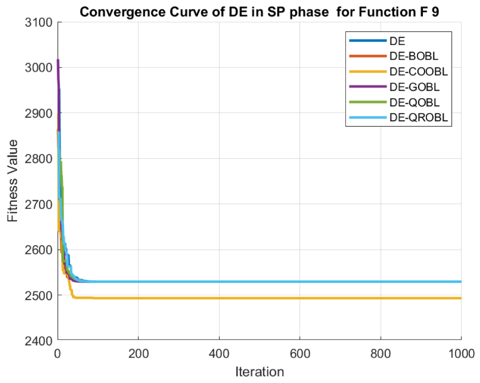

(d) F_9

Fig. 2: Convergence curves of DE and its variant during SP phase ($D = 10$).

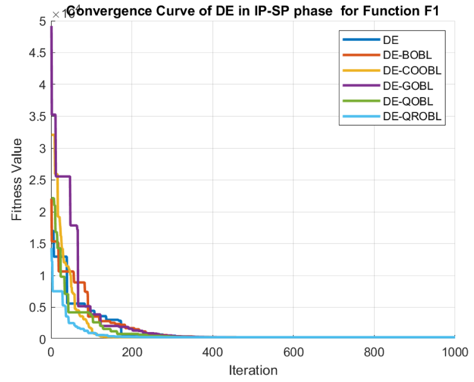

(a) F_1

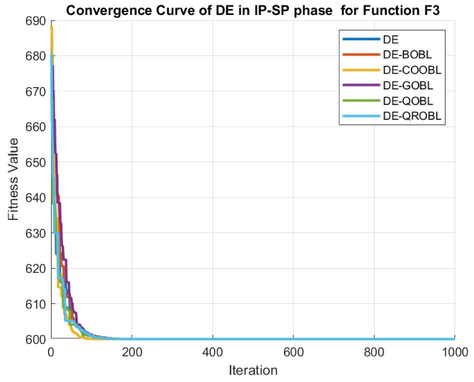

(b) F_3

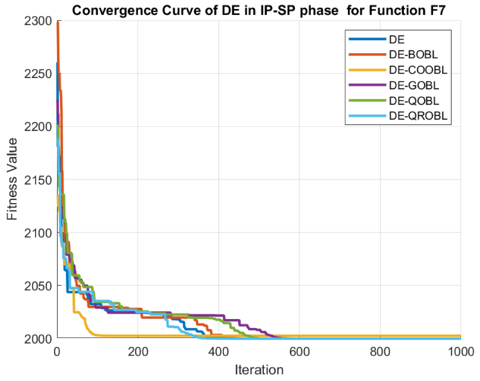

(c) F_7

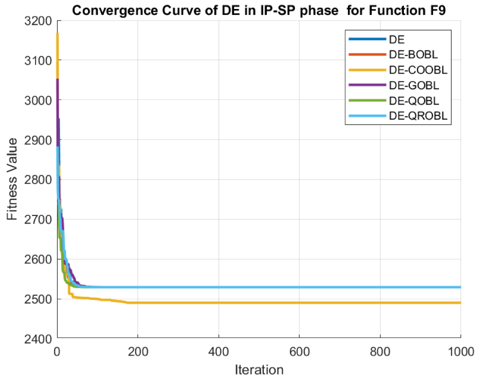

(d) F_9

Fig. 3: Convergence curves of DE and its variant during IP-SP phase ($D = 10$).

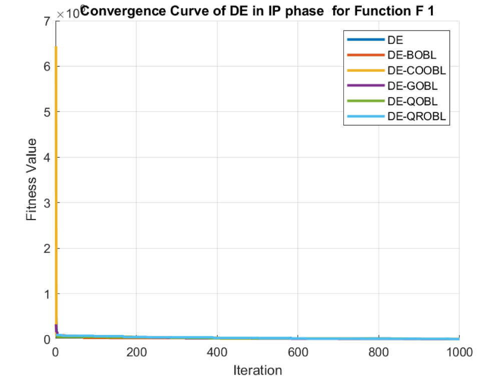

(a) F_1

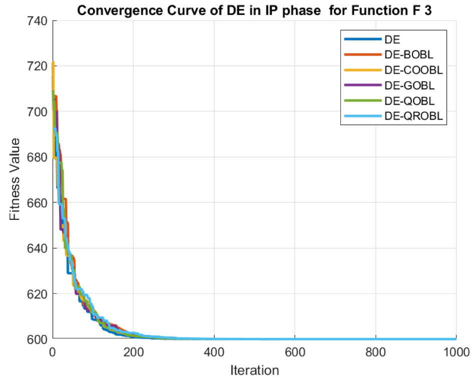

(b) F_3

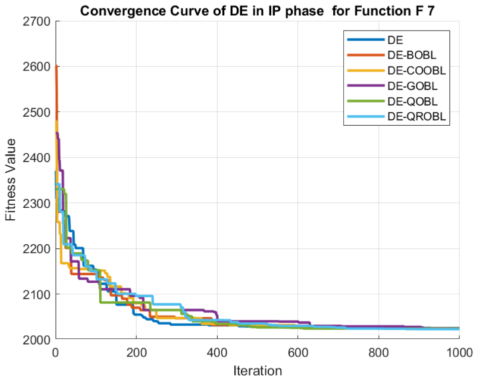

(c) F_7

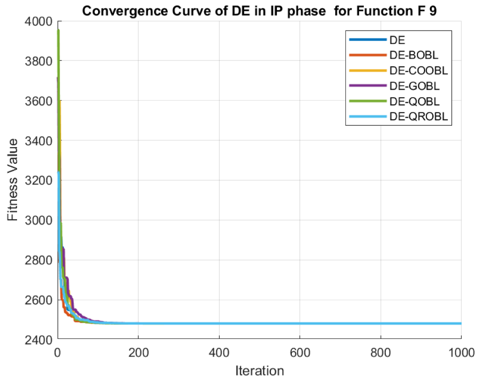

(d) F_9

Fig. 4: Convergence curves of DE and its variant during IP phase ($D = 20$).

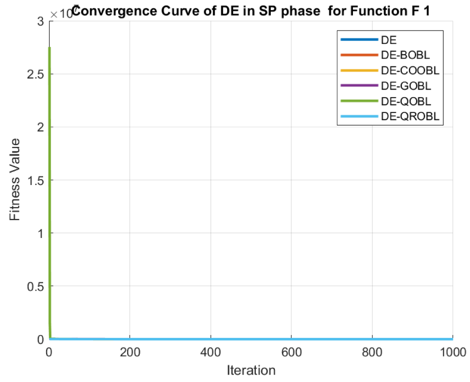

(a) F_1

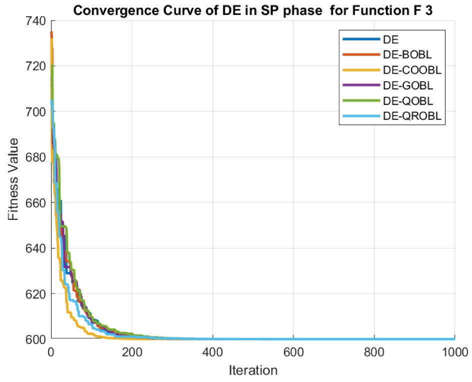

(b) F_3

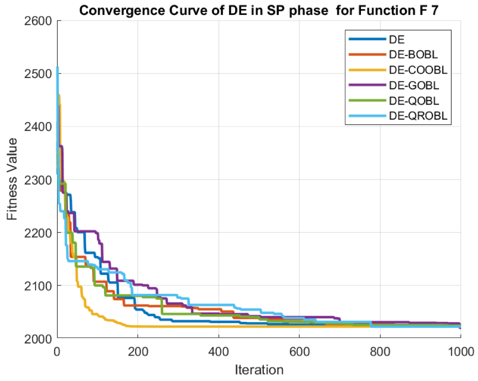

(c) F_7

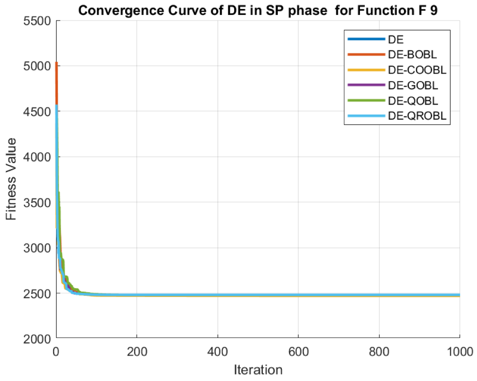

(d) F_9

Fig. 5: Convergence curves of DE and its variant during SP phase ($D = 20$).

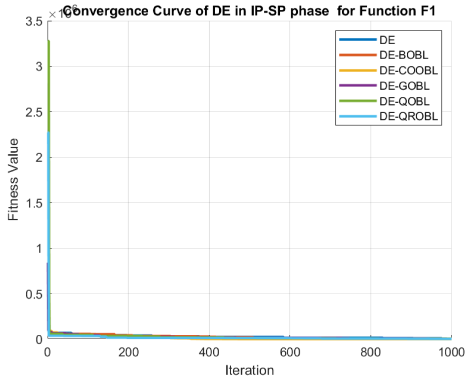

(a) F_1

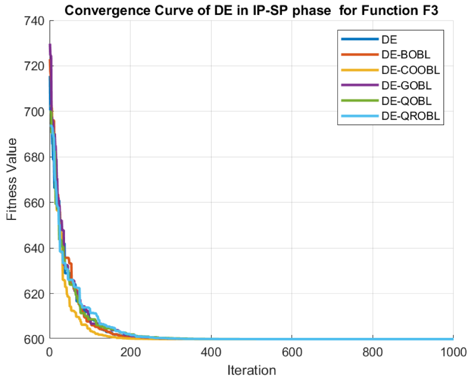

(b) F_3

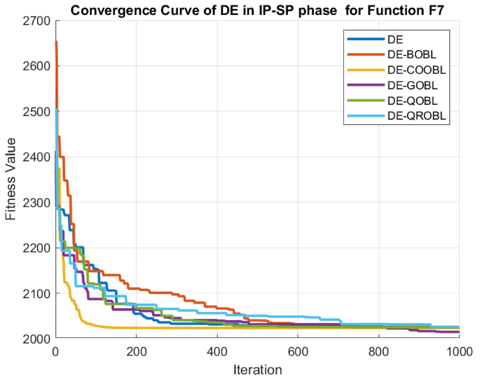

(c) F_7

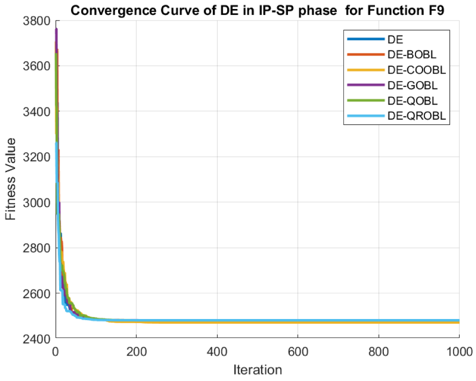

(d) F_9

Fig. 6: Convergence curves of DE and its variant during IP-SP phase ($D = 20$).

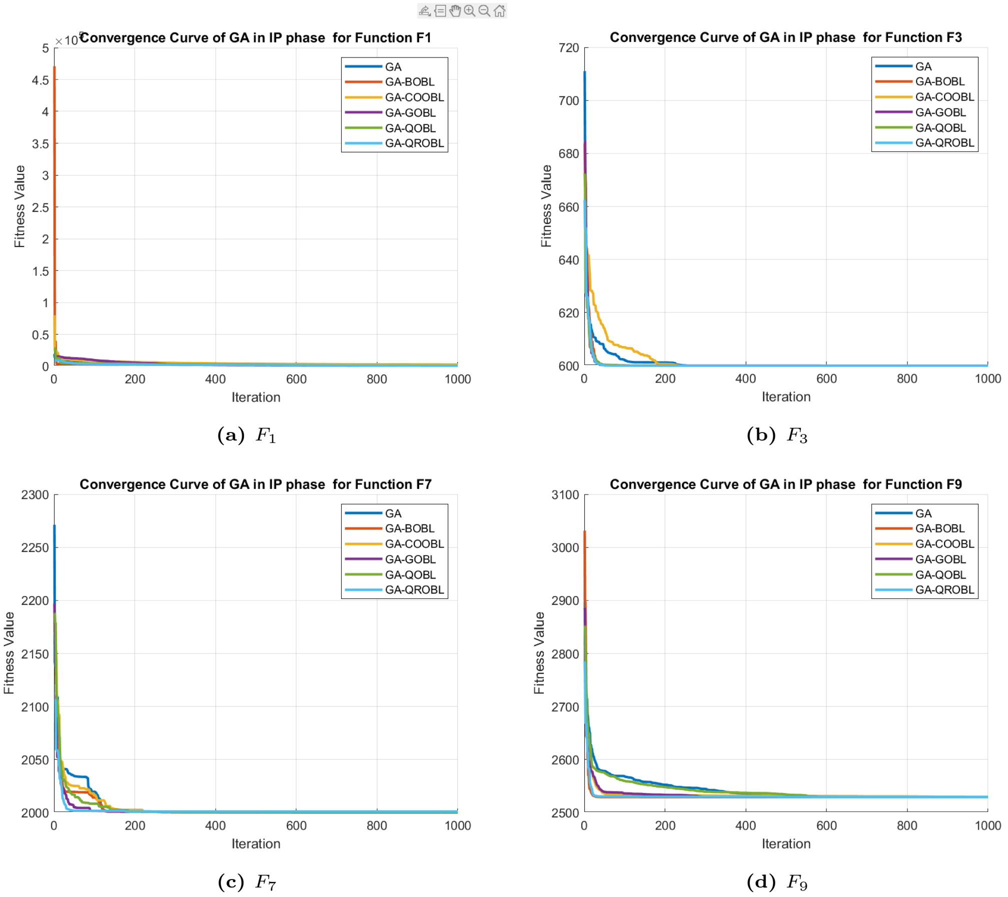

(a) F_1 **(b)** F_3 **(c)** F_7 **(d)** F_9

Fig. 7: Convergence curves of GA and its variant during IP phase ($D = 10$).

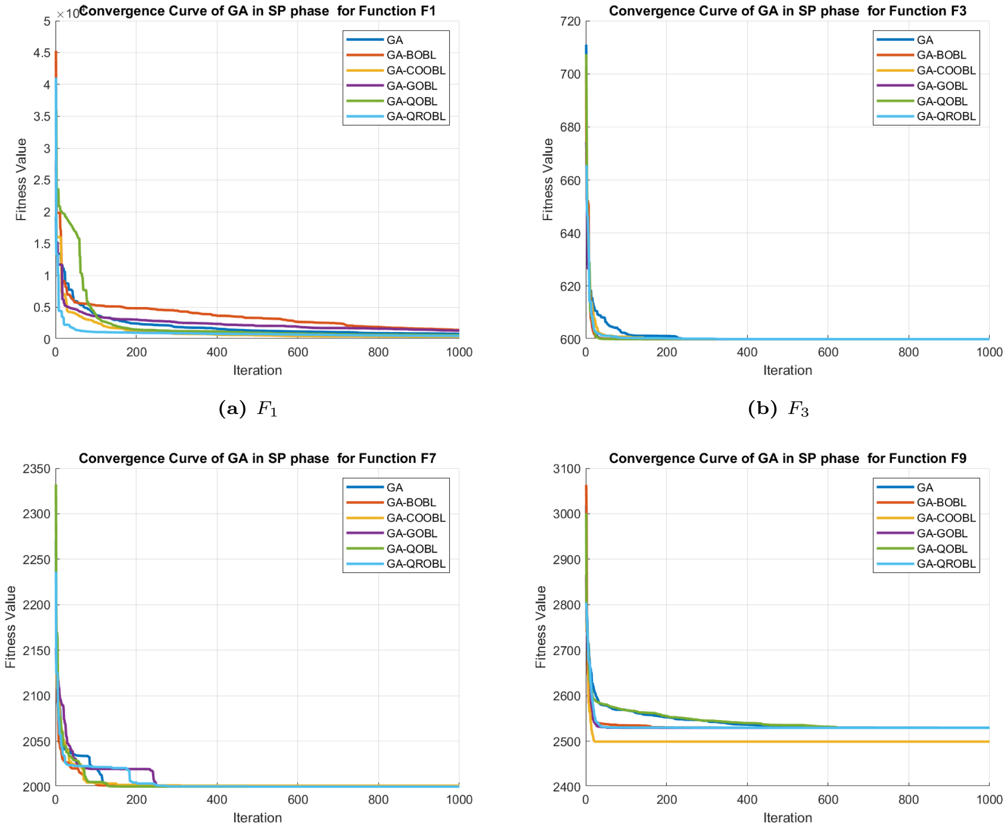

(a) F_1 **(b)** F_3 **(c)** F_7 **(d)** F_9

Fig. 8: Convergence curves of GA and its variant during SP phase ($D = 10$).

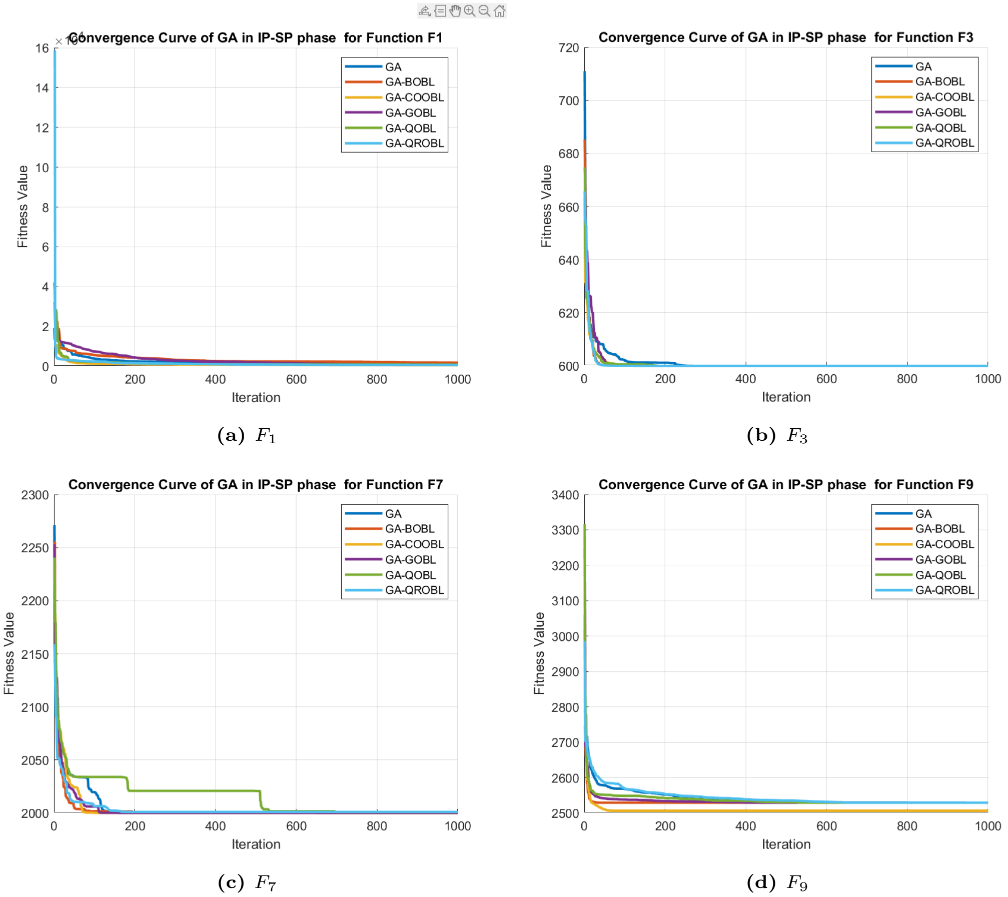

(a) F_1 **(b)** F_3

(c) F_7 **(d)** F_9

Fig. 9: Convergence curves of GA and its variant during IP-SP phase ($D = 10$).

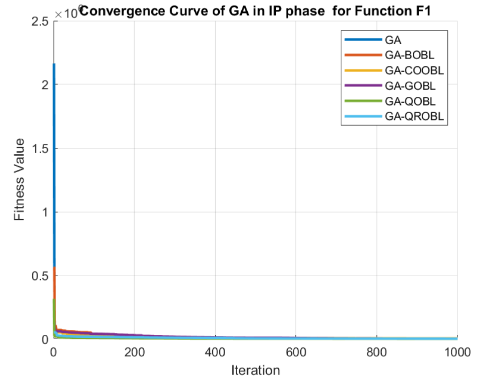

(a) F_1

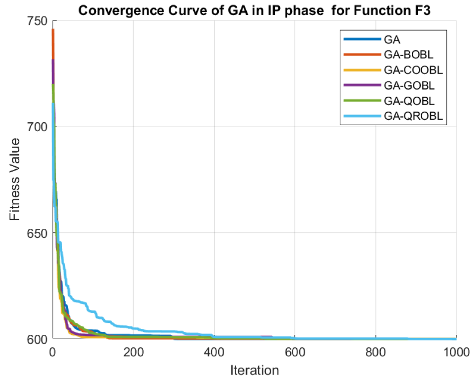

(b) F_3

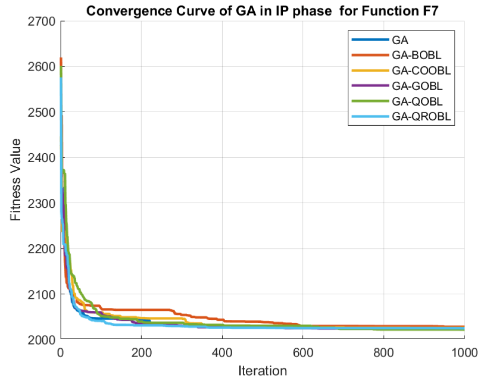

(c) F_7

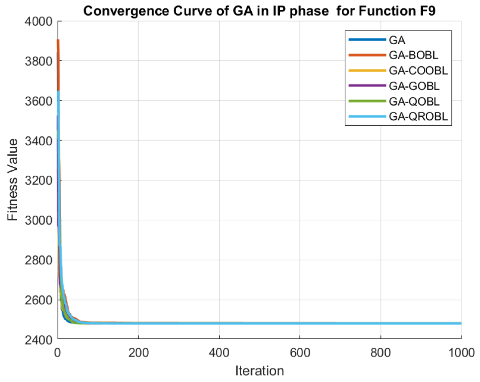

(d) F_9

Fig. 10: Convergence curves of GA and its variant during IP phase ($D = 20$).

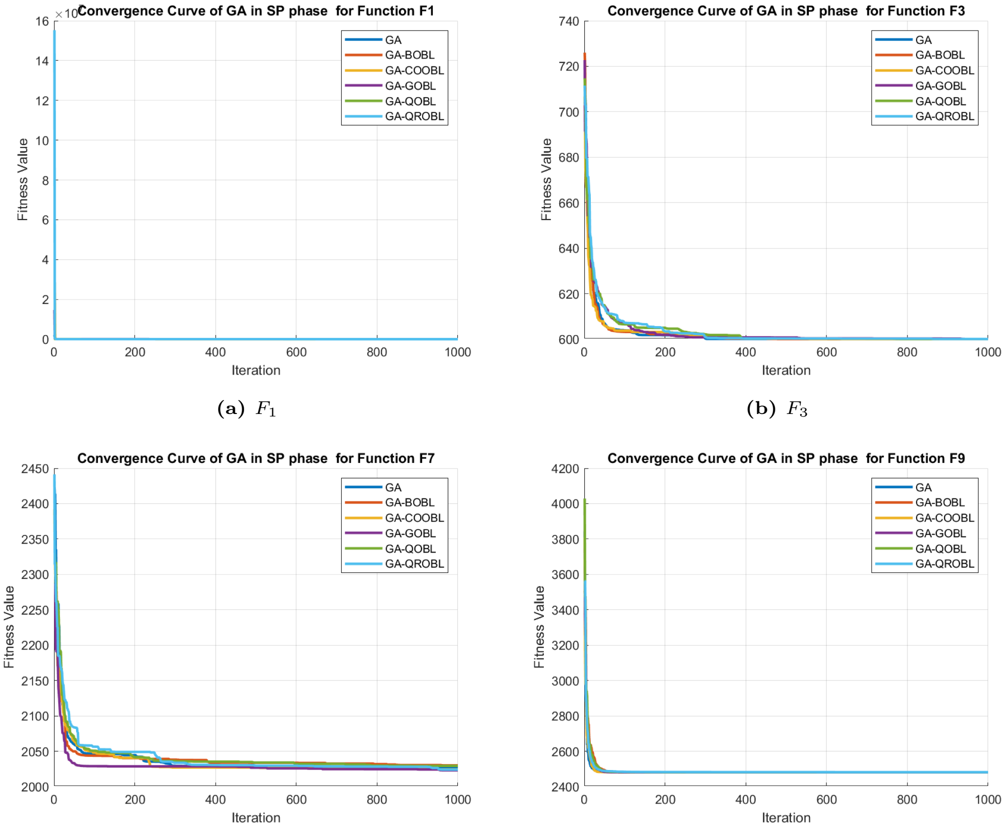

(a) F_1 **(b)** F_3

(c) F_7 **(d)** F_9

Fig. 11: Convergence curves of GA and its variant during SP phase ($D = 20$).

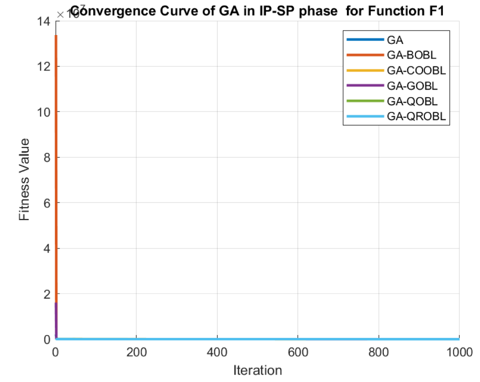

(a) F_1

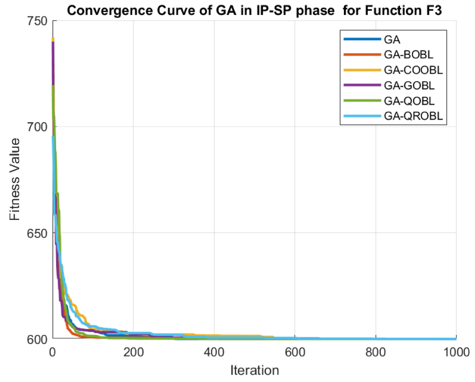

(b) F_3

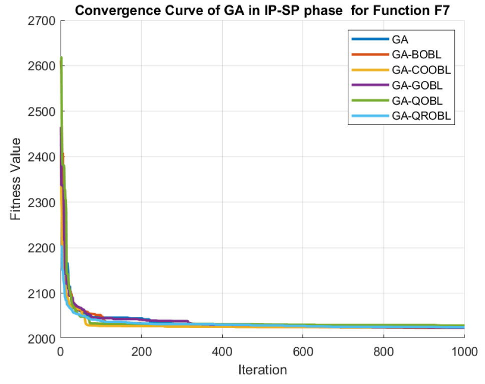

(c) F_7

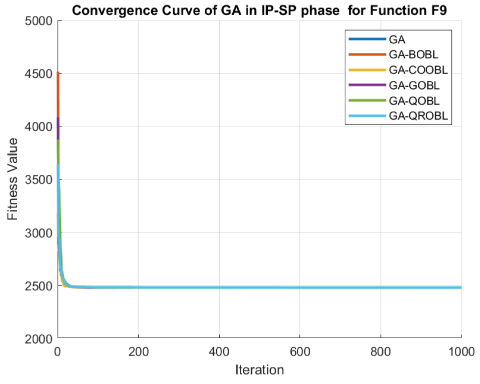

(d) F_9

Fig. 12: Convergence curves of GA and its variant during IP-SP phase ($D = 20$).

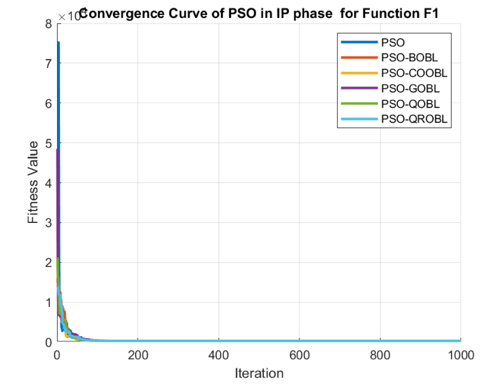

(a) F_1

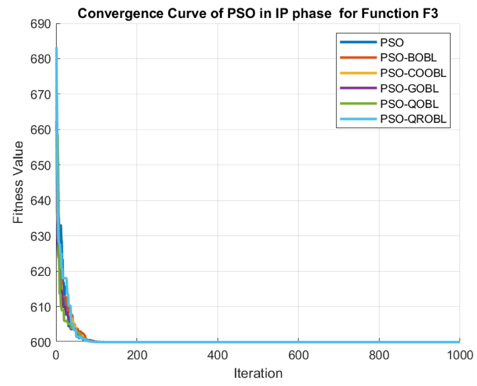

(b) F_3

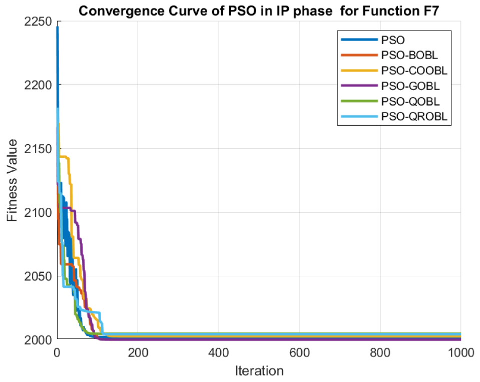

(c) F_7

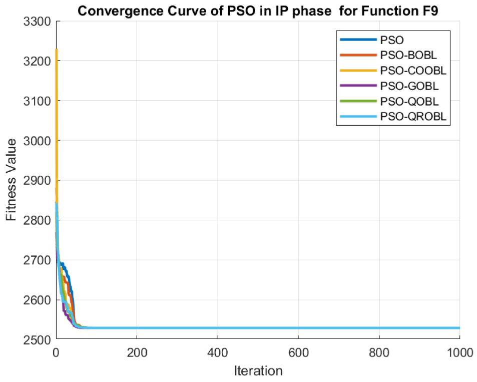

(d) F_9

Fig. 13: Convergence curves of PSO and its variants during IP phase ($D = 10$).

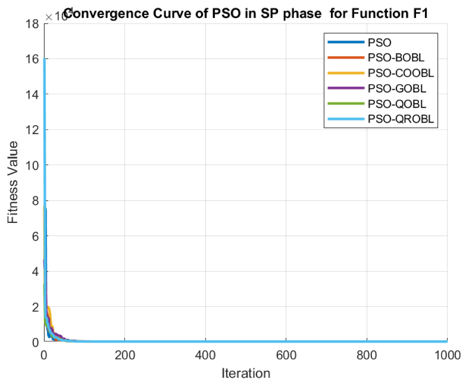

(a) F_1

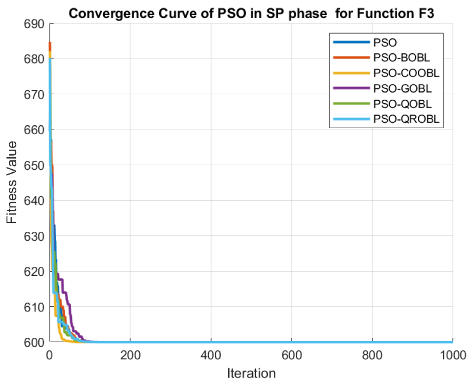

(b) F_3

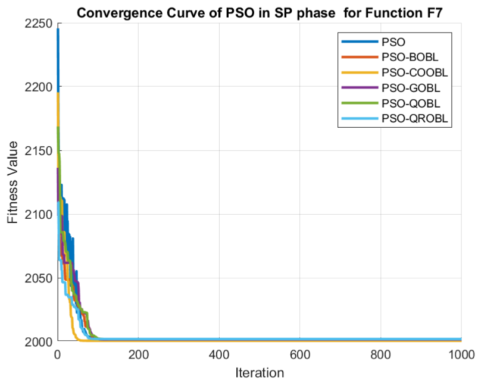

(c) F_7

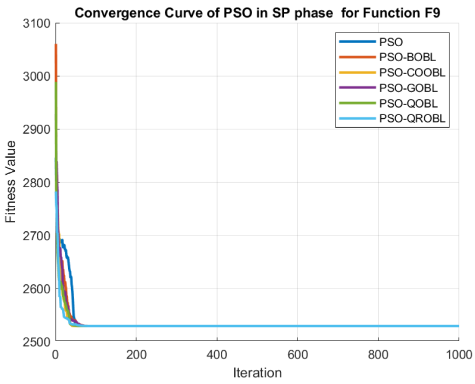

(d) F_9

Fig. 14: Convergence curves of PSO and its variant during SP phase ($D = 10$).

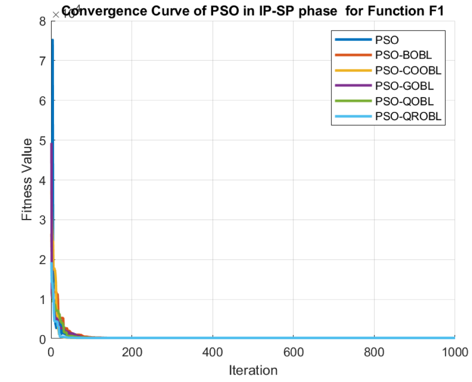

(a) F_1

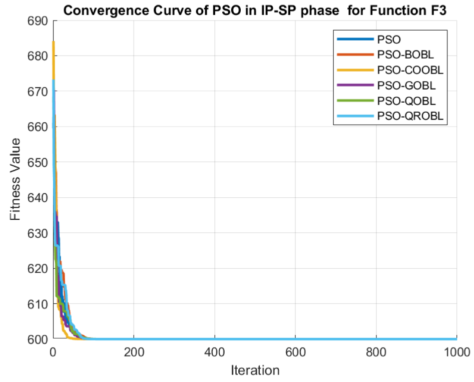

(b) F_3

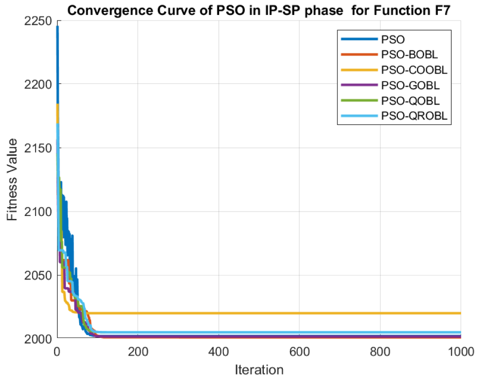

(c) F_7

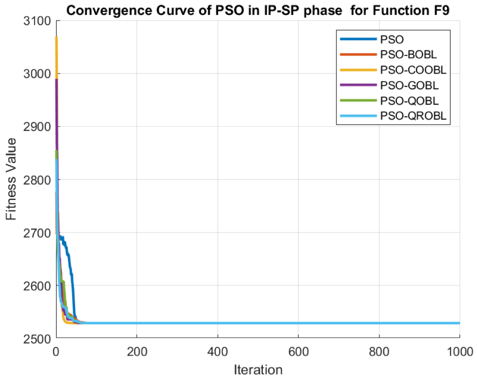

(d) F_9

Fig. 15: Convergence curves of PSO and its variant during IP-SP phase ($D = 10$).

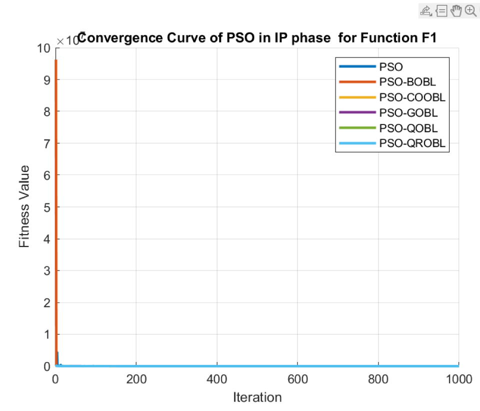

(a) F_1

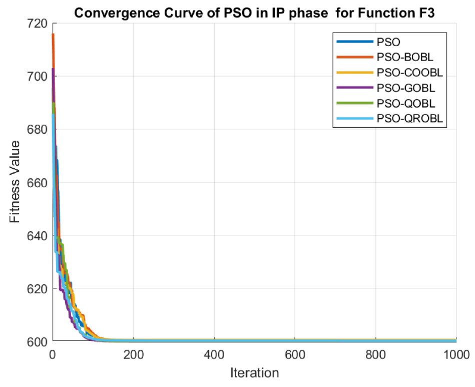

(b) F_3

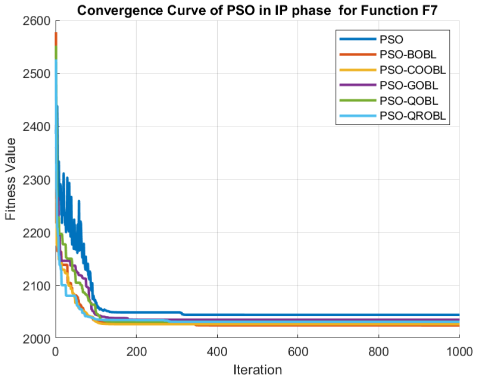

(c) F_7

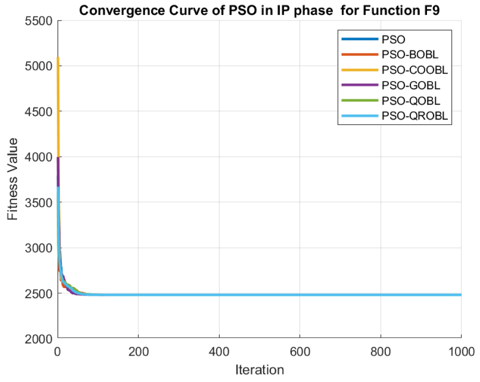

(d) F_9

Fig. 16: Convergence curves of PSO and its variant during IP phase ($D = 20$).

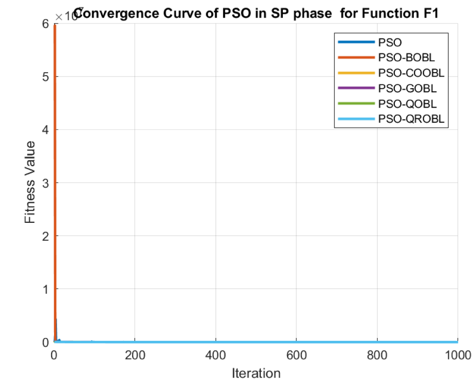

(a) F_1

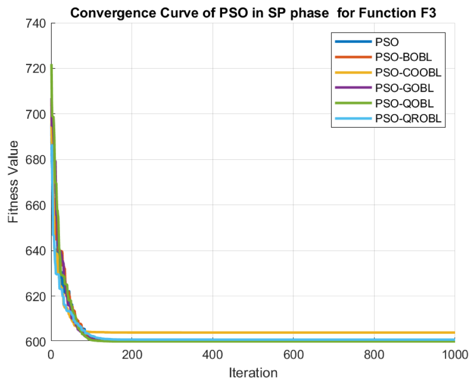

(b) F_3

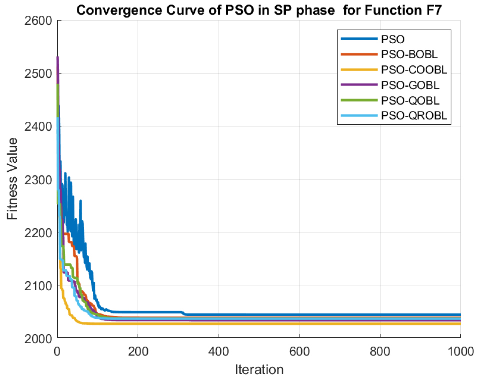

(c) F_7

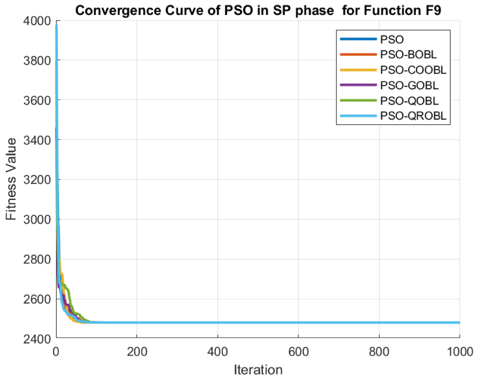

(d) F_9

Fig. 17: Convergence curves of PSO and its variant during SP phase ($D = 20$).

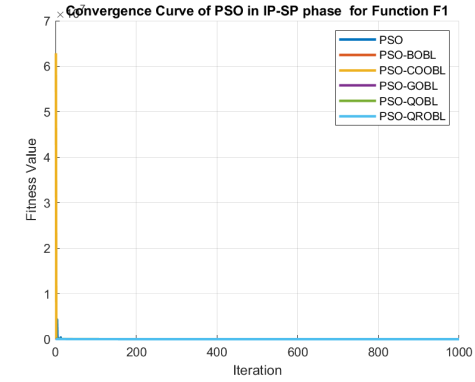

(a) F_1

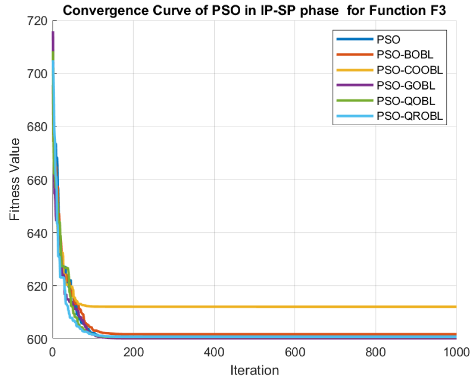

(b) F_3

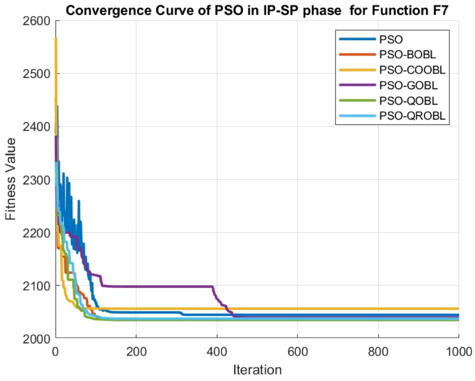

(c) F_7

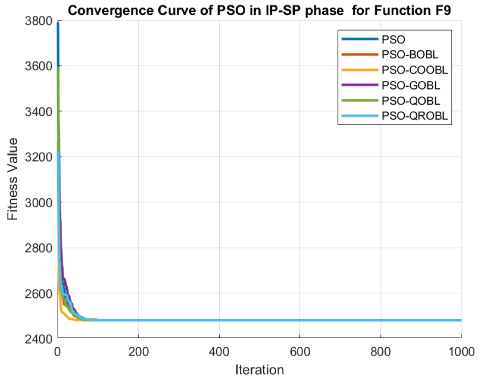

(d) F_9

Fig. 18: Convergence curves of PSO and its variant during IP-SP phase ($D = 20$).

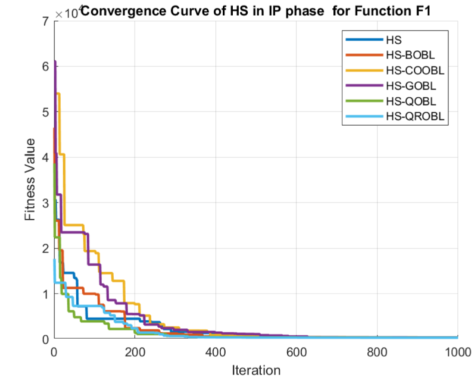

(a) F_1

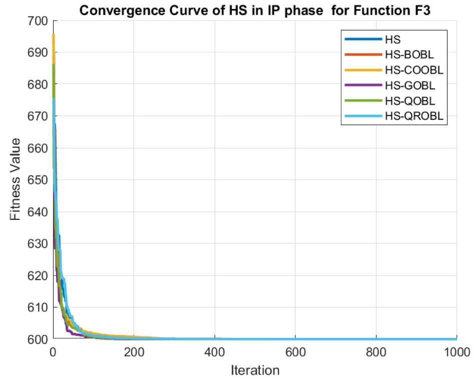

(b) F_3

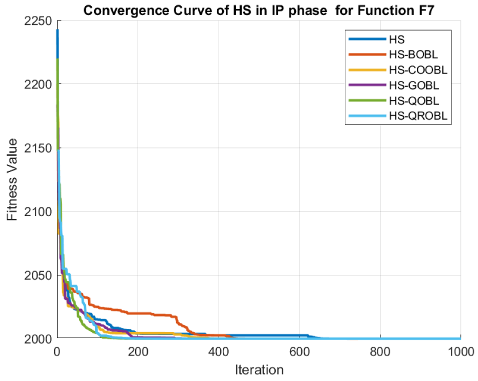

(c) F_7

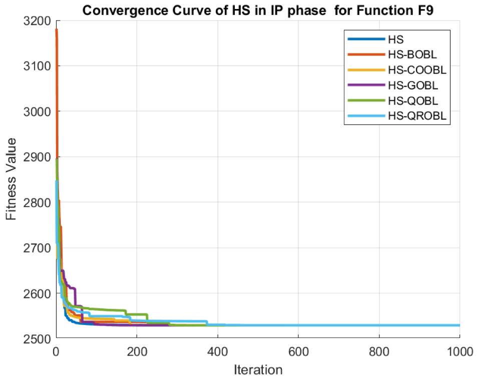

(d) F_9

Fig. 19: Convergence curves of HS and its variant during IP phase ($D = 10$).

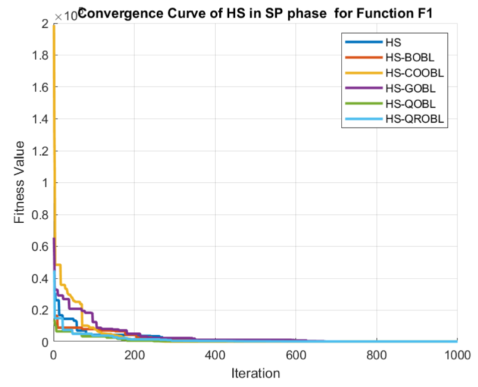

(a) F_1

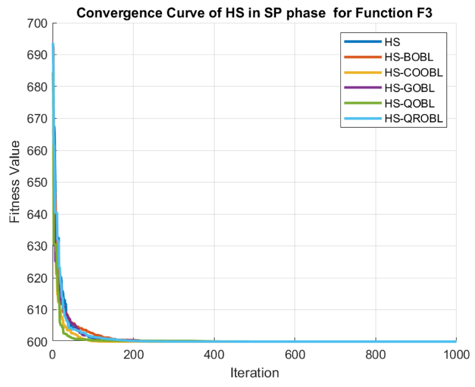

(b) F_3

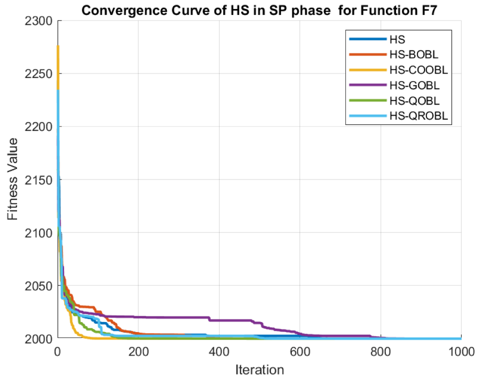

(c) F_7

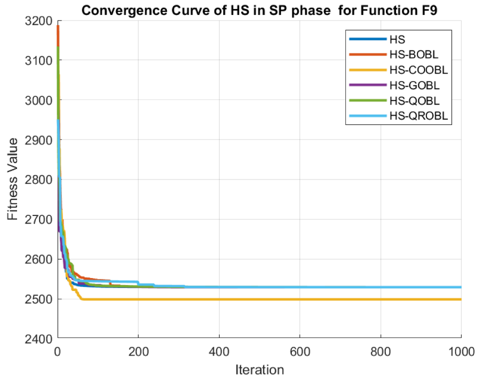

(d) F_9

Fig. 20: Convergence curves of HS and its variant during SP phase ($D = 10$).

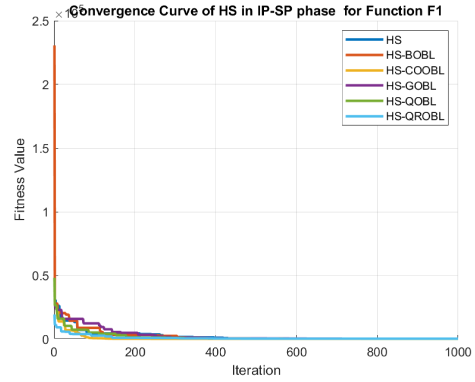

(a) F_1

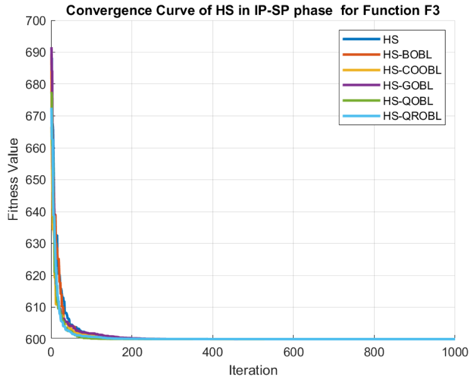

(b) F_3

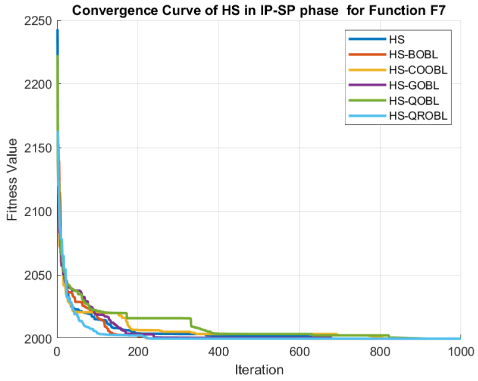

(c) F_7

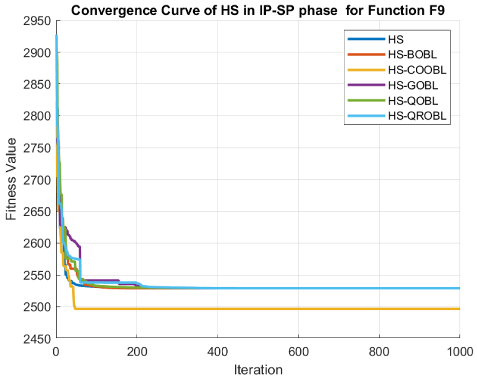

(d) F_9

Fig. 21: Convergence curves of HS and its variant during IP-SP phase ($D = 10$).

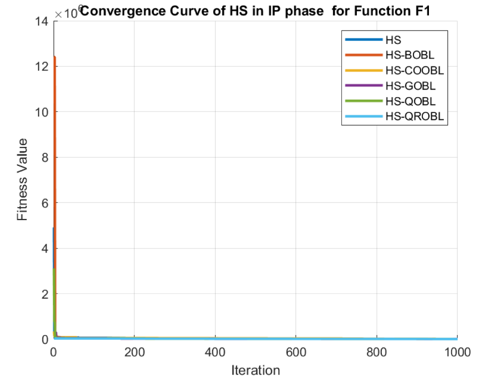

(a) F_1

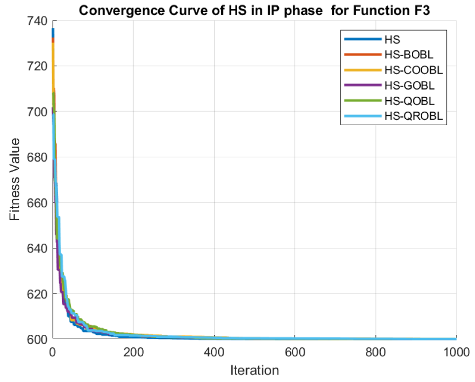

(b) F_3

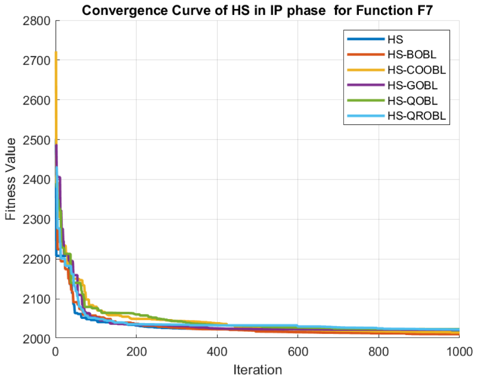

(c) F_7

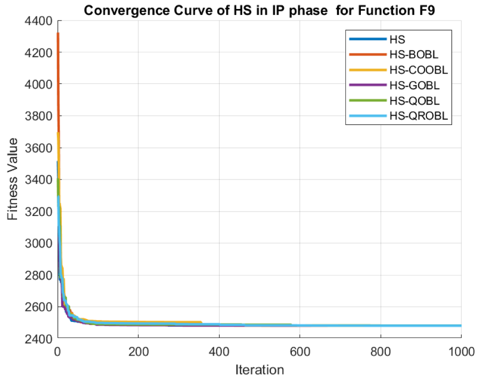

(d) F_9

Fig. 22: Convergence curves of HS and its variant during IP phase ($D = 20$).

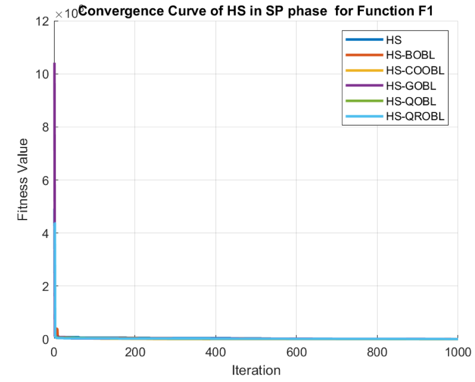

(a) F_1

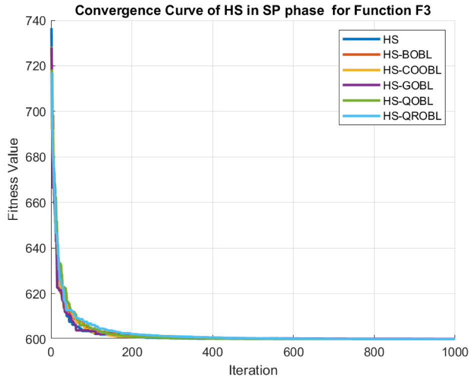

(b) F_3

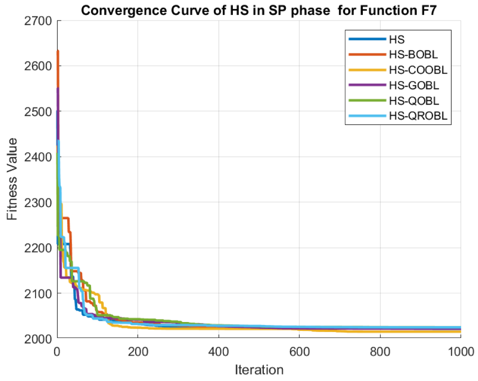

(c) F_7

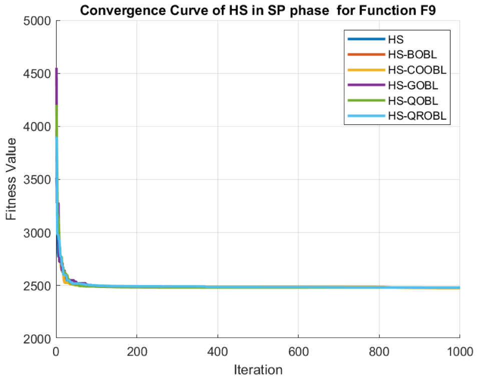

(d) F_9

Fig. 23: Convergence curves of HS and its variant during SP phase ($D = 20$).

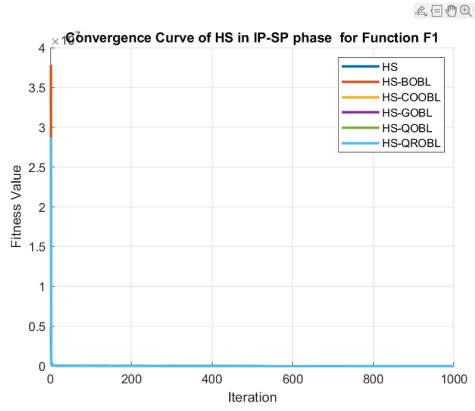

(a) F_1

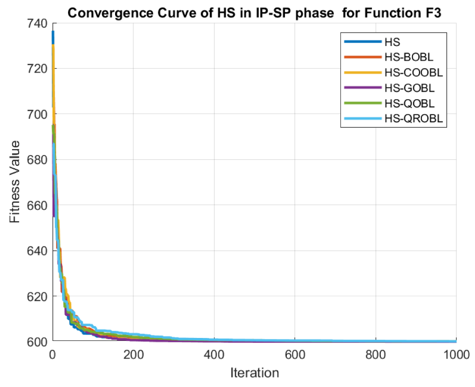

(b) F_3

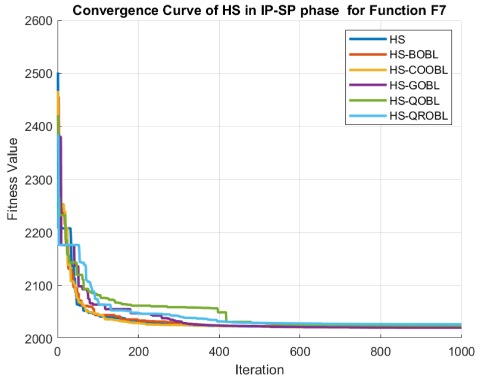

(c) F_7

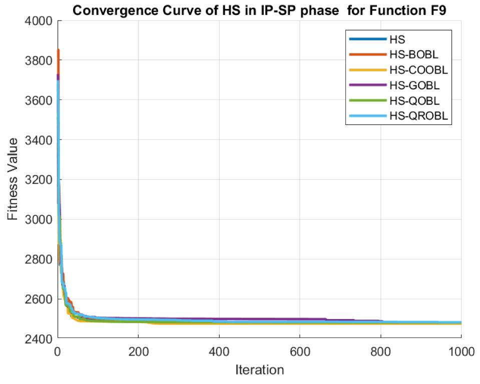

(d) F_9

Fig. 24: Convergence curves of HS and its variant during IP-SP phase ($D = 20$).

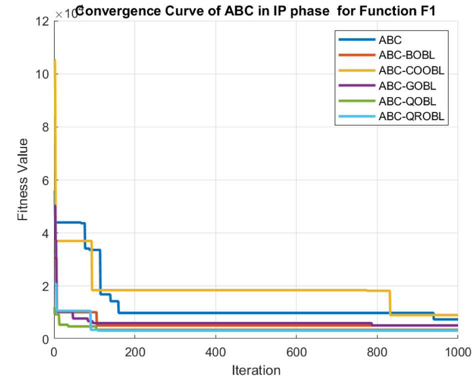

(a) F_1

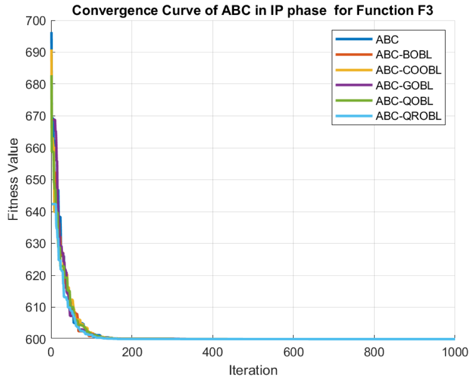

(b) F_3

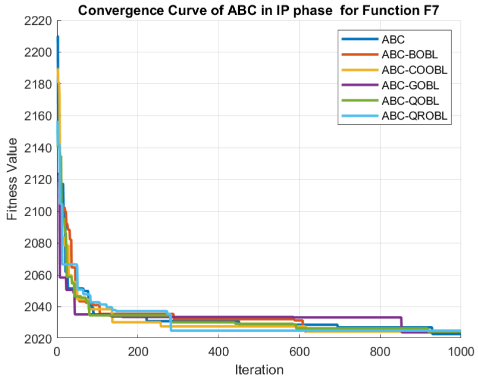

(c) F_7

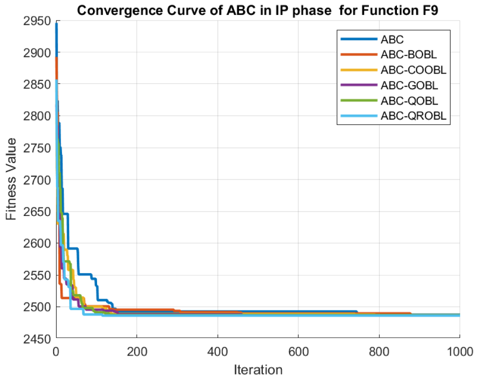

(d) F_9

Fig. 25: Convergence curves of ABC and its variant during IP phase ($D = 10$).

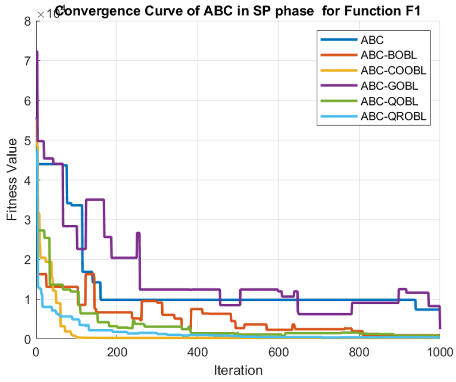

(a) F_1

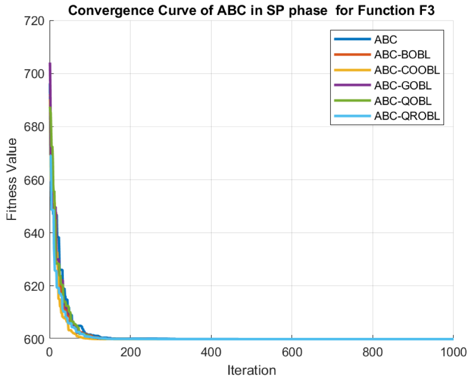

(b) F_3

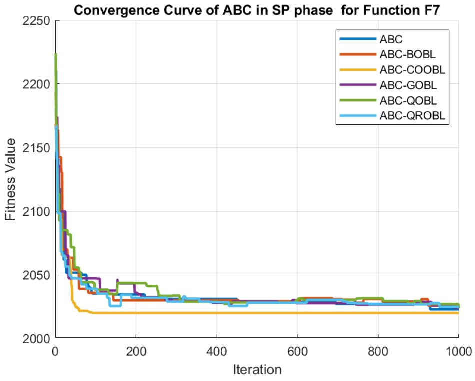

(c) F_7

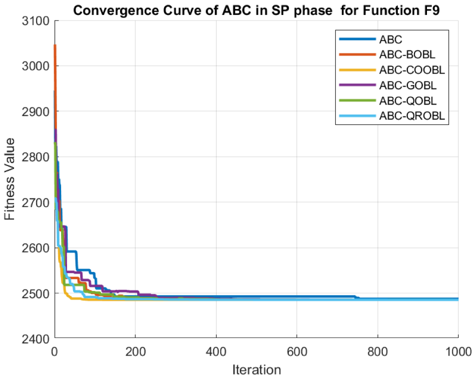

(d) F_9

Fig. 26: Convergence curves of ABC and its variant during SP phase ($D = 10$).

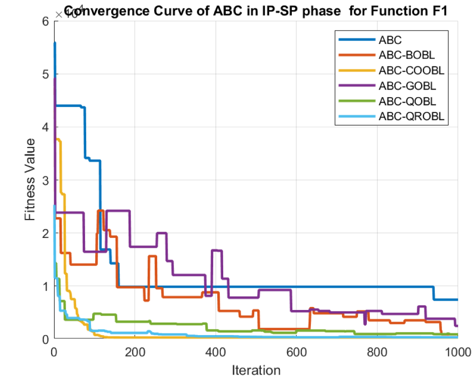

(a) F_1

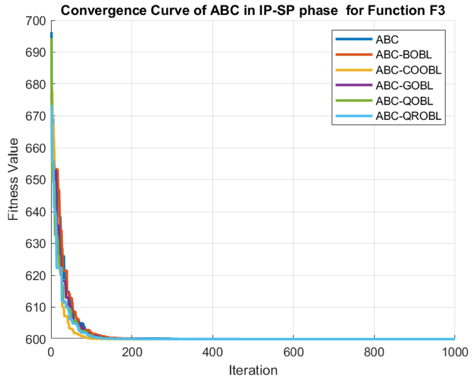

(b) F_3

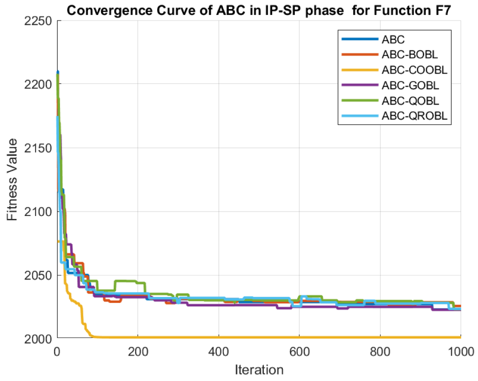

(c) F_7

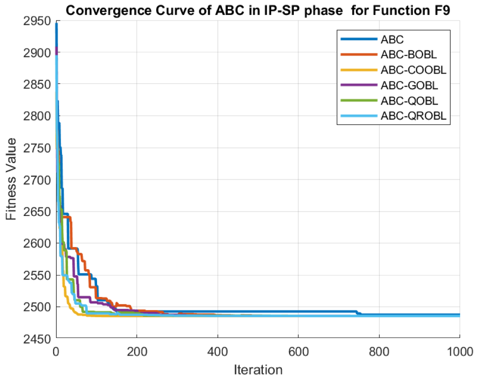

(d) F_9

Fig. 27: Convergence curves of ABC and its variant during IP-SP phase ($D = 10$).

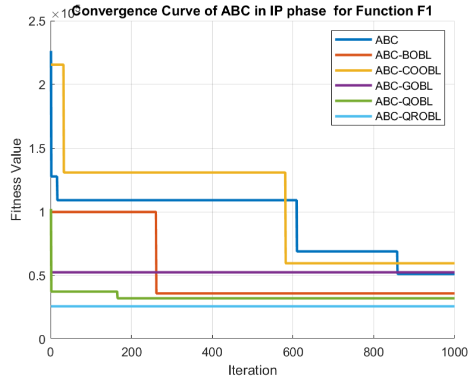

(a) F_1

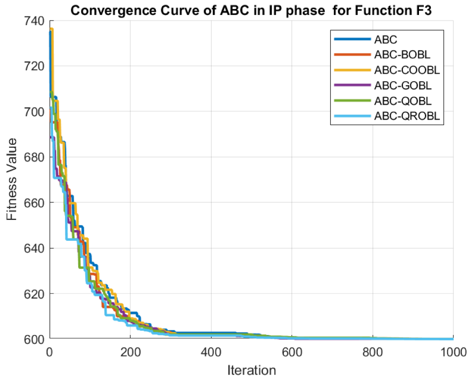

(b) F_3

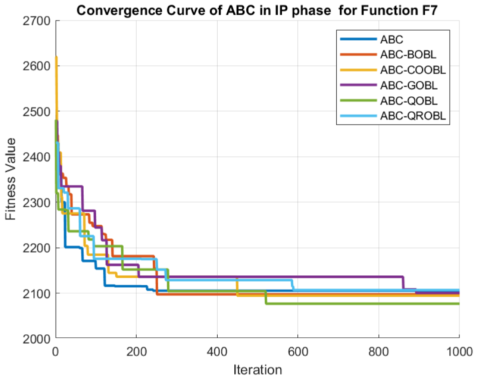

(c) F_7

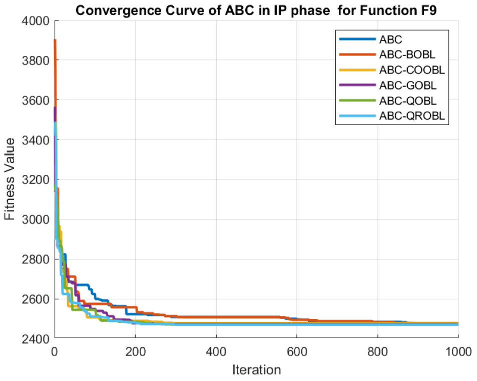

(d) F_9

Fig. 28: Convergence curves of ABC and its variant during IP phase ($D = 20$).

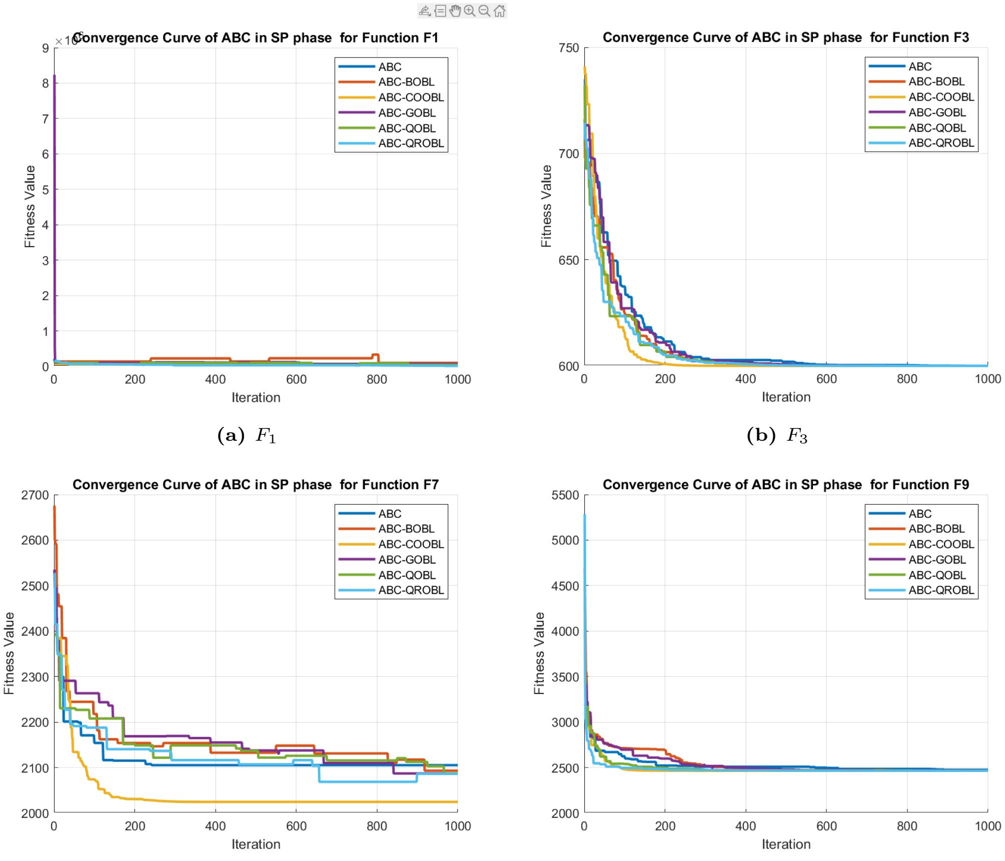

(a) F_1

(b) F_3

(c) F_7

(d) F_9

Fig. 29: Convergence curves of ABC and its variant during SP phase ($D = 20$).

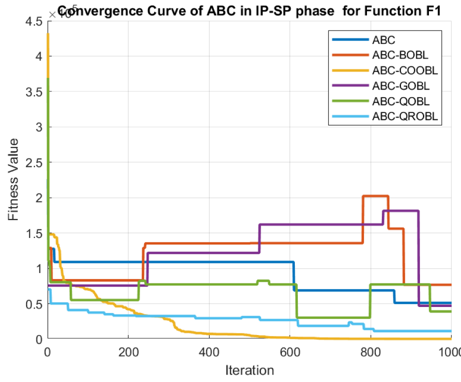

(a) F_1

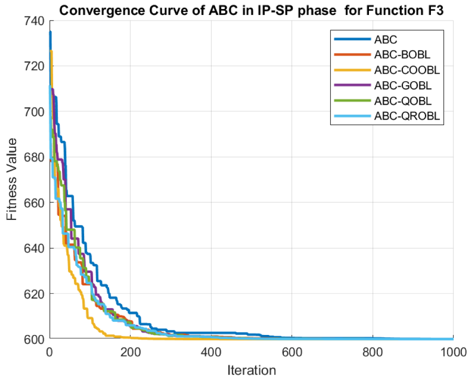

(b) F_3

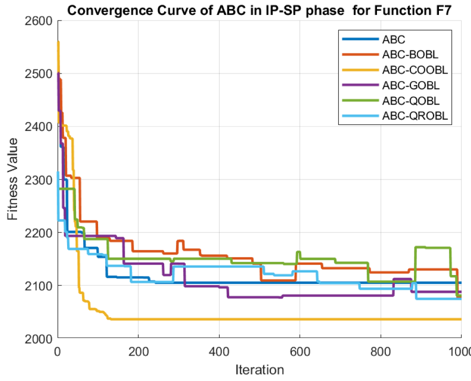

(c) F_7

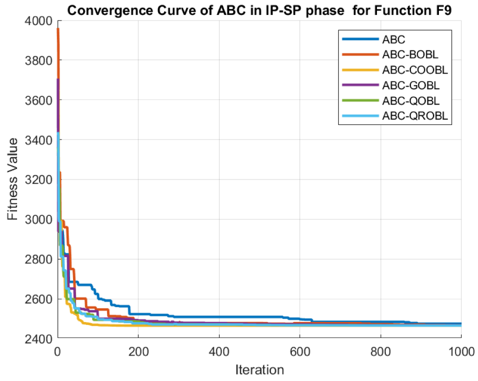

(d) F_9

Fig. 30: Convergence curves of ABC and its variant during IP-SP phase ($D = 20$).
